# 23rd International Symposium on Infections in the Critically Ill Patient

**DOI:** 10.3390/medsci6010013

**Published:** 2018-02-08

**Authors:** Antonio Artigas, Jean Carlet, Ignacio Martin-Loeches, Michael Niederman, Antoni Torres

**Affiliations:** 1 Corporacion Sanitaria Universitaria Parc Tauli, CIBER Enfermedades Respiratorias, Autonomous University of Barcelona, Sabadell, Spain; 2 President of the World Alliance against Antibiotic Resistance (WAAAR), Paris, France; 3 St Jame’s Hospital, Trinity Centre for Health Sciences, Dublin, Ireland; 4 Division of Pulmonary and Critical care Medecine, New York Presbyterian Hospital, Weill Cornell Medical College, USA; 5 Pulmonology department, Clinic Hospital, University of Barcelona, Barcelona, Spain; CIBER Enfermedades Respiratorias, Spain


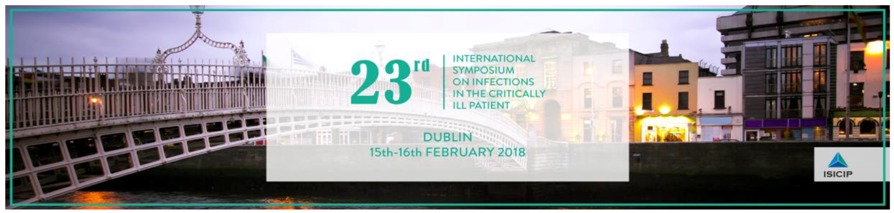


## Introduction

This 23rd International Symposium on Infections in the Critically Ill Patient aims to review current concepts, technology and present advances in infections in critically ill patient.

Sepsis, Pulmonary Infections and their therapeutic and preventive strategies will be the topics presented by international experts who will review and update sepsis as a global international problem. New guidelines of Surviving Sepsis campaign, fluid therapy and vassopressors, a balance view between personalization and protocol treatment and new recommendations for the design of future randomized control trials are provided. The immune response and the emerging methods to personalize sepsis care including new biomarkers and immunomonitoring of patients with sepsis represent a new complementary view to treat patients with severe infections and organ failure in addition to early antibiotic and the control of source of infection.

New ways to treat pulmonary infections including the new global guidelines and the international actions against multiresistant microorganisms and the development of new antibiotics represent key factors to improve the outcome of severe infections.
**Ignacio Martin-Loeches, MD****Antonio Artigas, MD**St James’s Hospital, Trinity Centre for Health Sciences, Dublin, IrelandCorporació Sanitaria Universitaria Parc Tauli, Autonomous University of Barcelona, Sabadell, Spain

## Abstracts Speakers

                                       

 

SESSION I. “SEPSIS: A GLOBAL PROBLEM”

 

## Balancing Personalization and Protocol in Sepsis

SingerMervynUniversity College, London, UK

**Abstract:** Outcome improvement in sepsis depends on early identification followed by appropriate and timely interventions aimed at (i) resuscitating hypoperfused organs, (ii) eradicating the underlying infectious source, (iii) providing adequate but non-injurious organ support until the affected organs recover functionality, (iv) preventing downstream complications and (v) offering adequate but non-excessive rehabilitation. How rigid we should be in dictating practice is, however, very much open to question. No specific protocol has been formally identified as life-saving in sepsis. Components of ‘must-do’ protocols have been abandoned when larger RCTs have failed to replicate the original studies—the trilogy of Early Goal-Directed Therapy studies being a case in point. Positive results are often derived from retrospective, heavily adjusted analyses of databases that are often biologically implausible and not borne out by prospective RCTs. A dogmatic, didactic approach to immediate antibiotics, fluid resuscitation, blood pressure targets and so forth fails to account for the individualized needs of the patient. Sepsis is not a specific disease entity but an umbrella syndrome covering a range of biological phenotypes. Therapies should thus be selected and dose-adjusted accordingly, taking into account the individual host’s phenotype, underlying comorbidities, and the site and source of infection. A one-size-fits-all, brain-stem, heavily protocolized approach will suit the lowest common denominator. Surely we should seek loftier ambitions based on education, an increased depth of understanding, enhanced monitoring and diagnostics, and a personalized approach incorporating evidence and clinical expertise. This concept of EBID (evidence-based individualized decision-making) is not new; indeed, it was enshrined by none other than David Sackett, a pioneer of evidence-based medicine who railed against rigid adoption of evidence: “Clinicians who fear top down cookbooks will find the advocates of evidence -based medicine joining them at the barricades”.

## Do We Need New Clinical Design for RCT?

VincentJean-LouisDepartment of Intensive Care, Erasme Hospital, Université libre de Bruxelles, Brussels, Belgium

**Abstract:** The prospective randomized controlled trial (RCT) has been widely promoted as the only effective means of definitively proving the superiority of one intervention over another. However, in the intensive care unit (ICU) population, RCTs can be challenging to conduct, and results have rarely demonstrated beneficial effects of the intervention under investigation on outcomes. The main reason for this lack of success is the heterogeneous nature of the critically ill populations included in these studies. Ethical issues can also be a problem in the ICU as patients are frequently not able to give consent and surrogates may not be available in the immediate phase, which is often when interventions are most likely to be effective and randomization therefore needs to occur. As a result of these and other limitations, new clinical trial designs have been suggested including so-called adaptive designs. These provide some flexibility by allowing the investigator to adjust certain criteria after the trial has started so that the study is more likely to identify a benefit of the studied treatment without undermining the underlying integrity of the study. Such changes in design are made prospectively based on analysis of data obtained from the ongoing trial. Some adaptive designs are already widely used in RCT design, including early stopping for futility or safety. However, other approaches, including re-estimating calculated sample sizes according to results of interim analyses and eliminating certain arms of a trial if treatment is only effective in some subgroups of patients, have not been widely used. Although in theory adaptive designs, perhaps particularly response-adaptive population enrichment designs, could be useful in the ICU setting, we are still faced with problems of patient heterogeneity and need to be able to better identify patients who are likely to respond to therapies before we can start using this approach. For example, we currently have no individual biomarkers able to accurately identify patients with sepsis or how they will respond to therapy. As such, most clinical trials include all patients with sepsis regardless of their immune status, age, sex, infecting organism, comorbid status, and so on, so that some will inevitably respond to the treatment being assessed and others will not, giving an overall negative trial result. An adaptive population-enrichment design could help in this context, but what markers should be used to create patient subgroups to evaluate response? Importantly too, the statistical analyses involved in such studies are considerably more complex than in the classical RCT format. We should also make greater use of observational studies in the ICU population. These enable assessment of effects of interventions in all patients without the often restrictive inclusion and exclusion criteria of the RCT, and without waiting for informed consent, which may not even be granted. The results of such studies could help identify which patients are most likely to respond to therapies and thus inform RCT inclusion criteria so that treatments could be targeted more appropriately in a personalized, precision medicine approach to trial design.

**Suggested Reading**

1. Bhatt, D.L.; Mehta, C. Adaptive designs for clinical trials. *N. Engl. J. Med.* **2016**, *375*, 65–74.
2. Huskins, W.C.; Fowler, V.G., Jr.; Evans, S. Adaptive designs for clinical trials: Application to healthcare epidemiology research. *Clin. Infect. Dis.* **2017**, doi:10.1093/cid/cix907.
3. Vincent, J.L. We should abandon randomized controlled trials in the intensive care unit. *Crit. Care Med.* **2010**, *38* (Suppl. 10), S534–S538.
4. Ospina-Tascón, G.A.; Büchele, G.L.; Vincent, J.L. Multicenter, randomized, controlled trials evaluating mortality in intensive care: doomed to fail? *Crit. Care Med.* **2008**, *36*, 1311–1322.
5. Vincent, J.L. Improved survival in critically ill patients: Are large RCTs more useful than personalized medicine? No. *Intensive Care Med.* **2016**, *42*, 1778–1780.

##                                        

 

SESSION II. “FLUID THERAPY AND VASSOPRESSORS: UNRESOLVED ISSUES”

 

## Choice of Fluids in the ICU. The Best i.v. Fluid?

MartinClaudeDepartment of Anesthesia, Intensive Care and Trauma Center, Nord University Hospital.

**Abstract:**

**Introduction**

Fluid resuscitation relieves overt tissue hypoxia. Underresuscitation contributes to aggravate oxygen deficit, and over-aggressive resuscitation with fluids has deleterious effects on patient outcome:

- Development of pulmonary edema and/or abdominal compartment syndrome.
- Increased incidence of acute kidney injury (AKI).
- Less ventilator free days and increased ICU stay
- Increased mortality

The breaking point is unclear between an adequate fluid resuscitation and an over-aggressive resuscitation leading to complications.

Although the need for the use of fluid is accepted in many patients, the choice of fluid therapy remains controversial. Cristalloids are characterized by various salt concentrations of saline solutions (mostly 0.9%, but also 0.45, 3% …) and the balanced salt solutions (Hartmans, Ringer lactate, Plasma-Lyte, Isofundine …). These drugs are cheap, expand the intra and extravascular fluid compartments. They improve cardiac output (CO) and end-organ perfusion.

The colloids include human albumin, gelatins, hydroxyethyl starch (HES) and dextran containing fluids. They expand fluid compartments and oncotic pressure. These drugs are expansive, may carry a risk of AKI (HES mostly) and have a risk of anaphylactoid reactions. Their superiority in ICU patients has not been demonstrated yet in terms of pulmonary oedema, resolution of organ failures, and mortality.

**Fluid Responsiveness and Success of Fluid Resuscitation**

The most common error in ICU patients is to believe that fluid resuscitation is successful when a number is reached. Never treat a number but treat a patient. The aim is to restore end-organ perfusion in a patient suspected to be fluid responsive, and to carefully weight the risk/benefit ratio of a single additional drop of fluid. Several surrogate markers of organ perfusion can be used. A combination of several (all?) should be considered.

- Normal capillary refill
- Increase in CO
- Increase in urine flow
- Increase in mean arterial
- Decrease in serum lactate after 4/6 h
- Normalization of central venous blood oxygen saturation (ScvO2)
- Improvement of microcirculation perfusion abnormalities (to be evaluated by further studies)

The prediction of preload responsiveness is not an easy task and there is not gold-standard for that. All parameters and tests have limitation in terms of sensitivity and specificity.

Static measures can no longer be recommended. The followings do not reliably reflect the left ventricular filling pressure in shock states with pulmonary hypertension or compliance changes:

Central venous pressure.
Pulmonary occlusion pressure.
Ventricular end-diastolic volumes.
Ventricular diastolic area.
Global end-diastolic volume.
Intrathoracic blood volume.

Dynamic measures of fluid responsiveness are recommended instead. Table 1 and Table 2 present the parameters to use and the main limitations in their interpretations. Ultrasound are useful to reduce the time of diagnostic uncertainty and guide resuscitation. They may be used to assess volume status, ventricular strain, myocardial dysfunction. Passive leg raising test can be used to predict volume responsiveness without giving a single drop of fluid. It should be titrated against changes in CO/stroke volume rather than pulse pressure changes.

**Types of Fluids in Sepsis**

The need for fluid resuscitation is widely accepted for fluid resuscitation in septic patients, but the choice of the “best” fluid(s) is still controversial.

Crystalloids

“Normal” or “Physiological” Saline, or “Unbalanced” Saline

The 0.9% saline solution is neither normal nor physiological. The sodium concentration exceeds that of the plasma (154 mEq/L). From experimental studies it is well demonstrated that high renal tubular chloride concentration cause significant damages in renal hemodynamics. The renal blood flow is decreased in response to a renal afferent vasoconstriction. There is no such changes in correlation to sodium concentration. In case of associated hypovolemia the reduction in renal blood flow is twice as high as in euvolemic dogs. In healthy volunteers intravenous 0.9% saline led to a decrease in renal blood flow velocity and renal cortical tissue perfusion as compared to plasma-lyte 148.

Metabolic acidosis is a major complication in septic patients due to lactic acidosis. Use of 0.9% saline is associated with an additional iatrogenic hyperchloremic acidosis. The clinical importance of this acidosis in unclear.

“Balanced Crystalloids”

As compared to 0.9% saline, balanced solutions have chloride concentration more closely approximating plasma but osmolality is lower:

- Lactated Ringer’s: Cl—109 mEq/L, osmolality 277 mOsm/L.
- Ringer’s acetate: Cl—127 mEq/L, osmolality 277 mOsm/L.
- Plasma-Lyte 148; Cl—93 mEq/L, osmolality 294 mOsm/L.

These drugs also contain anions such as lactate, gluconate or acetate in non-physiological concentrations. There is an increasing use of balanced solutions in ICU and septic patients. Contractory conclusions come from meta-analysis and observational studies comparing high versus low-chloride concentrations. Reduced mortality and less AKI are suggested following the use of balanced solutions, but patients are not always in septic shock or even septic. During septic shock, aggressive volume resuscitation in needed and the type of crystalloids may not influence the clinical outcome.

Two large-scale randomized controlled trials (RCT) are currently conducted. They will compare a gluconate/acetate-buffered crystalloid with 0.9% saline (The Brazilian study will include 11,000 patients and the Australian/New-Zealand study 8800 patients). The primary outcome will be all-cause mortality at 90 days. For the time being balanced solutions can be recommended, lactated-buffered fluids being cheaper and more widely available than acetate/gluconate-buffered fluids.

Colloids. HES Cannot Be Used in Critically Ill Patients, at Least in Europe and North America

Gelatins

They are considered less harmful to the renal system and coagulation factors than HESs. Currently a novel RCT is investigating the use of gelatin opposed to crystalloid administration in septic patients (NCT02715466).

Albumin

It is the main determinant of plasma oncotic pressure and has a pivotal role in the regulation of fluid dynamics at the microvascular level. In addition other properties may be relevant for septic patients: stabilization of glycocalyx, transport of molecules, antioxydant, immuno-modulating effects, positive inotropic effects …

The SAFE trial (albumin versus 0.9% saline) was negative, but a subgroup analysis was performed in septic patients (a priori) and the adjusted odds ratio for death was 0.71 (95% CI: 0.52–0.97, *p* = 0.03) for albumin. The CRISTAL trial compared various colloids (including albumin) to crystalloids and the 90-day mortality was lower in patients receiving colloids. The ALBIOS trial was negative for the global population. However, in patients with septic shock the albumin patients had a 6.3% higher survival at 90 days (*p* = 0.04). A meta-analysis, among others, including the EARSS trial (available as an abstract) concluded that mortality was lower among patients receiving albumin, relative risk ranging from 0.87 to 0.94. Currently, albeit some date come from subgroup analysis or meta-analysis, there is indirect, but convincing evidence that albumin provides some improvement in morbidity and survival in septic shock patients.

## How Much Fluid?

ArtigasAntonioCorporación Sanitaria Universitaria Parc Tauli, CIBER Enfermedades Respiratorias, Autonomous University of Barcelona, Sabadell, Spain

**Abstract:** Adequate initial fluid resuscitation of septic shock followed by a conservative late fluid management strategy has been associated with improved survival and reduced of mechanical ventilation and length of ICU stay [1]. CLASSIC trial restricting volumes of resuscitation fluid in adults with septic shock after initial management was associated with a lower incidence of acute kidney injury (AKI) compared with standard care providing rationale for avoiding excessive or unnecessary fluid loading [2]. Accordingly, a close monitoring of fluid balance in acute phases of resuscitation and following stabilization is necessary. Fluid management in critically ill patients, in particular those with established AKI, is a potentially important modifiable factor that may have important interactions with outcome [3]. Balakumar et al. recently demonstrated that both positive and negative fluid balance compared with even were associated with higher 1-year mortality (HR 1.16–1.22, *p* = 0.004) and no association between fluid balance and renal recovery was found [4]. Fluid management in patients with septic shock and acute respiratory distress syndrome (ARDS) also appears to be an important determinant of hospital mortality. Sakr et al. showed that patients with ARDS who survived achieved a net negative fluid balance compared to non-survivors. A positive mean fluid balance was also shown to be an independent predictor od ICU mortality in their investigation [5]. These studies examining fluid balance in septic shock and ARDS individually suggest that improvements in patient survival can be attained if patients improve to the point where optimal fluid management practices are able to be applied. Sicker patients have a greater dysregulated hosy response to infection and increase mortality, in part because the sickest patients commonly suffer the penalties for under-and-over resuscitation with organ ischemia/hypoxia and organ edema respectively, thereby increasing the risk of sepsis-related organ dysfunction [6]. Recently Famous et al. identified two ARDS subphenotypes by a limited number of varaibles, that responed differently to random-assigned fluid management [7]. These findings support the presence of subphenotypes that may require different treatment approaches. Clinical application will require real-time assigment of subphenotypes by rapid biomarkers assays. More recently, in a substudy of Sapphire trial, subphenotypes with a high risk of AKI defined by [TIMP-2]. [IGFBP 7] >2.0 may require a different fluid replacement treatment to improve outcome. The standarization of evidence-based practices in the care of septic shock has become accepted as the optimal method for management [8]. A meta-analysis of nine studies, including 1001 septic patients found lower mortality (OR 0.64; 95%CI 0.43–0.96) in patients treated with an early, targeted resuscitation protocol including fluids, inotropes and vassopressors [9]. Waechter et al. evaluated the association between hospital mortality and administration of intraveneous fluis and vasoactive agents during the first 24 h. they found that hospital mortality was associated with timing and volume fluis and with timing vasoactive agents. The lowest mortality include large volumes of fluids given early, combined with waiting to begin vasoactive agents until the first hour after the onset of shock, indicating that early initiation of vasoconstrictive agents in septic shock may cause harm even in the presence of adequate fluid resuscitation [10]. Three multicenter RCTs (ProMISE, ARISE, ProCESS) [11–13] and PRISM meta-analysis [14] of early goal-directed therapy did not result in better outcomes than usual care and were associated with higher hospitalization costs, across a broad range of patients and hospital characteristics. Policies that mandates routine measurement of central venous pressure (CVP) in all patients with sepsis did not improve outcomes [15]. In septic shock patients, rather than following predifined therapeutic algorithms, it is more reasonable to individualize fluid resuscitation to prevent harmful of fluid overload.After the initial salvage phase, the benefit risk ratio for further fluid infusion should be carefully assessed in each patient. Thus, if CVP is not measured, what clinical metrics should be used to assess how aggressively fluid should be removed? The new Surviving Sepsis Guidelines (SSC) [16] and the European Society of Intensive care Medecine consensus conference [17] recommend the use of dynamic variables of fluid responsiveness instead CVP. A variety of dynamic variables have been proposed [18]: pulse pressure variation, stroke volume variation, collapsibility of the superior vena cava, distensibility of jugular vein. A tidal volume of 8 mL/Kg IBW for one minute and end-espiration occlusion test in patients with mechanical ventilation are recommended to improve the accuracy of fluid response prediction in patients with spontaneous ventilation, arrhytmias, low lung compliance and high frequency ventilation. The passive leg raising test wich is usable in almost all the situations, is a reliable predictor of fluid responsiveness [19]. The current SSC guidelines recommend infusing at least 30 mL/Kg of i.v. crystalloids within the first three hours of resuscitation. The major concern of this recommendation is the arbritary volume and the unreasonably long-time frame of three hours before reassessing the hemodynamic status after fluid resusciation. A rate of 10 mL/Kg over the first hour of resuscitation seems to be more reasonable. In patients with ARDS the benefit-risk ratio of continuing fluid therapy should be carefully assessed by measurement of extravascular lung water (EVLW) and pulmonary vascular permeability index (PVPI) by transpulmonary thermodilution technique wich are independent predictors of severity and mortality in ARDS patients [20].

**Conclusions**

Individualize fluid resuscitation to prevent harmful fluid overload after the initial salvage phase is necessary particularly in septic shock patients with ARDS and AKI.

**References**

1. Murphy, C.V.; Schramm, G.E.; Doherty, J.A.; Reichley, R.M.; Gajic, O.; Afessa, B.; Micek, S.T.; Kollef, M.H. The importance of fluid management in acute lung injury secondary to septic shock. *Chest* **2009**, *136*, 102–109.
2. Hjortrup, P.B.; Haase, N.; Bundgaard, H.; Thomsen, S.L.; Winding, R.; Pettilä, V.; Aaen, A.; Lodahi, D.; Berthelsen, R.E.; Christensen, H.; et al. Restricting volumes of resuscitation fluid in adults with septic shock after initial management: The CLASSIC randomised, parallel-group, multicentre teasibility trial. *Intensive Care Med.* **2016**, *42*, 1695–1705.
3. Silversides, J.A.; Major, E.; Ferguson, A.J.; Mann, E.E.; Mc Auley, D.F.; Marshall, J.C.; Blackwood, B.; Fan, E. Conservative fluid management or deresuscitation for patients with sepsis or acute respiratory distress syndrome following the resuscitation phase of critical illness: A systematic review and meta-analysis. *Intensive Care Med.* **2017**, *43*, 155–170.
4. Balakumar, V.; Murugan, R.; Sileann, F.E.; Palevsky, P.; Clermont, G.; Kellum, J.A. Both positive and negative fluid balance may be associated with reduced long-term survival in the ritically ill. *Crit. Care Med.* **2017**, *45*, e749–e757.
5. Sakr, Y.; Vincent, J.L.; Reinhart, K.; Greneved, J.; Michalopoulos, A.; Sprung, C.L.; Artigas, A.; Ranieri, V.M.; SEPSIS Occurence in Acute Ill Patients Investigators. High tidal volume and positive fluid balance are associated with worse outcome in acute lung injury. *Chest* **2005**, *128*, 3098–3108.
6. Marik, P.E.; Linde-Zwirble, W.T.; Bittner, E.A.; Sahatjian, J.; Hansell, D. Fluid administration in severe sepsis and septic shock, patterns and outcomes: An analysis of a large national database. *Intensive Care Med.* **2017**, *43*, 625–632.
7. Famous, K.R.; Delucchi, K.; Ware, L.B.; Kangelaris, K.N.; Liu, K.D.; Thompson, B.T.; Calfee, C.S. ARDS subphenotypes respond differently to randomized fluid management strategy. *Am. J. Respir. Crit. Care Med.* **2017**, *195*, 331–338.
8. Dellinger, R.P.; Schorr, C.; Levy, M.M. A users’s guide to the 2016. Surviving Sepsis Guidelines. *Intensive Care Med.* **2017**, *43*, 299–303.
9. Jones, A.E.; Brown, M.D.; Trzeciak, S.; Shapiro, N.I.; Garrett, J.S.; Heffner, A.C.; Kline, J.A.; Emergency Medicine Shock Research Network Investigators. The effect of a quantitative resuscitation strategy on mortality in patients with sepsis: A meta-analysis. *Crit. Care Med.* **2008**, *36*, 2734–2739.
10. Waechter, J.; Kumar, A.; Lapinsky, S.E.; Marshall, J.; Dadek, P.; Arabi, Y.; Parillo, J.E.; Dellinger, R.P.; Garland, A.; Cooperative Antimicrobial Therapy of Septic Shock Date Base Research Group. Interaction between fluids and vasoactive agents on mortality in septic shock: A multicenter observational study. *Crit. Care Med.* **2014**, *42*, 2158–2168.
11. Mouncey, P.R.; Osborn, T.M.; Power, G.S.; Harrison, D.A.; Sadique, M.Z.; Grieve, R.D.; Jahan, R.; Sheila, S.E.; Bell, K.; Bion, J.F.; et al. Trial of early goal-directed resuscitation for septic shock. *N. Engl. J. Med.* **2015**, *372*, 1301–1311.
12. The ARISE Investigators and the ANZICS Clinical Trials Group.Goal-directed resuscitation for patients with early septic shock. *N. Engl. J. Med.* **2014**, *371*, 1496–1506.
13. The proCESS Investigators. A randomized trial of protocol-based care for early septic shock. *N. Engl. J. Med.* **2014**, *370*, 1683–1693.
14. The PRIM Investigators. Early goal-directed therapy for septic shock—A patient-level meta-analysis. *N. Engl. J. Med.* **2017**, *376*, 2223–2234.
15. Ferrer, R.; Artigas, A.; Suarez, D.; Palencia, E.; Levy, M.M.; Arenzana, A.; Pérez, X.L.; Sirvent, J.; Edusepsis Study Group. Effectiveness of treatments for severe sepsis: A prospective, multicenter, observational study. *Am. J. Respir. Crit. Care Med.* **2009**, *180*, 861–866.
16. Rhodes, A.; Evans, L.E.; Alhazzani, W.; Levy, M.M.; Antonelli, M.; Ferrer, R.; Kumar, A.; Sevransky, J.E.; Sprung, C.L.; Nunnally, M.E.; et al. Surviving Sepsis Campaign: International Guidelines for Management of Sepsis and Septic Shock: 2016. *Intensive Care Med.* **2017**, *45*, 486–552.
17. Cecconi, M.; De Backer, D.; Antonelli, M.; Beale, R.; Bakker, J.; Hofer, C.; Jaeschke, R.; Mebazaa, A.; Pinsky, M.R.; Teboul, J.L.; et al. Consensus on circulatory shock and hemodynamic monitoring. Task force of the European Society of Intensive Care Medecine. *Intensive Care Med.* **2014**, *40*, 1795–815.
18. Monnet, X.; Marik, P.; Teboul, J.L. Prediction of fluid responsiveness: An update. *Ann. Intensive Care* **2016**, *6*, 111.
19. Monnet, X.; Marik, P.; Teboul, J.L. Passive leg raising for predicting fluis responsiveness: A systematic review and meta-analysis. *Intensive Care Med.* **2016**, *42*, 1935–1947.
20. Jozwiak, M.; Teboul, J.L.; Monnet, X. Extravascular lung water in critical care: Recent advances and clinical applications. *Ann. Intensive Care* **2015**, *5*, 38.

## Vasopressors in Sepsis: When to Start, When to Stop?

HamzaouiOlfa^1^TeboulJean-Louis^2^,^3^1Hôpitaux universitaires Paris-Sud, Hôpital Antoine Béclère, service de réanimation polyvalente, 157, rue de la-porte-de-Trivaux, F-92141 Clamart, France; jean-louis.teboul@aphp.fr2Hôpitaux universitaires Paris-Sud, Hôpital de Bicêtre, service de réanimation médicale, 78, rue du Général Leclerc, F-94270 Le Kremlin-Bicêtre, France3INSERM UMR S_999, University Paris-Sud, 78, rue du Général Leclerc, F-94270 Le Kremlin-Bicêtre, France

**Introduction**

The prognosis of septic shock is tightly linked to the earliness of both antibiotic therapy and hemodynamic management, including administration of fluids and of vasopressors in order to target a mean arterial pressure (MAP) of at least 65 mmHg [1]. The early use of vasopressors—in practice norepinephrine—is one of the emerging concepts in hemodynamic management of septic shock.

**The Following Arguments Support the Early Use of Norepinephrine in Patients with Septic Shock**

- The duration and the degree of hypotension are associated with increased mortality.

The area of MAP under 65 mmHg during the first 48 h of resuscitation was shown to be the best predictor of 30-Day mortality in human septic shock [2]. This was one of the major arguments upon which the recommendations of the Surviving Sepsis Campaign (SSC) in terms of initial MAP target were based [1].

- Delayed initiation of norepinephrine is associated with increased mortality.

Fluid infusion alone cannot restore vascular tone, when it is severely depressed. In a multivariate logistic regression analysis, the time from the onset of resuscitation to norepinephrine initiation was shown to be an independent determinant of 28-Day mortality in septic shock [3].

- Early administration of norepinephrine increases cardiac output.

Norepinephrine increases the systemic venous return pressure gradient and the right atrial pressure [4]. When the patient is preload responsive, as is it the case in the early phase of sepsis, the increase in preload with norepinephrine increases cardiac output [5].

- Early administration of norepinephrine in severely hypotensive patients improves microcirculation.

In septic shock patients, early restoration of a MAP >65 mmHg with norepinephrine resulted in a significant increase in tissue oxygen saturation (StO_2_) measured at the thenar eminence using near-infra-red spectroscopy and in the StO_2_ recovery slope [6], which reflects the capacity of microvessels to be recruited in response to local hypoxia, and which is an important prognostic factor in septic shock patients [7].

- Early administration of norepinephrine prevents harmful fluid overload.

In a retrospective study [3], patients in whom norepinephrine was initiated within the first two hours of resuscitation received less fluids than patients with delayed initiated. This result also supports the early use of norepinephrine as it is well established that a positive fluid balance is an independent factor of mortality in septic shock [8].

A simple way to identify patients who early need norepinephrine, is to consider the diastolic arterial pressure (DAP), which mainly depends on vascular tone. An abnormally low DAP, especially in cases of tachycardia, is an indication to start norepinephrine urgently.

**What Is the Optimal Target?**

Current resuscitation guidelines in septic shock recommend to initially achieve and maintain MAP ≥65 mmHg, to avoid additional organ hypoperfusion [1]. A study investigating the effects on sublingual microcirculation of increasing doses of norepinephrine to target different MAP targets (65, 75 and 85 mmHg) in septic shock [9], showed a highly variable response confirming that individualized management is mandatory in septic shock. A multicenter randomized trial compared two MAP targets in patients with septic shock: 65–70 mmHg and 80–85 mmHg [10]. There was no differences between the two groups in 28-day mortality and in the rate of serious adverse events (SAEs) [10]. In the predefined stratum of patients with chronic hypertension, those with a MAP target of 80–85 mmHg exhibited a more pronounced improvement in kidney function and fewer requirements for renal replacement therapy [10]. This study thus suggests that in patients with chronic hypertension, targeting MAP between 80–85 mmHg is reasonable. A MAP target higher than 65 mmHg should also be considered in cases of high central venous pressure, which reflects a high outflow venous pressure within organs. Indeed, in such cases, it could be important to increase the MAP to maintain an adequate driving pressure for organ perfusion. Similarly, an increased intra-abdominal pressure can represent the outflow pressure of abdominal organs, necessitating then a higher than normal MAP to preserve their adequate perfusion unless reduction of the intra-abdominal pressure is feasible.

In all cases, achieving any specific target MAP does not guarantee successful correction of peripheral hypoperfusion since some dissociation between the macro- and the microcirculations is assumed to exist in sepsis [11]. It is likely that better markers of peripheral perfusion and/or microcirculation than MAP should be used in the future to individually titrate vasopressors.

**What to Do in Cases of Refractory Hypotension?**

Hypotension is often qualified as refractory to norepinephrine when rapidly increasing doses of norepinephrine fail to reach the MAP target, although no specific maximal dose of has been defined. The SSC recommends adding vasopressin to norepinephrine with the intent to reduce norepinephrine dosage or to raise MAP in case of refractory hypotension [1]. In septic shock, adding exogenous vasopressin was shown to increase MAP while reducing norepinephrine requirements in patients already receiving norepinephrine [12]. However, this strategy did not reduce mortality as compared with norepinephrine alone in this multi-center randomized trial [12]. The alternative option, which is to further increase the norepinephrine dose, is a matter of debate [13]. It is likely that in the future, identification of risks of development of SAEs using genotype markers will help clinicians to make the decision of either continuing norepinephrine or adding vasopressin or another vasopressor [14]. In the future, optimal vasopressor therapy could be a combination of multiple agents acting on different receptors with the intent to minimize doses of each agent and to increase safety [15]. Finally, the SSC suggests use of intravenous hydrocortisone if fluid and norepinephrine fail to restore hemodynamic stability, although no precise information about the most appropriate time of hydrocortisone initiation has been provided [1].

**Conclusions**

Norepinephrine should be initiated early during sepsis when a low DAP identifies a severely depressed vascular tone. A MAP target of at least 65 mmHg is recommended but in some conditions such as chronic hypertension a higher MAP target can be considered. Although addition of vasopressin to norepinephrine is recommended in cases of refractory hypotension, this is still a matter of debate.

**References**

1. Rhodes, A.; Evans, L.E.; Alhazzani, W.; Levy, M.M.; Antonelli, M.; Ferrer, R.; Kumar, A.; Sevransky, J.E.; Sprung, C.L.; Nunnally, M.E.; et al. Surviving Sepsis Campaign: International Guidelines for Management of Sepsis and Septic Shock: 2016. *Intensive Care Med.* **2017**, *45*, 486–552.
2. Varpula, M.; Tallgren, M.; Saukkonen, K.; Voipio-Pulkki, L.M.; Pettilä, V. Hemodynamic variables related to outcome in septic shock. *Intensive Care Med.* **2005**, *31*, 1066–1071.
3. Bai, X.; Yu, W.; Ji, W.; Lin, Z.; Tan, S.; Duan, K.; Dong, Y.; Xu, L.; Li, N. Early versus delayed administration of norepinephrine in patients with septic shock. *Crit. Care* **2014**, *18*, 532.
4. Persichini, R.; Silva, S.; Teboul, J.L.; Jozwiak, M.; Chemla, D.; Richard, C.; Monnet, X. Effects of norepinephrine on mean systemic pressure and venous return in human septic shock. *Crit. Care Med.* **2012**, *40*, 3146–3153.
5. Hamzaoui, O.; Georger, J.F.; Monnet, X.; Ksouri, H.; Maizel, J.; Richard, C.; Teboul, J.L. Early administration of norepinephrine increases cardiac preload and cardiac output in septic patients with life-threatening hypotension. *Crit. Care* **2010**, *14*, R142.
6. Georger, J.F.; Hamzaoui, O.; Chaari, A.; Maizel, J.; Richard, C.; Teboul, J.L. Restoring arterial pressure with norepinephrine improves muscle tissue oxygenation assessed by near-infrared spectroscopy in severely hypotensive septic patients. *Intensive Care Med.* **2010**, *36*, 1882–1889.
7. Creteur, J.; Carollo, T.; Soldati, G.; Buchele, G.; De Backer, D.; Vincent, J.L. The prognostic value of muscle StO_2_ in septic patients. *Intensive Care Med.* **2007**, *33*, 1549–1556.
8. Acheampong, A.; Vincent, J.L. A positive fluid balance is an independent prognostic factor in patients with sepsis. *Crit. Care* **2015**, *19*, 251.
9. Dubin, A.; Pozo, M.O.; Casabella, C.A.; Pálizas, F., Jr.; Murias, G.; Moseinco, M.C.; Kanoore Edul, V.S.; Pálizas, F.; Estenssoro, E.; Ince, C. Increasing arterial blood pressure with norepinephrine does not improve microcirculatory blood flow: A prospective study. *Crit. Care* **2009**, *13*, R92.
10. Asfar, P.; Meziani, F.; Hamel, J.F.; Grelon, F.; Megarbane, B.; Anguel, N.; Mira, J.P.; Dequin, P.F.; Gergaud, S.; Weiss, N.; et al. High versus low blood-pressure target in patients with septic shock. *N. Engl. J. Med.* **2014**, *370*, 1583–1593.
11. Hernández, G.; Teboul, J.L. Is the macrocirculation really dissociated from the microcirculation in septic shock? *Intensive Care Med.* **2016**, *42*, 1621–1624.
12. Russell, J.A.; Walley, K.R.; Singer, J.; Gordon, A.C.; Hébert, P.C.; Cooper, D.J.; Holmes, C.L.; Mehta, S.; Granton, J.T.; Storms, M.M. VASST Investigators. Vasopressin versus norepinephrine infusion in patients with septic shock. *N. Engl. J. Med.* **2008**, *358*, 877–887.
13. Auchet, T.; Regnier, M.A.; Girerd, N.; Levy, B. Outcome of patients with septic shock and high-dose vasopressor therapy. *Ann. Intensive Care* **2017**, *7*, 43.
14. Hamzaoui, O.; Scheeren, T.W.L.; Teboul, J.L. Norepinephrine in septic shock: When and how much? *Curr. Opin. Crit. Care* **2017**, *23*, 342–347.
15. Teboul, J.L.; Duranteau, J.; Russell, J.A. Intensive care medicine in 2050: Vasopressors in sepsis. *Intensive Care Med.* **2017**, doi:10.1007/s00134-017-4909-7.

##                                        

 

SESSION III. “TRANSLATIONAL MEDICINE: THE IMMUNE RESPONSE IN SEPSIS”

 

## Pathogen-Host Interaction in Sepsis: The Role of the Endothelium

KerriganSteveIrish Centre for Vascular Biology, Sepsis Research Group, Royal College of Surgeons in Ireland, Dublin, Ireland

**Abstract:** The vascular endothelium is a major target of sepsis-induced events and endothelial cell damage accounts for much of the pathology of septic shock [1]. Endothelial cells are a highly metabolically active monocell layer that is constantly sensing alterations in the local extracellular environment such as transient bacteremia, minor trauma or other common daily stresses [2]. A common feature in patients with sepsis is progressive subcutaneous and body cavity oedema typically caused by permeabilization of the vascular endothelial cell monolayer. In addition, hyper and disseminated intravascular coagulation is also observed in sepsis patients leading to occlusion of microvessels by circulating thrombi [2]. The coordinated progression of increased vascular permeability and together with the dysregulated hypercoagulation leads to multiple organ failure in patients with sepsis. Vascular permeability leading to accumulation of parenchymal and interstitial fluid can potentially impair organ function by increasing the distance required for the diffusion of oxygen and by compromising micro vascular perfusion because of increased interstitial pressure [3]. Indeed reduction in oedema is a well-recognised feature of recovery from sepsis. Circulating thrombi as a result of platelet-bacterial interactions leading to occlusion of a blood vessel has been well established. As a first line of defence, endothelial cells recognise invading bacteria through specific proteinprotein interactions. These interactions result in the release of many endotheliumderived mediators critical for the recruitment of leukocytes and inflammation, responses consistent with the clinical signs of sepsis. The inflammatory response plays a key role in the sepsis phenotype and an excessive or sustained inflammatory response contributes to the tissue damage and death [4]. Currently, there are no approved drugs on the market to control the underlying pathophysiology that triggers the uncontrolled host response to sepsis. Therefore the current principles of sepsis management remains to optimise organ perfusion with intravenous fluids (vasopressors if required), support dysfunctional organ systems (e.g., ventilation) and mitigate the immediate threat of uncontrolled infection with antibiotic therapy and source control. Despite significant advances in our understanding of the pathophysiology of the host response to sepsis, targeted therapies to disrupt the aberrant host-pathogen interaction are lacking. This paper will discuss the value of specifically targeting the vascular endothelium and will discuss a potential novel drug target that could be used to treat the vascular injury in sepsis. A major limitation in our current understanding of how bacteria interact with endothelial cells is that almost all previous in vitro studies on endothelial cells have been carried out using cells grown under static conditions in a 2D environment. Data obtained using statically grown endothelial cells may not be physiologically relevant as in the circulation they are subjected to a fluid dynamic environment which determines cellular morphology and function. To address this we developed a clinically relevant real time epifluorescent shear based 2D model of sepsis where monolayers of Human Endothelial Cells were exposed to shear stress at levels experienced in the human circulation [5]. To further validate our findings we fabricated a 3D microvessel containing human endothelial cells exposed to physiological shear rates experienced in the vasculature. Using these models we demonstrated that endothelial cells grown under static conditions do not stick together to form a tight barrier but rather exhibit a random pattern of growth not characteristic of a functional blood vessel found in the human circulation. In contrast, human endothelial cells sheared at physiological rates experienced in the vasculature, stick together and form cell-cell contacts characteristic of a functional tight barrier found in blood vessels in humans. Most importantly we found that both *S. aureus* and *E. coli* bind to sheared human endothelial cells much better than to statically grown cells. These results suggest that shearing the endothelial cells is critical in order to investigate the molecular mechanisms that lead to bacterial binding. Using these models we identified the molecular mechanisms that both *S. aureus* and *E. coli* use to trigger endothelial cell dysfunction such as loss of barrier function and apoptosis which leads to vascular leak [5]. Strikingly, our studies demonstrate that under shear based conditions both *S. aureus* and *E. coli* bind to the same major endothelial cell receptor, suggestive of a common mechanism through which bacteria induce endothelial dysfunction. Blocking the endothelial cell receptor either before the infection or post infection prevents the injurious effect of bacteria on the human endothelial cells [6]. Finally to translate our findings and investigate if blocking bacterial interaction with the endothelium might serve as a potential novel approach to treating sepsis early we used mouse and rat models of sepsis [5]. Consistent with our results above blocking the endothelial cell receptor in vivo prevents bacterial attachment and therefore prevents all downstream injurious effects, such as loss of barrier integrity, on the endothelium that is associated with the infection. The implications of these findings are important as preventing bacteria from binding to the endothelium, either pre-emptively or therapeutically, could slow or reduce the progression of sepsis to multi-organ failure and death.

**References**

1. Kerrigan, S.W.; McDonnell, C. Dysregulation of the endothelium during *S. aureus* infection. *Biochem. Soc. Trans.* **2015**, *43*, 715–719.
2. Lemichez, E.; Lecuit, M.; Nassif, X.; Bourdoulous, S. Breaking the wall: Targeting of the endothelium by pathogenic bacteria. *Nat. Rev. Microbiol.* **2010**, *8*, 93–104.
3. Angus, D.C.; van der Poll, T. Severe sepsis and septic shock. *N. Engl. J. Med.* **2013**, *369*, 840–851.
4. Deutschman, C.S.; Tracey, K.J. Sepsis: Current dogma and new perspectives. *Immunity* **2014**, *40*, 464–476.
5. McDonnell, C.; Garciarena, C.; Watkin, R.; McHale, T.; McLoughlin, A.; Claes, J.; Verhamme, P.; Cummins, P.; Kerrigan, S.W. Staphylococcus aureus-mediated endothelial cell dysregulation: Implications for the treatment of sepsis. *J. Thromb. Haemost.*
6. Garciarena, C.; McHale, T.; Martin-Loeches, I.; Kerrigan, S.W. Pre-emptive and therapeutic value of blocking bacterial attachment to the endothelial alphaVbeta3 integrin with cilengitide in sepsis. *Crit. Care* **2017**, *21*, 246.

## The Host Response in Sepsis Patients Developing ICU Acquired Secondary Infections

PèneFrédéricMedical ICU, Cochin hospital, Assistance Publique—Hôpitaux de Paris & University Paris Descartes, Paris, France

**Abstract:** Sepsis represents the life-threatening inflammatory response to infection and is the leading cause of death in non-coronary intensive care units (ICU). Thanks to early infection control and advanced life support, a majority of patients now survive the first days of multiple organ failure, but then become exposed to ICU-acquired bacterial and fungal infections, as well as viral reactivation of *Herpes simplex virus* and cytomegalovirus [1–4]. This wide range of infections suggests a complex immunosuppressive response, and septic patients indeed exhibit multiple quantitative and functional defects affecting both innate and adaptive components of immunity [5] [6]. Most immune defects have been linked to relevant endpoints in critically ill patients, such as mortality and the development of ICU-acquired infections. The deactivation of monocytes, characterized by a decreased expression of the antigen presentation apparel and a blunted cytokine production in response to ex vivo challenge with LPS, is a hallmark of post-aggressive immunosuppression. A low expression of HLA-DR on monocytes sustains an increased susceptibility to ICU-acquired infections and is currently considered as the most potent biomarker in the field [7]. More recently, whole-genome transcriptomic assays have added a new layer of complexity in the comprehension of immune regulation in septic critically ill patients, and convey the emerging concept of immune reprogramming [1,8]. However, a reliable assessment of the impact of sepsis-induced immunosuppression on the susceptibility and response to ICU-acquired infections in critically ill patients is limited by multiple confounding factors. In addition, most immune investigations in humans are restricted to circulating leukocytes, with limited access to organ immune cells. Relevant double-hit experimental animal models have been developed in order to overcome this caveat. The high susceptibility of post-septic animals to secondary infectious insults such as *Pseudomonas aeruginosa* pneumonia provides a definite proof-of-concept of sepsis-induced immunosuppression [9]. Furthermore, these models allow an accurate assessment of defective lung defense in post-septic animals and thereby represent irreplaceable tools to address the regulatory cellular and molecular mechanisms [10]. Beyond the classical sepsis-related overwhelming inflammatory response, clinical and experimental data have allowed defining the paradigm of sepsis-induced immunosuppression that conveys the clinical course of sepsis in the current era. The development of immune biomarkers may help at stratifying the risk of infections in the critically ill and identifying the patients likely to benefit from immune-enhancing targeted therapies under current evaluation in this indication.

**References**

1. Van Vught, L.A.; Klein Klouwenberg, P.M.; Spitoni, C.; Scicluna, B.P.; Wiewel, M.A.; Horn, J.; Schultz, M.J.; Nurnberg, P.; Bonten, M.J.; Cremer, O.L.; et al. Incidence, Risk Factors, and Attributable Mortality of Secondary Infections in the Intensive Care Unit After Admission for Sepsis. *JAMA* **2016**, *315*, 1469–1479.
2. Daviaud, F.; Grimaldi, D.; Dechartres, A.; Charpentier, J.; Geri, G.; Marin, N.; Chiche, J.D.; Cariou, A.; Mira, J.P.; Pène, F. Timing and causes of death in septic shock. *Ann. Intensive Care* **2015**, *5*, 16.
3. Jamme, M.; Daviaud, F.; Charpentier, J.; Marin, N.; Thy, M.; Hourmant, Y.; Mira, J.P.; Pene, F. Time Course of Septic Shock in Immunocompromised and Nonimmunocompromised Patients. *Crit. Care Med.* **2017**, *45*, 2031–2039.
4. Walton, A.H.; Muenzer, J.T.; Rasche, D.; Boomer, J.S.; Sato, B.; Brownstein, B.H.; Pachot, A.; Brooks, T.L.; Deych, E.; Shannon, W.D.; et al. Reactivation of multiple viruses in patients with sepsis. *PLoS ONE* **2014**, *9*, e98819.
5. Boomer, J.S.; To, K.; Chang, K.C.; Takasu, O.; Osborne, D.F.; Walton, A.H.; Bricker, T.L.; Jarman, S.D., II; Kreisel, D.; Krupnick, A.S.; et al. Immunosuppression in patients who die of sepsis and multiple organ failure. *JAMA* **2011**, *306*, 2594–2605.
6. Hotchkiss, R.S.; Monneret, G.; Payen, D. Sepsis-induced immunosuppression: From cellular dysfunctions to immunotherapy. *Nat. Rev. Immunol.* **2013**, *13*, 862–874.
7. Landelle, C.; Lepape, A.; Voirin, N.; Tognet, E.; Venet, F.; Bohe, J.; Vanhems, P.; Monneret, G. Low monocyte human leukocyte antigen-DR is independently associated with nosocomial infections after septic shock. *Intensive Care Med.* **2010**, *36*, 1859–1866.
8. Shalova, I.N.; Lim, J.Y.; Chittezhath, M.; Zinkernagel, A.S.; Beasley, F.; Hernandez-Jimenez, E.; Toledano, V.; Cubillos-Zapata, C.; Rapisarda, A.; Chen, J.; et al. Human Monocytes Undergo Functional Re-programming during Sepsis Mediated by Hypoxia-Inducible Factor-1alpha. *Immunity* **2015**, *42*, 484–498.
9. Pène, F.; Zuber, B.; Courtine, E.; Rousseau, C.; Ouaaz, F.; Toubiana, J.; Tazi, A.; Mira, J.P.; Chiche, J.D. Dendritic cells modulate lung response to *Pseudomonas aeruginosa* in a murine model of sepsis-induced immune dysfunction. *J. Immunol.* **2008**, *181*, 8513–8520.
10. Roquilly, A.; McWilliam, H.E.G.; Jacqueline, C.; Tian, Z.; Cinotti, R.; Rimbert, M.; Wakim, L.; Caminschi, I.; Lahoud, M.H.; Belz, G.T.; et al. Local Modulation of Antigen-Presenting Cell Development after Resolution of Pneumonia Induces Long-Term Susceptibility to Secondary Infections. *Immunity* **2017**, *47*, 135–147.e135.

## Immunomonitoring in Patients with Sepsis

RoatErikaBiagioniEmanuelaTosiMartinaGirardisMassimoIntensive Care Unit, Modena University Hospital, L.go del Pozzo 71, 41100 Modena, Italy

**Abstract:** The new definition of sepsis as a life-threatening organ dysfunction caused by a dysregulated host response to infection clearly focused on the pivotal role of the immune response in the pathobiology of this condition [1]. Despite an important step forward, the new definitions and classification did not solve the issue of high heterogeneity in sepsis population. For instance, referring to host response, septic patients may display different inflammatory and immune phenotypes as a function of the load and virulence of micro-organisms, site of infection, individual differences in immune system related to genetic heterogeneity and/or preexisting disease, time from sepsis occurrence and treatments provided. For instance, in some cases a disproportionate inflammatory-immune hyperactivity may rapidly lead to organ dysfunction and early death by the so called “cytokine storm”. On the other hand, in some cases we can observe a state of immune-paralysis characterized by a blunted response to pathogens due to cell anergy and apoptosis responsible for secondary infections, mostly by opportunistic agents, and viral reactivations leading to long term deaths [2,3]. The two described pathophysiological phenotypes may also explain the biphasic curve observed in the timing of death of septic patients [2,3] with early deaths—i.e., within 72 h—related to an excessive hyperinflammatory response and late deaths—i.e., after two-three weeks—attributed to exhaustion of immune response [4]. Indeed, these phenotypes are just an oversimplification because numerous studies demonstrated the coexistence of both pro and anti-inflammatory responses during sepsis with different entities and time profiles [5]. In the last years, the population ageing with multiple co-existing diseases and immune-senescence [6], the extended treatment of neoplastic diseases by aggressive surgery and chemotherapy, and the increased use immunosuppressive drugs led to growing number of patients showing an immune-paralysis pattern at the beginning of sepsis. In this condition, sepsis management is often complicated because of recurrent and/or breakthrough infections requiring prolonged use of antibiotics with selection of multi drug resistant pathogens [7,8]. Identification of immune-dysfunction and, then, use of specific adjunctive therapies aimed to support the immune-system may be the keys for improving the survival rate of this specific population. In sepsis, numerous mechanisms may cause dysfunction of the immune system which can last up to six months before recovery. An altered expression of costimulatory receptors on antigen presenting cells and dysfunction of the monocyte-macrophage system with a reduction in HLA-DR expression [9–12] have been frequently observed in septic patients. An increase in apoptosis has been also described for both innate and adaptive immune cells associated with a suppression of specific immunity and the increase of regulatory T cells which exert an immunosuppressive function [13,14]. Another important mechanism of immune-paralysis is the alteration in cytokine synthesis with an imbalanced production of anti-inflammatory molecules disturbing several functions of leukocytes subpopulations. Several biomarkers have been identified both in animal models and patients to evaluate presence, magnitude and mechanisms of immune-dysfunction during sepsis. Unfortunately, many of these biomarkers are unusable in clinical practice, especially those measuring lymphocytes function which need long turn-around time, high expertise and significant budget [15,16]. Nevertheless, today some easy to measure biomarker is available to define the immune phenotype of the patient at the bed side.

- HLA-DR expression on monocytes is broadly used to test innate immunity and reduced levels of HLA-DR are predictive of adverse outcome and septic complication after trauma, surgery, pancreatitis, burn and septic shock [17,18].
- Lymphocytes count is related to adaptive immune-response. Persistent lymphopenia during sepsis is associated to high risk of secondary infections and mortality [19].
- Neutrophils to lymphocyte ratio (NLR). In patients with septic shock, low NRL values at admission are associated with a higher risk of early death whereas high NRL values during sepsis course are related to late death, probably because of decrease or not recovery in lymphocyte count [20].
- Immunoglobulins (Ig) plasma concentration levels are predictive for outcome both at sepsis onset and during sepsis course and, therefore, may be used for assessing immune-status during sepsis [21].
- Viral reactivations and infections by opportunistic micro-organisms. IN septic patients CMV, EBV and HSV1 reactivation is frequently (up to 60% of the patients) and it is associated with an higher risk of secondary infections and late mortality [22,23]. Similarly, infections by opportunistic bacteria and fungi are frequently favored by impairment of the innate and adaptive immunity [24].

Although generic and non-specific for single mechanism and function, the bio-markers proposed patients seems to be feasible and useful for assessing and monitoring the immune phenotype of septic patients. The central role and the frequent dysfunction of the immune response in sepsis led to develop and test different immunomodulatory strategies in the last decades. Colony stimulating factors, cytokines (e.g., INF-γ, IL-3, IL-7, IL-15), immunoglobulins with their pleiotropic effects on different compartments of the immune system and blood purification have been proposed as adjunctive immune therapies [25,26]. Promising results have been also recently obtained in animal models by use of monoclonal antibodies directed to PD-1/PD-L1 pathway and to CTLA-4 [27,28] and specific clinical trials are underway. Unfortunately, up to now we have not clear data on which is the patient who could benefit from the use of these therapies that are frequently used as compassionate and last ditch. Nevertheless, in the near future the ongoing clinical trials will provide data for the appropriate use of immunomodulatory therapies in septic population. In the mean time, we believe that immune system should be considered a pivotal ‘organ’ in sepsis and, then, its dysfunction should be assessed regularly in patients with sepsis and septic shock by easy parameters (e.g., lymphocytes count, immunoglobulins plasma concentration) as reccomended for other organs by SOFA score.

**References**

1. Singer, M.; Deutschman, C.S.; Seymour, C.W.; Shankar-Hari, M.; Annane, D.; Bauer, M.; Bellomo, R.; Bernard, G.R.; Chiche, J.D.; Coopersmith, C.M.; et al. The Third International Consensus Definitions for Sepsis and Septic Shock (Sepsis-3). *JAMA* **2016**, *315*, 801–810.
2. Otto, G.P.; Sossdorf, M.; Claus, R.A.; Rödel, J.; Menge, K.; Reinhart, K.; Bauer, M.; Riedemann, N.C. The late phase of sepsis is characterized by an increased microbiological burden and death rate. *Crit. Care* **2011**, *15*, R183.
3. Iskander, K.N.; Osuchowski, M.F.; Stearns-Kurosawa, D.J.; Kurosawa, S.; Stepien, D.; Valentine, C.; Remick, D.G. Sepsis: Multiple abnormalities, heterogeneous responses, and evolving understanding. *Physiol. Rev.* **2013**, *93*, 1247–1288.
4. Daviaud, F.; Grimaldi, D.; Dechartres, A.; Charpentier, J.; Geri, G.; Marin, N.; Chiche, J.D.; Cariou, A.; Mira, J.P.; Pène, F. Timing and causes of death in septic shock. *Ann. Intensive Care* **2015**, *5*, 16.
5. Tamayo, E.; Fernandez, A.; Almansa, R.; Carrasco, E.; Heredia, M.; Lajo, C.; Goncalves, L.; Gomez-Herreras, J.L.; de Lejarazu, R.O.; Bermejo-Martin, J.F. Pro- and anti-inflammatory responses are regulated simultaneously from the first moments of septic shock. *Eur. Cytokine Netw.* **2011**, *22*, 82–87.
6. Reber, A.J.; Chirkova, T.; Kim, J.H.; Cao, W.; Biber, R.; Shay, D.K.; Sambhara, S. Immunosenescence and challenges of vaccination against influenza in the aging population. *Aging Dis.* **2012**, *3*, 68–90.
7. Schefold, J.C.; Hasper, D.; Volk, H.D.; Reinke, P. Sepsis: Time has come to focus on the later stages. *Med. Hypotheses* **2008**, *71*, 203–208.
8. Limaye, A.P.; Kirby, K.A.; Rubenfeld, G.D.; Leisenring, W.M.; Bulger, E.M.; Neff, M.J.; Gibran, N.S.; Huang, M.L.; Santo, Hayes T.K.; Corey, L.; et al. Cytomegalovirus reactivation in critically ill immunocompetent patients. *JAMA* **2008**, *300*, 413–422.
9. Boomer, J.S.; To, K.; Chang, K.C.; Takasu, O.; Osborne, D.F.; Walton, A.H.; Bricker, T.L.; Jarman, S.D., II; Kreisel, D.; Krupnick, A.S.; et al. Immunosuppression in patients who die of sepsis and multiple organ failure. *JAMA* **2011**, *306*, 2594–605.
10. Landelle, C.; Lepape, A.; Voirin, N.; Tognet, E.; Venet, F.; Bohe, J.; Vanhems, P.; Monneret, G. Low monocyte human leukocyte antigen-DR is independently associated with nosocomial infections after septic shock. *Intensive Care Med.* **2010**, *36*, 1859–1866.
11. Monneret, G.; Lepape, A.; Voirin, N.; Bohé, J.; Venet, F.; Debard, A.L.; Thizv, H.; Bienvenu, J.; Gueyffier, F.; Vanhems, P. Persisting low monocyte human leukocyte antigen-DR expression predicts mortality in septic shock. *Intensive Care Med.* **2006**, *32*, 1175–1183.
12. Suarez-Santamaria, M.; Santolaria, F.; Perez-Ramirez, A.; Alemán-Valls, M.R.; Martínez-Riera, A.; González-Reimers, E.; de la Vega, M.J.; Milena, A. Prognostic value of inflammatory markers (notably cytokines and procalcitonin), nutritional assessment, and organ function in patients with sepsis. *Eur. Cytokine Netw.* **2010**, *21*, 19–26.
13. Hotchkiss, R.S.; Osmon, S.B.; Chang, K.C.; Wagner, T.H.; Coopersmith, C.M.; Karl, I.E. Accelerated lymphocyte death in sepsis occurs by both the death receptor and mitochondrial pathways. *J. Immunol.* **2005**, *174*, 5110–5118.
14. Kessel, A.; Bamberger, E.; Masalha, M.; Toubi, E. The role of T regulatory cells in human sepsis. *J. Autoimmun.* **2009**, *32*, 211–215.
15. Boomer, J.S.; Shuherk-Shaffer, J.; Hotchkiss, R.S.; Green, J.M. A prospective analysis of lymphocyte phenotype and function over the course of acute sepsis. *Crit. Care.* **2012**, *16*, R112.
16. Rol, M.L.; Venet, F.; Rimmele, T.; Moucadel, V.; Cortez, P.; Quemeneur, L.; Gardiner, D.; Griffiths, A.; Pachot, A.; Textoris, J.; et al. The REAnimation Low Immune Status Markers (REALISM) project: A protocol for broad characterisation and follow-up of injury-induced immunosuppression in intensive care unit (ICU) critically ill patients. *BMJ Open* **2017**, *7*, e015734.
17. Cazalis, M.A.; Friggeri, A.; Cavé, L.; Demaret, J.; Barbalat, V.; Cerrato, E.; Lepape, A.; Pachot, A.; Monneret, G.; Venet, F. Decreased HLA-DR antigen-associated invariant chain (CD74) mRNA expression predicts mortality after septic shock. *Crit. Care* **2013**, *17*, R287.
18. Cheron, A.; Floccard, B.; Allaouchiche, B.; Guignant, C.; Poitevin, F.; Malcus, C.; Crozon, J.; Faure, A.; Guillaume, C.; Marcotte, M.; et al. Lack of recovery in monocyte human leukocyte antigen-DR expression is independently associated with the development of sepsis after major trauma. *Crit. Care* **2010**, *14*, R208.
19. Drewry, A.M.; Samra, N.; Skrupky, L.P.; Fuller, B.M.; Compton, S.M.; Hotchkiss, R.S. Persistent lymphopenia after diagnosis of sepsis predicts mortality. *Shock* **2014**, *42*, 383–391.
20. Riché, F.; Gayat, E.; Barthélémy, R.; Le Dorze, M.; Máteo, J.; Payen, D. Reversal of neutrophil-to-lymphocyte count ratio in early versus late death from septic shock. *Crit. Care.* **2015**, *19*, 439.
21. Bermejo-Martín, J.; Rodriguez-Fernandez, A.; Herrán-Monge, R.; Andaluz-Ojeda, D.; Muriel-Bombín, A.; Merino, P.; García-García, M.M.; Citores, R.; Gandía, F.; Almansa, R.; et al. Immunoglobulins IgG1, IgM and IgA: A synergistic team influencing survival in sepsis. *J. Intern. Med.* **2014**, *276*, 404–412.
22. Heininger, A.; Haeberle, H.; Fischer, I.; Beck, R.; Riessen, R.; Rohde, F.; Meisner, C.; Jahn, G.; Koenigsrainer, A.; Unerti, K.; et al. Cytomegalovirus reactivation and associated outcome of critically ill patients with severe sepsis. *Crit. Care* **2011**, *15*, R77.
23. Textoris, J.; Mallet, F. Immunosuppression and herpes viral reactivation in intensive care unit patients: One size does not fit all. *Crit. Care* **2017**, *21*, 230.
24. Patil, N.K.; Guo, Y.; Luan, L. Targeting Immune Cell Checkpoints during Sepsis. *Int. J. Mol. Sci.* **2017**, *18*, 2413.
25. Hamers, L.; Kox, M.; Pickkers, P. Sepsis-induced immunoparalysis: Mechanisms, markers, and treatment options. *Minerva Anestesiol.* **2015**, *81*, 426–439.
26. Naeem, K.; Patil, N.K.; Bohannon, J.K. Immunotherapy: A promising approach to reverse sepsis-induced immunosuppression. *Pharmacol. Res.* **2016**, *111*, 688–702.
27. Shao, R.; Fang, Y.; Yu, H.; Zhao, L.; Jiang, Z.; Li, C.S. Monocyte programmed death ligand-1 expression after 3–4 days of sepsis is associated with risk stratification and mortality in septic patients: A prospective cohort study. *Crit. Care* **2016**, *20*,124.
28. Inoue, S.; Bo, L.; Bian, J.; Unsinger, J.; Chang, K.; Hotchkiss, R.S. Dose-dependent effect of anti-CTLA-4 on survival in sepsis. *Shock* **2011**, *36*, 38–44.

## Local Immunity against Infection in the Respiratory Tract

MillsKingston H.G.WilkMieszko M.MisiakAlicjaBorknerLisaCurhamLucyImmune Regulation Research Group, School of Biochemistry and Immunology, Trinity Biomedical Sciences Institute, Trinity College Dublin, Dublin, Ireland

**Abstract:** The upper respiratory tract and lungs, like other mucosal surfaces, are constantly exposed to infectious agents. Indeed respiratory tract infections are a major cause of morbidity and mortality in humans, and accounted for 3.2 million deaths (7% of all deaths) globally in 2015. Furthermore, lower respiratory tract infections are still the leading cause of death in low income countries. Even vaccine-preventable respiratory infectious diseases like whooping cough (pertussis) still account for around 200,000 deaths annually, mostly in young infants. Studies in animal models of pertussis infection and in children with whooping cough have provided evidence that the innate and adaptive immune systems play critical roles in control of infection of the respiratory tract with *Bordetella pertussis* [1]. The innate immune system, including tissue resident macrophages and lung infiltrating neutrophils, natural killer (NK) cells, γδ T cells and anti-microbial peptides provide the first line of defense and in most individuals help to control the infection before adaptive immune responses promote pathogen clearance. However, very young infants can develop severe pertussis-related disease, with leukocytosis, pulmonary hypertension and pneumonia, which despite macrolide antibiotic treatment, can result in death. Studies in mouse models have shown that infection with *B. pertussis* is associated with rapid (2 h) production of inflammatory cytokines and chemokines in the lungs, including IL-1β, TNF, MIP-1α, and MIP-2α, typically produced by innate immune cells including macrophages and dendritic cells (DCs) in response to LPS and other pathogen-associated molecular patterns (PAMPs) produced by the bacteria [2]. Furthermore, γδ T cells, a small population of T cells that are prevalent at mucosal surfaces, are recruited to the lungs within a few hours of infection with *B. pertussis.* These ‘innate’ γδ T cells secrete early IL-17 without TCR activation. A second wave of γδ T cells that are antigen-specific are expanded in the lung 7–14 days after challenge, and these cells, together with Th17 cells produce further IL-17, which helps to recruit neutrophils [3]. IFN-γ-secreting NK cells are also recruited to the lungs during infection with *B. pertussis* and play a role in macrophage activation and in addition direct the induction of antigen-specific Th1 cells [4]. Complete clearance of the bacteria and prevention of re-infection is dependent on antigen-specific CD4 Th1 and Th17 cells. Furthermore, *B. pertussis*-specific Th1 cells have been found in the circulation of infants with or convalescing from whooping cough [5]. The severity and persistence of infection with *B. pertussis* is in part a reflection of the immune subversion strategies evolved by the pathogen. *B. pertussis* secretes a range of toxins and other virulence factors that either directly suppress the function of macrophages and neutrophils or promote the induction of regulatory macrophages/DCs and regulatory T (Treg) cells that suppress protective Th1 and Th17 responses [6]. Interestingly these Treg cells also play a protective role during acute infection by limiting infection-induced immunopathology. LPS activation of TLR4, together with other bacterial virulence factors, including filamentous hemagglutinin and adenylate cyclase toxin promote IL-10 production from macrophages and DCs, which controls lung pathology during *B. pertussis* infection [7]. Therefore optimum protective immunity against lung infection is dependent on a balance between effector and regulatory responses, allowing clearance of infection while limiting collateral damage through infection-induced immunopathology. Prevention of re-infection with *B. pertussis* and optimum vaccine-induced adaptive immunity is mediated by induction of memory T and B cells. Recent evidence has suggested that tissue-resident memory T (T_RM_) cells, memory T cells that reside in tissues without recirculating provide a first line of defence against reinfection. We have recently demonstrated that CD69^+^CD103^+^ CD4 T_RM_ cells accumulate in the lungs of mice during infection with *B. pertussis* and significantly expand through local proliferation following reinfection [8]. These *B. pertussis*-specific CD4 T_RM_ cells secrete IL-17 or IL-17 and IFN-γ and mediate protection against re-infection with *B. pertussis.* We also demonstrated that pathogen-specific and tissue-resident γδ T cells expand in the lungs following reinfection and play a role in adaptive immunity to *B. pertussis* [3]. Although these T_RM_ cells are antigen-specific, evidence is emerging that they may be activated by cytokines without TCR activation, suggesting that they behave like innate cells and that the maintenance of memory may be possible in the absence of specific antigen re-stimulation. Collectively the findings suggests co-operation and convergence of the innate and adaptive immune responses in the control of infection at mucosal surfaces such as the respiratory tract.

**References**

1. Higgs, R.; Higgins, S.C.; Ross, P.J.; Mills, K.H. Immunity to the respiratory pathogen *Bordetella pertussis. Mucosal Immunol.* **2012**, *5*, 485–500.
2. Bernard, N.J.; Finlay, C.M.; Tannahill, G.M.; Cassidy, J.P.; O’Neill, L.A.; Mills, K.H. A critical role for the TLR signaling adapter Mal in alveolar macrophage-mediated protection against *Bordetella pertussis. Mucosal Immunol.* **2015**, *8*, 982–992.
3. Misiak, A.; Wilk, M.M.; Raverdeau, M.; Mills, K.H. IL-17-Producing Innate and Pathogen-Specific Tissue Resident Memory gammadelta T Cells Expand in the Lungs of *Bordetella pertussis*-Infected Mice. *J. Immunol.* **2017**, *198*, 363–374.
4. Byrne, P.; McGuirk, P.; Todryk, S.; Mills, K.H. Depletion of NK cells results in disseminating lethal infection with *Bordetella pertussis* associated with a reduction of antigen-specific Th1 and enhancement of Th2, but not Tr1 cells. *Eur. J. Immunol.* **2004**, *34*, 2579–2588.
5. Ryan, M.; Murphy, G.; Gothefors, L.; Nilsson, L.; Storsaeter, J.; Mills, K.H. *Bordetella pertussis* respiratory infection in children is associated with preferential activation of type 1 T helper cells. *J. Infect. Dis.* **1997**, *175*, 1246–1250.
6. McGuirk, P.; McCann, C.; Mills, K.H. Pathogen-specific T regulatory 1 cells induced in the respiratory tract by a bacterial molecule that stimulates interleukin 10 production by dendritic cells: A novel strategy for evasion of protective T helper type 1 responses by *Bordetella pertussis. J. Exp. Med.* **2002**, *195*, 221–231.
7. Higgins, S.C.; Lavelle, E.C.; McCann, C.; Keogh, B.; McNeela, E.; Byrne, P.; O’Gorman, B.; Jarnicki, A.; McGuirk, P.; Mills, K.H. Toll-like receptor 4-mediated innate IL-10 activates antigen-specific regulatory T cells and confers resistance to *Bordetella pertussis* by inhibiting inflammatory pathology. *J. Immunol.* **2003**, *171*, 3119–3127.
8. Wilk, M.M.; Misiak, A.; McManus, R.M.; Allen, A.C.; Lynch, M.A.; Mills, K.H.G. Lung CD4 Tissue-Resident Memory T Cells Mediate Adaptive Immunity Induced by Previous Infection of Mice with *Bordetella pertussis. J. Immunol.* **2017**, *199*, 233–243.

##                                        

 

SESSION IV. “BRINGING PRECISION MEDICINE TO SEPSIS: EMERGING METHODS TO PERSONALIZE SEPSIS CARE”

 

## Impact of Source Infection in Sepsis

MartínezM.L.Intensive Care Unit, Hospital Universitario General de Catalunya, Sant Cugat del Vallés, Barcelona, Spain

**Abstract:** Sepsis is a complex systemic inflammatory reaction to an infection. Infection initiates cytokine release, leading to a global inflammatory cascade [1]. Although current knowledge suggests that the primary driver of mortality in septic shock is the systemic inflammatory response, the trigger of this response is uncontrolled infection arising from a specific anatomic source. Leligdowicz et al. [2] concluded in a large study of patients with septic shock that the anatomic source of infection is associated with hospital mortality in crude analysis and after adjustment for non-modifiable and modifiable factors. According to Laffey et al. [3], non-modifiable factors are those associated with demographic (age, sex) and risk factors (CHARLSON, community vs. hospital acquired infection, organism type) factors. While modifiable factors include illness severity (APACHE-II, SOFA), management (appropriate antibiotic treatment, source control) and ICU organizational factors (number of beds). Considering this, it is reasonable to think that the anatomical source of infection will determine certain clinical characteristics in patients that may be involved in an increased hospital mortality. In this large study, hospital mortality is highest for patients who have intraabdominal infections followed by ischemic bowel and disseminated infections and lowest for those who have obstructive uropathy–associated urinary tract infections. Adjustment for some downstream factors does not explain residual variation after adjustment for predisposing factors. This suggests that even if interventions directly targeting these factors reduced mortality, heterogeneity in mortality across anatomic sources of infection would remain. Related studies have examined the role of the anatomic source in outcomes from severe sepsis [4–6] and have found that urosepsis has a more favorable prognosis, whereas abdominal sources have a worse prognosis. Although respiratory infections are more common, abdominal infections may be more severe. Volakli et al. [7,8] describe that while patients with abdominal infections have early renal and coagulation failure, patients with respiratory infections more commonly have early alteration in neurological function. The study also states that the length of hospital stay in patients with abdominal infection is longer, likely because of the increased number of secondary infections in these patients. In contrast, He et al. [9] defends that patients with pulmonary-sepsis were more prone to fungal infection, acute renal failure requiring continuous renal replacement therapy, prolonged mechanical ventilation, and a longer ICU and hospital stay. According to Bagshaw [10] urinary tract infections are a common complication of critical illness that are associated with increased patient morbidity but not with mortality. Septic shock mortality from urinary sources is reported to be lower than 10% to 20%. This rate is consistently lower than the 30% to 40% mortality reported for other common sources of septic shock [11,12]. Urinary tract infections have a better outcome than infections from other sources, as studies have shown [2,7,11]. The anatomic structure of the bladder and the genitourinary tract as well as washout by micturition may prevent bacterial invasion and limit absorption of microbes and bacterial toxins [2,13]. The main cause of urinary tract infections is obstruction of the urinary tract (obstructive pyelonephritis). Prompt action taken to solve this (drainage or lithotomy and placement of catheters) is recommended as source control [14]. There are several reasons why exploring the relationship between the source of infection and mortality due to sepsis and septic shock would be beneficial. Firstly, to improve the prognostic scoring systems, thus helping identify patients who are at a higher risk of mortality. Secondly, to identify those who could benefit from early admission to the intensive care unit and from novel treatments focusing on the management of sepsis. We should, therefore, consider the anatomical source of infection when designing studies related to sepsis and septic shock. **Impact of Source Infection in Sepsis**.

**References**

1. Vincent, J.; Opal, S.; Marshall, J. Sepsis definitions: Time for change. *Lancet* **2013**, *381*, 774–775.
2. Leligdowicz, A.; Dodek, P.M.; Norena, M.; Wong, H.; Kumar, A.; Kumar, A.; Co-operative Antimicrobial Therapy of Septic Shock Database Research Group. Association between source of infection and hospital mortality in patients who have septic shock. *Am. J. Respir. Crit. Care Med.* **2014**, *189*, 1204–1213.
3. Laffey, J.G.; Bellani, G.; Pham, T.; Fan, E.; Madotto, F.; Bajwa, E.K.; Brochard, L.; Clarkson, K.; Esteban, A.; Gattinoni, L. Potentially modifiable factors contributing to outcome from acute respiratory distress syndrome: The LUNG SAFE study. *Intensive Care Med.* **2016**, *42*, 1865–1876.
4. Rello, J.; Ricart, M.; Mirelis, B.; Quintana, E.; Gurgui, M.; Net, A.; Prats, G. Nosocomial bacteremia in a medical-surgical intensive care unit: Epidemiologic characteristics and factors influencing mortality in 111 episodes. *Intensive Care Med.* **1994**, *20*, 94–98.
5. Shorr, A.F.; Bernard, G.R.; Dhainaut, J.F.; Russell, J.R.; Macias, W.L.; Nelson, D.R.; Sundin, D.P. Protein C concentrations in severe sepsis: An early directional change in plasma levels predicts outcome. *Crit. Care* **2006**, *10*, R92–R99.
6. Blanco, J.; Muriel-Bombín, A.; Sagredo, V.; Taboada, F.; Gandía, F.; Tamayo, L.; Collado, J.; García-Labattut, A.; Carriedo, D.; Valledor, M.; et al. Incidence, organ dysfunction and mortality in severe sepsis: A Spanish multicentre study. *Crit. Care* **2008**, *12*, R158–R171.
7. Volakli, E.; Spies, C.; Michalopoulos, A.; Groeneveld, A.B.; Sakr, Y.; Vincent, J.L. Infections of respiratory or abdominal origin in ICU patients: What are the differences? *Crit. Care* **2010**, *14*, R32.
8. De Waele, J.; Lipman, J.; Sakr, Y.; Marshall, J.C.; Vanhems, P.; Barrera Groba, C.; Leone, M.; Vincent, J.L.; EPIC II Investigators. Abdominal infections in the intensive care unit: Characteristics, treatment and determinants of outcome. *BMC Infect. Dis.* **2014**, *14*, 420.
9. He, X.L.; Liao, X.L.; Xie, Z.C.; Han, L.; Yang, X.L.; Kang, Y. Pulmonary Infection Is an Independent Risk Factor for Long-Term Mortality and Quality of Life for Sepsis Patients. *Biomed. Res. Int.* **2016**, *2016*, 1–10.
10. Bagshaw, S.M.; Laupland, K.B. Epidemiology of intensive care unit-acquired urinary tract. *Curr. Opin. Infect. Dis.* **2006**, *19*, 67–71.
11. Qiang, X.H.; Yu, T.O.; Li, Y.N.; Zhou, L.X. Prognosis Risk of Urosepsis in Critical Care Medicine: A Prospective Observational Study. *Biomed. Res. Int.* **2016**, *2016*, 1–5.
12. Nicolle, L.E. Urinary Tract Infection. *Crit. Care Clin.* **2013**, *29*, 699–715.
13. Finer, G.; Landau, D. Pathogenesis of urinary tract infections with normal female anatomy. *Lancet Infect. Dis.* **2004**, *4*, 631–635.
14. Lagunes, L.; Encina, B.; Ramirez-Estrada, S. Current understanding in source control management in septic shock patients: a review. *Ann. Transl. Med.* **2016**, *4*, 330.

## Assessment of the Worldwide Burden of Critical Illness: The Intensive Care over Nations (Icon) Audit

VincentJean-LouisDepartment of Intensive Care, Erasme Hospital, Université libre de Bruxelles, Brussels, Belgium

**Abstract:** Sepsis remains a major cause of morbidity and mortality in modern intensive care units (ICUs). The true incidence of sepsis and its associated mortality are difficult to state with any precision. Aside from problems of definition, our epidemiological data are limited largely to the ICU population, but many patients with sepsis do not reach the ICU. Moreover, although there are good epidemiological data on sepsis in ICU patients in the developed world, data from other countries and areas of the world are much less complete. In 2012, the World Federation of Societies of Intensive and Critical Care Medicine (WFSICCM) conducted a worldwide audit of patient data from ICUs around the world, including aspects of infection and organ dysfunction. More than 10,000 patients were included from 84 countries worldwide. Defining sepsis as infection plus organ dysfunction, we showed important differences around the world, including international differences in occurrence rates (13–39%), causative microorganisms and outcomes (12–47%). Interestingly, a comparison of these data from 2012 and similar data collected in 2002 using the same definitions of sepsis, showed no increase in the rate of sepsis over that 10-year period, despite an increase in the severity of illness and age of ICU patients. These observational data do not support the proposal made by some that the incidence of sepsis is increasing: This is largely a reporting phenomenon related to use of definitions that effectively consider sepsis as equivalent to infection. As shown in international collaborative studies such as ICON, the heterogeneity of sepsis patterns, which is already considerable in individual patients within a single center, can be multiplied many fold when considered across centers, countries and continents. Clearly a “one size fits all” approach to management of patients with sepsis will never work; rather a personalized approach is needed such that at each instant in the course of their disease, each patient receives the correct treatment for their particular combination of infectious, immune and organ dysfunction characteristics.

**Suggested Reading**

1. Vincent, J.L.; Mira, J.P.; Antonelli, M. Sepsis: older and newer concepts. *Lancet Respir. Med.* **2016**, *4*, 237–240.
2. Vincent, J.L.; Marshall, J.C.; Namendys-Silva, S.A.; François, B.; Martin-Loeches, I.; Lipman, J.; Reinhart, K.; Antonelli, M.; Pickkers, P.; Nijmi, H. Assessment of the worldwide burden of critical illness: The intensive care over nations (ICON) audit. *Lancet Respir. Med.* **2014**, *2*, 380–386.
3. Vincent, J.L.; Lefrant, J.Y.; Kotfis, K.; et al. Comparison of European ICU patients in 2012 (ICON) versus 2002 (SOAP). *Intensive Care Med.* **2018**, in press.
4. Kaukonen, K.M.; Bailey, M.; Suzuki, S.; Pilcher, D.; Bellomo, R. Mortality related to severe sepsis and septic shock among critically ill patients in Australia and New Zealand, 2000–2012. *JAMA* **2014**, *311*, 1308–1316.
5. Rhee, C.; Dantes, R.; Epstein, L.; Murphy, D.J.; Seymour, C.W.; Iwashyna, T.J.; Kadri, S.S.; Angus, D.C.; Danner, R.L.; Fiore, A.E.; et al. Incidence and trends of sepsis in US hospitals using clinical vs. claims data, 2009–2014. *JAMA* **2017**, *318*, 1241–1249.

##                                        

 

SESSION V. “SATELLITE SYMPOSIUM: A CLOSER LOOK AT ALBUMIN”

 

## Albumin Administration for Organ Protection

WiedermannChristian J.University Hospitals Innsbruck, Tirol Kliniken GmbH, Anichstrasse 35, 6020 Innsbruck (Tyrol), Austria

**Abstract:** Albumin is a multifunctional colloid that is essential for maintaining fluid balance between intravascular and interstitial compartments. As a predominantly plasma protein, albumin accounts for approximately 75–80 percent of plasma oncotic pressure, and plays a role in ligand binding, free radical scavenging, anti-inflammatory processes, and as an antioxidant. The role of albumin in inhibition of apoptosis and as a mediator of cell signaling have also been reviewed [1]. Albumin binds directly to a number of endogenous ligands including hormones, lipids, metabolites, metal ions, and high-affinity endothelial cell receptors. Binding of albumin to endogenous ligands affects multiple biological processes including transport, sequestration, and transcytosis. Moreover, albumin binds to numerous drugs, modifying their bioavailability and pharmacokinetics [2].

**Evidence-Based Organ Protection by Human Albumin Infusion**

Initial Findings in Randomized Controlled Trials

The use of albumin for fluid resuscitation has proven valuable in a number of conditions. A 2003 systematic review of 79 randomized controlled trials (RCTs) (*N* = 4755 patients) demonstrated that albumin was beneficial in more than 70 percent of studies across a range of organ function endpoints with no detrimental effects identified [3]. In cirrhosis patients with ascites, albumin was effective at reducing hemodynamic dysfunction, morbidity and length of stay in hospital, and also reduced mortality and renal dysfunction due to spontaneous bacterial peritonitis. In cardiac surgery patients, albumin administration led to reduced pulmonary edema, lower fluid requirements, and superior hemodilution compared with crystalloids. Postoperative bleeding also decreased in response to albumin administration compared with hydroxyethyl starch (HES). Furthermore, in non-cardiac surgery, albumin reduced fluid requirements and decreased pulmonary and intestinal edema. Albumin is also associated with improved outcomes and decreased complications in patients with brain injury and those with burns. In spite of these findings, there is still doubt surrounding the value of albumin in fluid management and hypoalbuminemia. This is due in part to confounding factors in previous RCTs, such as administration of albumin to the control group and discrepancies in hemodynamic targets between groups [3,4], which may disguise true treatment effects.

**Hypoalbuminemia**

In critically-ill patients, hypoalbuminemia (serum albumin levels lower than 4.0 g/dL) and sepsis occur frequently and may be beneficially treated with albumin. Acutely-ill patients often develop hypoalbuminemia and postoperative decreases in serum albumin concentration from 3.8 ± 0.5 g·dL^−1^ to 2.6 ± 0.5 g·dL^−1^ (i.e., moderately-severe hypoalbuminemia) have been demonstrated in patients undergoing cardiopulmonary bypass [5]. Low serum albumin levels are associated with poorer outcomes, and exogenous administration of albumin is beneficial. A meta-analysis of 90 clinical studies (*N* = 291,433 patients) used multivariate analysis to evaluate hypoalbuminemia as an outcome predictor in acutely-ill patients. Each 10.0 g·L^−1^ decrease in serum albumin increased the risk of mortality and morbidity, prolonged stay in the intensive care unit and overall length of hospital stay, and increased utilization of healthcare resources [6]. Of note, the relationship between hypoalbuminemia and poorer outcomes was independent of nutritional status and inflammation. A meta-regression analysis of results from 9 prospective controlled trials (*N* = 535 patients) showed that the incidence of complications declined significantly as the serum albumin level increased following albumin supplementation [6]. In addition, a multicenter, double-blind RCT was conducted in 40 patients with severe hypoalbuminemia and acute lung injury who were receiving adjunctive diuretic therapy. Albumin significantly increased oxygenation and improved net fluid loss compared with saline. Furthermore, albumin administration led to decreased hypotension, improved Sequential Organ Failure Assessment (SOFA) scores, and increased the number of shock-free days by 7.0 days [7]. Significant effects of co-administration of albumin with furosemide on transiently increased amounts of urine volume and sodium excretion have been observed in several studies in hypoalbuminemic patients [8].

A previous systematic review analyzed the relationship between hypoalbuminemia and renal failure; in particular, the association between serum albumin and the incidence of acute kidney injury (AKI), and the impact of lower serum albumin levels on mortality in patients with AKI [9]. Hypoalbuminemia was confirmed as an independent predictor for AKI and subsequent mortality [10]. A recent trial evaluated the effect of exogenous 20% human albumin vs. saline on the incidence of postoperative AKI in adult patients with hypoalbuminemia undergoing off-pump coronary artery bypass surgery [11]. The rate of postoperative AKI increased in the saline group as the level of serum albumin decreased. In addition, the incidence of postoperative AKI was significantly lower in the albumin group, and multivariate logistic regression analysis identified a renal-protective effect of albumin administration [11]. The inverse relationship between serum albumin and postoperative AKI confirms the positive association between albumin and renal health. In line with this, correction of hypoalbuminemia resulted in smaller increases in serum creatinine and led to protection against AKI.

Supporting evidence for possible renal protection by albumin administration has been recently provided by a large retrospective cohort study confirming hypoalbuminemia on admission to the hospital as a risk factor for the development of AKI and mortality [12]. A total of 19,472 patients were enrolled and included in a hypoalbuminemia group (serum albumin level <3.4 mg/dL, measured within two days of admission) and a normal group. AKI was defined as an increase in serum creatinine levels of ≥0.3 mg/dL or 1.5 times baseline during hospitalization. The incidence of AKI was 10.7% in the group with hypoalbuminemia and 4.1% in the control group; interestingly, in patients with AKI, albumin administration significantly improved renal recovery [12].

**Albumin Isoforms and Reduced Effective Albuminemia in Severe Liver Disease**

In patients with cirrhosis, serum albumin is post-transcriptionally changed which is likely to affect its function. In a study aimed at identifying cirrhosis-related structural changes of serum albumin and their effects on patient morbidity and mortality, it was shown that the albumin isoforms are not only altered in liver cirrhosis, but are also increasingly impaired in their function, and impairment parallels the severity of liver disease. Altered isoforms including cysteinylated/N-terminal truncated albumin are therefore associated with specific clinical complications, making them better predictors of patient survival than the total albumin concentration. These findings support the concept of ‘effective’ albumin concentration, which implies that the global function of human albumin is not only related to serum concentration, but also to the maintenance of structural integrity [13].

Cirrhosis-associated immunosuppression is held responsible for both infectious and non-infectious, inflammatory complications of the disease [14,15]. Prostaglandin E_2_ (PGE_2_) levels are elevated in cirrhosis and mediate immunosuppression; human albumin infusion can be used to successfully reduce circulating PGE_2_ levels, attenuate immunosuppression, and reduce the risk of infection in acutely decompensated cirrhotics [16].

The potential of organ protection by albumin use has been further illustrated recently at the International Liver Congress 2017 held in Amsterdam, The Netherlands [17]: The ANSWER study was a randomized, controlled trial of 440 patients with cirrhosis and uncomplicated ascites that compared standard diuretic therapy with standard diuretic therapy plus human albumin (40 g intravenously twice weekly in the first two weeks and then once weekly). The primary endpoint was overall survival and patients were followed for up to 18 months: “The rate of survival was significantly higher in patients receiving human albumin plus to standard therapy, compared with those receiving standard therapy only. Treatment with human albumin reduced the risk of death by 38%. Statistically significant benefits of administering human albumin rather than standard therapy alone were demonstrated for the management of ascites, complications of cirrhosis, quality of life and hospital admissions [17]”.

Since treatment with human albumin, especially in spontaneous bacterial peritonitis and hepato-renal syndrome, has significantly reduced mortality, it is currently the only colloid administration recommended in both, international and national guidelines, e.g., the European EASL guidelines [18], the German S3 guidelines [19] and the revised edition of the German cross-sectional guidelines for the treatment with blood components and plasma derivatives [20].

Hepatic encephalopathy (HE), a neuropsychiatric syndrome complicating acute and chronic liver failure in the absence of other brain disease, is a condition in which use of albumin may be organ protective as well. The effects of albumin administration in patients with HE have been investigated in two small RCTs both describing significantly increased survival rates in the albumin-treated groups; mortality was defined as a secondary study endpoint. However, reported results from the two studies were conflicting for various primary outcome parameters [21,22]. Further studies to explore the potential of such a therapeutic approach are needed.

**Sepsis**

With regards to sepsis/septic shock, the ALBIOS study analyzed the effects of albumin maintenance at 3.0 g/dL for up to 28 days during ICU stay in patients with severe sepsis or septic shock (*N* = 1818) [23]. There were no significant differences in overall survival after 28 or 90 days between the albumin and crystalloid groups. However, a *post-hoc* analysis showed that in the subgroup of 1300 patients with septic shock, there was a significant survival advantage after 90 days in patients who received albumin. In addition to its hemodynamic effects, albumin may have a protective effect on endothelial function and microcirculation. This is evidenced by the loss of glycocalyx that follows decreases in plasma albumin concentration, leading to increased fluid extravasation [24]. Albumin is shown to protect against loss of glycocalyx in models of ischemia-reperfusion and facilitated recovery of the glycocalyx in cases of hemorrhagic shock (for review see [25–27]).

Human albumin is the only colloid infusion recommended in guidelines for use in severe sepsis and septic shock [28–30]. Its administration is suggested when in volume resuscitation a combination of crystalloids with colloids is considered necessary for the treatment of patients in septic shock (grade 2C).

**Conclusions**

Evidence in support of organ protection by administration of human albumin is growing. Known beneficial effects of albumin are mediated by both hemodynamic and pharmacodynamic mechanisms. The hemodynamic effects of albumin are important for normal organ perfusion and function, while exogenous administration of human albumin helps to maintain glomerular filtration and preserve renal function via hemodynamic and oncotic mechanisms. Pharmacodynamic properties include mitigation of nephrotoxicity induced by certain medications, restoration of net fluid balance, protection against loss of glycocalyx, and maintenance of glomerular filtration. Reduced renal morbidity in patients with liver cirrhosis and decreased nephrotoxicity suggest a nephro-protective effect of human albumin in addition to its unique pharmacodynamic properties. Important roles of albumin administration in regulating innate immune system and inflammation in states of effective hypoalbuminemia such as cirrhosis and sepsis are increasingly uncovered. However, more high-quality, randomized, interventional studies are required to better understand the mechanisms by which albumin leads to organ protection and to identify additional clinical indications in areas where current evidence is still too low to better inform clinical practice.

**Conflicts of Interest:** The author has received fees for speaking and/or travel cost reimbursements from providers’ educational grants by Kedrion, CSL, Grifols, Baxter and Daiichi Sankyo.

**References**

1. Quinlan, G.J.; Martin, G.S.; Evans, T.W. Albumin: Biochemical properties and therapeutic potential. *Hepatology* **2005**, *41*, 1211–1219.
2. Bertucci, C.; Domenici, E. Reversible and covalent binding of drugs to human serum albumin: Methodological approaches and physiological relevance. *Curr. Med. Chem.* **2002**, *9*, 1463–1481.
3. Haynes, G.R.; Navickis, R.J.; Wilkes, M.M. Albumin administration--what is the evidence of clinical benefit? A systematic review of randomized controlled trials. *Eur. J. Anaesthesiol.* **2003**, *20*, 771–793.
4. Vincent, J.L.; Navickis, R.J.; Wilkes, M.M. Morbidity in hospitalized patients receiving human albumin: A meta-analysis of randomized, controlled trials. *Crit. Care Med.* **2004**, *32*, 2029–2038.
5. Mohnle, P.; Schwann, N.M.; Vaughn, W.K.; Snabes, M.C.; Lau, W.; Levin, J.; Nussmeier, N.A. Perturbations in laboratory values after coronary artery bypass graft surgery with cardiopulmonary bypass. *J. Cardiothorac. Vasc. Anesth.* **2005**, *19*, 19–25.
6. Vincent, J.L.; Dubois, M.J.; Navickis, R.J.; Wilkes, M.M. Hypoalbuminemia in acute illness: Is there a rationale for intervention? A meta-analysis of cohort studies and controlled trials. *Ann. Surg.* **2003**, *237*, 319–334.
7. Martin, G.S.; Moss, M.; Wheeler, A.P.; Mealer, M.; Morris, J.A.; Bernard, G.R. A randomized, controlled trial of furosemide with or without albumin in hypoproteinemic patients with acute lung injury. *Crit. Care Med.* **2005**, *33*, 1681–1687.
8. Kitsios, G.D.; Mascari, P.; Ettunsi, R.; Gray, A.W. Co-administration of furosemide with albumin for overcoming diuretic resistance in patients with hypoalbuminemia: A meta-analysis. *J. Crit. Care* **2014**, *29*, 253–259.
9. Wiedermann, C.J.; Wiedermann, W.; Joannidis, M. Hypoalbuminemia and acute kidney injury: A meta-analysis of observational clinical studies. *Intensive Care Med.* **2010**, *36*, 1657–1665.
10. Wiedermann, C.J.; Wiedermann, W.; Joannidis, M. Causal relationship between hypoalbuminemia and acute kidney injury. *World J. Nephrol.* **2017**, *6*, 176–187.
11. Lee, E.H.; Kim, W.J.; Kim, J.Y.; Chin, J.H.; Choi, D.K.; Sim, J.Y.; Choo, S.J.; Chung, C.H.; Lee, J.W.; Choi, I.C. Effect of exogenous albumin on the incidence of postoperative acute kidney injury in patients undergoing off-pump coronary artery bypass surgery with a preoperative albumin level of less than 4.0 g/dL. *Anesthesiology* **2016**, *124*, 1001–1011.
12. Yu, M.Y.; Lee, S.W.; Baek, S.H.; Na, K.Y.; Chae, D.W.; Chin, H.J.; Kim, S. Hypoalbuminemia at admission predicts the development of acute kidney injury in hospitalized patients: A retrospective cohort study. *PLoS ONE* **2017**, *12*, e0180750.
13. Domenicali, M.; Baldassarre, M.; Giannone, F.A.; Naldi, M.; Mastroroberto, M.; Biselli, M.; Laggetta, M.; Patrono, D.; Bertucci, C.; Bernardi, M.; et al. Posttranscriptional changes of serum albumin: Clinical and prognostic significance in hospitalized patients with cirrhosis. *Hepatology* **2014**, *60*, 1851–1860.
14. Philips, C.A.; Sarin, S.K. Sepsis in cirrhosis: Emerging concepts in pathogenesis, diagnosis and management. *Hepatol. Int.* **2016**, *10*, 871–882.
15. Clària, J.; Arroyo, V.; Moreau, R. The acute-on-chronic liver failure syndrome, or when the innate immune system goes astray. *J. Immunol.* **2016**, *197*, 3755–3761.
16. O’Brien, A.J.; Fullerton, J.N.; Massey, K.A.; Auld, G.; Sewell, G.; James, S.; Newson, J.; Karra, E.; Winstanley, A.; Alazawi, W.; et al. Immunosuppression in acutely decompensated cirrhosis is mediated by prostaglandin E_2_. *Nat. Med.* **2014**, *20*, 518–523.
17. Bernardi, M. *Long-Term Albumin Administration Improves Survival in Patients with Decompensated Cirrhosis: Final Results of the “ANSWER” Study*; The International Liver Congress™: Amsterdam, The Netherlands, 2017. Available online: https://ilc-congress.eu/wp-content/uploads/2017/04/LBO-008-Bernardi.pdf (accessed on 6 January 2018).
18. European Association for the Study of the Liver. EASL clinical practice guidelines on the management of ascites, spontaneous bacterial peritonitis, and hepatorenal syndrome in cirrhosis. *J. Hepatol.* **2010**, *53*, 397–417.
19. Gerbes, A.L.; Gülberg, V.; Sauerbruch, T.; Wiest, R.; Appenrodt, B.; Bahr, M.J.; Dollinger, M.; Rössle, M.; Schepke, M. German S 3-guideline “ascites, spontaneous acterial peritonitis, hepatorenal syndrome”. *Z. Gastroenterol.* **2011**, *49*, 749–779.
20. Vorstand der Bundesärztekammer. Querschnitts-Leitlinien (BÄK) zur Therapie Mit Blutkomponenten und Plasmaderivaten. 4. Überarbeitete und Aktualisierte Auflage. 2014. Available online: http://www.bundesaerztekammer.de/fileadmin/user_upload/downloads/QLL_Haemotherapie_2014.pdf (accessed on 6 January 2018).
21. Jalan, R.; Kapoor, D. Reversal of diuretic-induced hepatic encephalopathy with infusion of albumin but not colloid. *Clin. Sci.* **2004**, *106*, 467–474.
22. Simon-Talero, M.; García-Martínez, R.; Torrens, M.; Augustin, S.; Gómez, S.; Pereira, G.; Guevara, M.; Ginés, P.; Soriano, G.; Román, E. Effects of intravenous albumin in patients with cirrhosis and episodic hepatic encephalopathy: A randomized double-blind study. *J. Hepatol.* **2013**, *59*, 1184–1192.
23. Caironi, P.; Tognoni, G.; Masson, S.; Fumagalli, R.; Pesenti, A.; Romero, M.; Fanizza, C.; Caspani, L.; Faenza, S.; Grasselli, G.; et al. Albumin replacement in patients with severe sepsis or septic shock. *N. Engl. J. Med.* **2014**, *370*, 1412–1421.
24. Stevens, A.P.; Hlady, V.; Dull, R.O. Fluorescence correlation spectroscopy can probe albumin dynamics inside lung endothelial glycocalyx. *Am. J. Physiol. Lung Cell. Mol. Physiol.* **2007**, 293, L328–L335.
25. Vincent, J.L.; De Backer, D.; Wiedermann, C.J. Fluid management in sepsis: The potential beneficial effects of albumin. *J. Crit. Care* **2016**, *35*, 161–167.
26. Wiedermann, C.J.; Joannidis, M. Nephroprotective potential of human albumin infusion: A narrative review. *Gastroenterol. Res. Pract.* **2015**, *2015*, 1–8.
27. Tarbell, J.M.; Cancel, L.M. The glycocalyx and its significance in human medicine. *J. Intern. Med.* **2016**, *280*, 97–113.
28. Reinhart, K.; Perner, A.; Sprung, C.L.; Jaeschke, R.; Schortgen, F.; Johan Groeneveld, A.B.; Beale, R.; Hartog, C.S.; European Society of Intensive Care Medicine. Consensus statement of the ESICM task force on colloid volume therapy in critically ill patients. *Intensive Care Med.* **2012**, *38*, 368–383.
29. Rhodes, A.; Evans, L.E.; Alhazzani, W.; Levy, M.M.; Antonelli, M.; Ferrer, R.; Kumar, A.; Sevransky, J.E.; Sprung, C.L.; Nunnally, M.E.; et al. Surviving Sepsis Campaign: International Guidelines for Management of Sepsis and Septic Shock: 2016. *Intensive Care Med.* **2017**, *45*, 486–552.
30. Joannidis, M.; Druml, W.; Forni, L.G.; Groeneveld, A.B.J.; Honore, P.M.; Hoste, E.; Ostermann, M.; Oudemans-van Straaten, H.M.; Schetz, M. Prevention of acute kidney injury and protection of renal function in the intensive care unit: Update 2017: Expert opinion of the Working Group on Prevention, AKI section, European Society of Intensive Care Medicine. *Intensive Care Med.* **2017**, *43*, 730–749.

## Do We Need Another Trial?

VincentJean-LouisDepartment of Intensive Care, Erasme Hospital, Université libre de Bruxelles, Brussels, Belgium

**Abstract:** Fluids play a key role in the management of critically ill patients, but multiple aspects of fluid management are still under debate, including the precise role—if any—of albumin. Colloid solutions are of particular value in patients requiring large volumes of crystalloids because less colloid volume is needed to reach the same hemodynamic effect, thus reducing the likelihood of fluid overload and its associated harmful effects on multiple outcomes. Human albumin is the only available non-synthetic colloid. In addition to its oncotic properties, human albumin has other physiological roles, including anti-oxidant effects, protective effects on vessel wall integrity (protecting the glycocalyx), and as a carrier protein, which may be of particular benefit in certain groups of patients, notably those with sepsis and/or hypoalbuminemia. The recent Surviving Sepsis Campaign recommendations suggest the administration of human albumin solutions in addition to crystalloids for initial resuscitation and subsequent intravascular volume replacement in patients with sepsis and septic shock when patients require substantial amounts of crystalloids. The most recent American College pediatric guidelines recommend either crystalloids or albumin solutions. Although more expensive than other colloid solutions, the price of albumin relative to other treatments has decreased over recent years. Whether albumin administration can decrease mortality rates has not been shown definitely, but this is true for virtually any intervention in the ICU: this is our current problem with clinical trials. So, do we need another trial? The simple answer is that I’m not sure whether this is necessary or worthwhile. Clearly albumin is not indicated in all patients and, if conducted, such a trial should include only those patients most likely to benefit, i.e., patients with septic shock. In addition, it should not focus simply on the very short-term use of albumin during the acute resuscitation phase, but rather on the more prolonged use of albumin as supplementation to correct hypoalbuminemia. Importantly too, being able to demonstrate an effect of albumin on mortality rates is unlikely and effects on organ (dys)function may be more valuable. A large German study evaluating the effects of albumin in septic shock is being planned. Nevertheless, the results may still not provide us with any definitive answers as patients with septic shock remain a heterogeneous population. Fluid administration in critically ill patients must be personalized to the individual patient according to multiple factors, including severity of illness and underlying diagnosis.

**Suggested Reading**

1. Vincent, J.L.; Russell, J.A.; Jacob, M.; Martin, G.; Guidet, B.; Wernerman, J.; Ferrer-Roca, R.; McCluskey, S.A.; Gattinoni, L. Albumin administration in the acutely ill: What is new and where next? *Crit. Care* **2014**, *18*, 231.
2. Caironi, P.; Tognoni, G.; Masson, S.; Fumagalli, R.; Pesenti, A.; Romero, M.; Fanizza, C.; Caspani, L.; Faenza, S.; Grasselli, G.; et al. Albumin replacement in patients with severe sepsis or septic shock. *N. Engl. J. Med.* **2014**, *370*, 1412–1421.
3. Xu, J.Y.; Chen, Q.H.; Xie, J.F.; Pan, C.; Liu, S.Q.; Huang, L.W.; Yang, C.S.; Liu, L.; Huang, Y.Z.; Guo, F.M.; et al. Comparison of the effects of albumin and crystalloid on mortality in adult patients with severe sepsis and septic shock: A meta-analysis of randomized clinical trials. *Crit. Care* **2014**, *18*, 702.
4. Finfer, S.; McEvoy, S.; Bellomo, R.; McArthur, C.; Myburgh, J.; Nortno, R.; SAFE Study Investigators. Impact of albumin compared to saline on organ function and mortality of patients with severe sepsis. *Intensive Care Med.* **2011**, *37*, 86–96.
5. Vincent, J.L.; De Backer, D.; Wiedermann, C.J. Fluid management in sepsis: The potential beneficial effects of albumin. *J. Crit. Care* **2016**, *35*, 161–167.

##                                        

 

SESSION VI. “CAVEATS IN RESPIRATORY INFECTIONS”

 

## Aspergillus Significance in Non Immunocompromised Patients

RogersThomas R.Department of Clinical Microbiology, Trinity College Dublin & St James’s Hospital, Dublin, Ireland; rogerstr@tcd.ie

**Abstract:** Invasive aspergillosis is a frequently lethal disease that typically affects patients who are immunocompromised. According to the European Organization for Research and Treatment of Cancer/Invasive Fungal Infections Cooperative Group and the National Institute of Allergy and Infectious Diseases Mycoses Study Group (EORTC/MSG) Consensus Group definitions of invasive fungal disease [1] proven invasive aspergillosis (IA) requires detection of fungal hyphae by histology, cytology or direct microscopy, or isolation of the mould from culture of sterile material. Probable IA requires at least one the following host factors: recent history of neutropenia, receipt of an allogeneic stem cell transplant; prolonged use of corticosteroids at a mean minimum dose of 0.3 mg/kg/day of prednisone equivalent for >3 weeks; treatment with other recognized T cell immunosuppressants, such as cyclosporine, TNF-*α* blockers, specific monoclonal antibodies (such as alemtuzumab), or nucleoside analogues during the past 90 days, or inherited severe immunodeficiencies. In mixed medical/surgical intensive care units (ICU) only a minority of patients will fall within these specifically immunocompromised groups. Over the past decade there has been a growing realisation that there are critical care patients succumbing to IA who do not fall within these traditional categories of immunocompromised patients. Because of a lack of consensus on criteria for diagnosis of IA in these patients there are delays in recognition of IA and consequent high mortality rates. This statement is supported by the few studies where autopsies have revealed the presence of IA that was not diagnosed earlier during critical care. In an effort to improve detection of probable cases (which they termed putative IA), Blot et al. [2] proposed a clinical algorithm that required, as an entry criterion, an *Aspergillus*-positive sample from a lower respiratory tract specimen culture, combined with a set of clinical, radiological and host criteria. Notably *Aspergillus* biomarkers such as serum or bronchoalveolar lavage (BAL) galactomannan were not included. Taccone et al. [3] used these criteria in a study conducted in 30 ICU’s where they identified 297 cases of proven or putative IA. They found that 70% of the cases were immunosuppressed in accordance with the EORTC/MSG criteria. When they compared these patients with those who were not immunosuppressed the following were significantly associated with IA in the latter group (*p* < 0.05): diabetes, smoking history, alcohol abuse, acute heart failure and renal replacement therapy. Chronic obstructive pulmonary disease (COPD) is increasingly recognised as a risk factor for IA, especially in patients with more advanced Global initiative for Obstructive Lung Disease (GOLD) stage disease and/or who have received prolonged courses of corticosteroids. It has been shown that COPD patients colonized by *Aspergillus* have a high risk of later progressing to invasive disease. Other patients with increased risk of IA are those with liver cirrhosis, acute respiratory distress syndrome, severe sepsis, and acute renal failure [4]. Recently there have been reports of influenza virus infection predisposing to IA. Van de Veerdonk et al. [5] report a 16% incidence of influenza-associated aspergillosis in critically ill ICU patients. Of interest was that no patients were neutropenic, not all of the patients had received corticosteroids, and 30% had no underlying risk factors for IA; there was a high associated mortality. As more experience is gained with managing critical care patients at risk of IA, our ability to make a confident diagnosis, at least of probable/putative IA, should improve. The use of diagnostic biomarkers like BAL galactomannan and *Aspergillus* PCR is becoming more established and is being incorporated into critical care pathways. Early empirical antifungal therapy will continue to be practised but use of serum 1–3-beta-D glucan monitoring should help to curtail overuse of systemic antifungals because of its high negative predictive value [4]. A further consideration is the emergence of antifungal drug resistance in the main pathogenic species *A. fumigatus* and the greater possibility of drug resistance in other *Aspergillus* species [6]. This necessitates having ready access to excellent mycology laboratory support. A final consideration is control of the environment to minimise the risk of nosocomially acquired IA. Recent national guidelines have been published that address this specific topic and are available at http://www.hpsc.ie/a-z/respiratory/aspergillosis/guidance/.

**References**

1. De Pauw, B.; Walsh, T.J.; Donnelly, J.P.; Stevens, D.A.; Edwards, J.E.; Calandra, T.; Pappas, P.G.; Maertens, J.; Lortholary, O.; Kauffman, C.A.; et al. Revised definitions of invasive fungal disease from the European Organization for Research and Treatment of Cancer/Invasive Fungal Infections Cooperative Group and the National Institute of Allergy and Infectious Diseases Mycoses Study Group (EORTC/MSG) Consensus Group. *Clin. Infect. Dis.* **2008**, *46*, 1813–1821, doi:10.1086/588660.
2. Blot, S.I.; Taccone; FS; Van den Abeele, A.-M.; Bulpa, P.; Meersseman, W.; Brusselaers, N.; Dimopoulos, G.; Paiva, J.A.; Misset, B.; Rello, J.; et al. A clinical algorithm to diagnose invasive pulmonary aspergillosis in critically ill patients. *Am. J. Respir. Crit. Care Med.* **2012**, *186*, 808.
3. Taccone, F.S.; Van den Abeele, A.-M.; Bulpa, P.; Misset, B.; Meersseman, W.; Cardoso, T.; Paiva, J.A.; Blasco-Navalpotro, M.; De Laere, E.; Dimopoulos, G.; et al. Epidemiology of invasive aspergillosis in critically ill patients: Clinical presentation, underlying conditions, and outcomes. *Crit. Care* **2015**, *19*, 7, doi:10.1186/s13054–014-0722–7.
4. Bassetti, M.; Peghin, M.; Vena, A. Challenges and Solution of Invasive Aspergillosis in Non-neutropenic Patients: A Review. *Infect. Dis. Ther.* **2017**, doi:10.1007/s40121–017-0183–9.
5. Van de Veerdonk, F.L.; Kolwijck, E.; Lestrade, P.P.; Hodiamont, C.J.; Rijnders, B.J.; van Paassen, J.; Haas, P.J.; Oliveria Dos Santos, C.; Kampinga, G.A.; Bergmans, D.C.; et al. Influenza-Associated Aspergillosis in Critically Ill Patients. *Am. J. Respir. Crit. Care Med.* **2017**, doi:10.1164/rccm.201612–2540LE.
6. Lass-Florl, C.; Cuenca-Estrella, M. Changes in the epidemiological landscape of invasive mould infections and disease. *J. Antimicrob. Chemother.* **2017**, *72* (Suppl. 1), i5–i11, doi:10.1093/jac/dkx028.

## Ineffective Measures to Prevent Ventilator-Associated Pneumonia

NseirSaad^1^,^2^1CHU Lille, Critical Care Center, F-59000 Lille, France; s-nseir@chru-lille.fr; Tel.: +(33)-320444084; Fax: +(33)-3204450942Medicine School, University Lille, F-59000 Lille, France

**Abstract:** In spite of important progress in the field of ventilator-associated pneumonia (VAP) pathophysiology and prevention, this infection is still common in critically ill patients. Recognizing ineffective measures for VAP prevention is helpful to avoid side effects of these interventions, and to reduce cost, and workload in the intensive care unit (ICU).

**Residual Gastric Volume Measurement**

A recent randomized, noninferiority, open-label, multicenter trial was performed in 449 adults requiring invasive mechanical ventilation, and compared absence of residual gastric volume monitoring vs. measurement of gastric volume every 6 h [1]. VAP rate was similar in intervention (16.7%) and control group (15.8%) (difference, 0.9%; 90% CI, −4.8% to 6.7%). In addition, proportion of patients receiving 100% of their calorie goal was higher in the intervention group (odds ratio, 1.77; 90% CI, 1.25–2.51; *p* = 0.008), suggesting that this measurement should not be performed.

**Systemic or Inhaled Antibiotic Therapy**

Few studies have assessed antibiotics as preemptive therapy for VAP aiming either to suppress colonization or to treat ventilator-associated-tracheobronchitis (VAT), which is considered to be a condition that may lead to VAP [2]. Systemic use of short courses of antibiotics early after intubation, or later at VAT diagnosis, or the local application of antibiotics in the trachebronchial tree showed promising results for reducing VAP prevalence [2,3]. However, available studies are small, observational, or performed in specific populations such as patients with neurologic failure. Moreover, there is still skepticism for the emergence of multidrug resistant bacteria following the implementation of such strategies.

A recent single-center randomized controlled trial evaluated the impact of inhaled colistin on VAP incidence [4]. Although the rate of VAP was lower in intervention compared with control group, the difference did not reach statistical significance.

**Statins**

Statins (reductase inhibitors) present antiinflammatory-immunomodulatory properties besides their ability to affect cholesterol composition and it has been hypothesized that they might be useful in improving the outcome or the incidence of various diseases, including pneumonia, acute respiratory distress syndrome. However, data from national registries or post hoc analysis of data from randomized studies reported conflicting results for the impact of the use of statins on the risk of pneumonia. A randomized study in critical care patients, without previous use of statins, reported that the prophylactic use of a 30-day treatment with pravastatin did not significantly modify the risk of VAP [5].

**Closed Tracheal Suctioning System**

Closed tracheal suctioning system (CTSS) might reduce exogenous tracheobronchial colonization via fewer manipulations by healthcare workers. However, 3 large randomized controlled trials did not report significant benefit of this system in reducing VAP incidence. By contrast, a recent meta analysis of 15 randomized controlled trials reported significant reduction in VAP rate using CTSS, compared with open tracheal suctioning system (OTSS) (RR 0.69; 95% CI 0.54–0.87) [6]. However, heterogeneity was high (I2 = 46.4%), and sensitivity analyses, including trial sequential analysis, suggested the scarcity of high-quality trials.

**Improved Tracheal Cuff**

In vitro and observational studies suggested reduced microaspiration and VAP rates using tracheal tubes with tapered and/or polyurethane cuff. However, the large multicenter randomized controlled TOPCUFF study [7] carefully evaluated the impact of tapered vs. tapered shape, and polyurethane vs. polyvinyl chloride cuff in 621 patients receiving mechanical ventilation for >48 h. No significant impact of these interventions was found on tracheobronchial colonization, or VAP rates. Recently, the multicenter cluster randomized controlled BestCuff trial [8] evaluated the impact of tapered cuff on microaspiration in 342 critically ill patients. No significant impact was found on microaspiration of gastric contents or oropharyngeal secretions. VAP and ventilator associated events rates were also similar in intervention and control groups.

**References**

1. Reigner, J.; Mercier, E.; Le Gouge, A.; Boulain, T.; Desachy, A.; Bellec, F.; Clavel, M.; Frat, J.P.; Plantefeve, G.; Quenot, J.P.; et al. Effect of Not Monitoring Residual Gastric Volume on Risk of Ventilator-Associated Pneumonia in Adults Receiving Mechanical Ventilation: A Randomized Controlled Trial. *JAMA* **2013**, *309*, 249–256.
2. Nseir, S.; Favory, R.; Jozefowicz, E.; Decamps, F.; Dewavrin, F.; Brunin, G.; Di Pompeo, C.; Mathieu, D.; Durocher, A.; VAT Study Group. Antimicrobial treatment for ventilator-associated tracheobronchitis: A randomized, controlled, multicenter study. *Crit. Care* **2008**, *12*, R62.
3. Bouza, E.; Granda, M.J.P.; Hortal, J.; Barrio, J.M.; Cercenado, E.; Muñoz, P. Pre-emptive broad-spectrum treatment for ventilator-associated pneumonia in high-risk patients. *Intensive Care Med.* **2013**, *39*, 1547–1555.
4. Karvouniaris, M.; Makris, D.; Zygoulis, P.; Triantaris, A.; Xitsas, S.; Mantzarlis, K.; Petinaki, E. ZE: Nebulized colistin for ventilator-associated pneumonia preventione. *Eur. Respir. J.* **2015**, *46*, 1732–1739.
5. Makris, D.; Manoulakas, E.; Komnos, A.; Papakrivou, E.; Tzovaras, N.; Hovas, A.; Zintzaras, E.; Zakynthinos, E. Effect of pravastatin on the frequency of ventilator-associated pneumonia and on intensive care unit mortality: Open-label, randomized study. *Crit. Care Med.* **2011**, *39*, 2440–2446.
6. Kuriyama, A.; Umakoshi, N.; Fujinaga, J.; Takada, T. Impact of closed versus open tracheal suctioning systems for mechanically ventilated adults: A systematic review and meta-analysis. *Intensive Care Med.* **2014**, *41*, 402–411.
7. Philippart, F.; Gaudry, S.; Quinquis, L.; Lau, N.; Ouanes, I.; Touati, S.; Nguyen, J.C.; Branger, C.; Faibis, F.; Mastouri, M.; et al. Randomized Intubation with Polyurethane or Conical Cuffs to Prevent Pneumonia in Ventilated Patients. *Am. J. Respir. Crit. Care Med.* **2015**, *191*, 637–645.
8. Jaillette, E.; Girault, C.; Brunin, G.; Zerimech, F.; Behal, H.; Chiche, A.; Broucqsault-Dedrie, C.; Fayolle, C.; Minacori, F.; Alves, I.; et al. Impact of tapered-cuff tracheal tube on microaspiration of gastric contents in intubated critically ill patients: A multicenter cluster-randomized cross-over controlled trial. *Intensive Care Med.* **2017**, *43*, 1362–71.

## Oxygen and Vap: How Much, How Long and by What Method?

CarelliS.De PascaleG.AntonelliM.Department of Anesthesiology and Intensive Care Medicine, Università Cattolica del Sacro Cuore—Fondazione Policlinico Universitario A.Gemelli, Rome, Italy

**Abstract:** Ventilator-associated pneumonia (VAP) is a nosology entity that occurs in patients mechanically ventilated (MV) for at least 48 h during their intensive care unit (ICU) stay [1]. VAP is the most common ICU-acquired infection that increases mortality, prolonging MV, ICU stay and hospitalization [2]. The identification of the risk factors and treatments of this condition are key issues in critically ill patients. Hypoxemia is often one of the earlier signs of VAP, depending in large part on the amount of lung tissue impaired by the inflammatory process. Hypoxemia is generally defined as a SaO_2_ <90% and a PaO_2_ <60 mmHg if breathing ambient air or a PaO_2_/FiO_2_ <300 [3]. Current guidelines suggest to achieve normal/near-normal SaO_2_ for all acutely ill patients, including those with VAP. Recommended target SaO_2_ range for critically ill patients is 94–98% (88–92% for those at risk of hypercapnic respiratory failure) [3]. Weaker recommendations are available about PaO_2_ even though the suggested target range is between 70 and 100 mmHg. Different strategies have been proposed to improve blood oxygenation in VAP patients, the administration of oxygen as first. It has been suggested to use the lowest possible FiO_2_ to maintain PaO_2_ between 70 and 100 mm Hg and SaO_2_ between 94% and 98%. In MV patients, the interfaces to administer oxygen are mostly mask during non invasive ventilation (NIV), and the endotracheal tube or tracheostomy cannula for invasive MV. High flow oxygen therapy (HFOT) has been recently shown improving oxygenation and generating modest levels of positive airway pressure [4]. The use of a positive end expiratory pressure (PEEP) is another cornerstone. It decreases the amount of opening and closing lung tissue and prevents lung atelectasis, reducing the ventilator induced lung injury (VILI) and attenuating the bacterial growth and translocation that may represent risk factors for VAP [5]. The application of prophylactic PEEP has been shown to reduce the number of hypoxemic episodes and incidence of VAP also in non-hypoxemic MV patients [6]. Patients positioning is another important factor; prone positioning is well known to improve oxygenation by homogenizing the transpulmonary gradient and increasing ventilation to the dorsal areas of the lungs, with the additional effect of preventing or reducing VILI [7]. Result of prone positioning for VAP prevention are however conflicting, and in ICU patients with pure hypoxemic acute respiratory failure, prone positioning had no effect on the risk of VAP [8]. Differently the semi-recumbent position still remains an effective measure for VAP prevention.

The other face of the coin is the liberal oxygen therapy that induces hyperoxia. It has been demonstrated that ICU MV patients still spend most of their time with unnecessary high FiO_2_ and PaO_2_ [9,10] despite that evidences from recent studies suggest that hyperoxemia is generally not safe. Detrimental effects related to the too high oxygen tension have been shown in perioperative patients [11], as well as in patients with ST segment elevation and myocardial infarction [12] and in patients undergoing resuscitation from cardiac arrest [13]. In a recent RCT in a medical-surgical ICU [14], oxygen supplementation titrated to a conservative SaO_2_ and PaO_2_ target (94–98% and 70–100 mmHg respectively) was associated with lower ICU and hospital mortality compared to conventional oxygen administration. Even though the occurrence of new respiratory failures and infections did not differ between the two groups, patients in the conservative group showed an increase in MV-free hours reducing the duration of MV that is a well-known factor risk for VAP [15]. In addition the study excluded patients with immunosuppression, ARDS, and COPD whose risk factors for VAP are higher, impeding a definitive conclusion on the potential benefit of a conservative oxygen therapy on the occurrence of new respiratory infections and VAP [14]. In spite of these data, what constitutes the safe upper limits and duration of FiO_2_ and PaO_2_ still remains uncertain. Available evidence shows a rapid increment of toxicity when FiO_2_ is increased above 0.6 and also when the exposure time is prolonged, showing a U-shaped relationship between mortality and PaO_2_. Mild hyperoxemia is defined as PaO_2_ between 120 and 200 mmHg and severe hyperoxemia as PaO_2_ >200 mmHg [16]. In humans, direct lung toxicity is the best-known harmful consequence of hyperoxia; it is mediated by excessive production of reactive oxygen species either through direct damage to tissue constituents or via altered oxidative signalling [17]. Hyperoxia has been reported to cause pulmonary edema, generation of hyaline membrane, pulmonary arteriole thickening, and alteration of the ventilation to perfusion ratio generating the so called hyperoxic acute lung injury (HALI [18,19]. Denitrogenation phenomena and inhibition of surfactant production are also observed at high levels of FiO_2_, promoting expiratory collapse and atelectasis [20,21]. Prolonged hyperoxia may then generate HALI inducing a reduced bacterial clearance and impairment of macrophages activity [22–24]. Animal studies suggested a close relationship between hyperoxemia and VAP [25], Six et al. designed a retrospective observational study to determine the actual relationship between hyperoxemia (defined as PaO_2_ >120 mmHg) and VAP in humans. Hyperoxemia on ICU admission, number of days with hyperoxemia and percentage of days with hyperoxemia were all more frequent in patients developing VAP and independently associated with VAP onset at the multivariate analysis.

In conclusion, management of patients affected by or at risk of VAP implies an accurate equilibrium between improving blood oxygenation and preventing hyperoxemia.

**References**

1. Torres, A.; Niederman, M.S.; Chastre, J.; Ewig, S.; Fernandez-Vandelios, P.; Hanberger, H.; Kollef, M.; Li Bassi, G.; Luna, C.M.; Martin-Loeches, I.; et al. International ERS/ESICM/ESCMID/ALAT guidelines for the management of hospital-acquired pneumonia and ventilator-associated pneumonia. *Eur. Respir. J.* **2017**, *50*, 1700582.
2. Nair, G.B.; Niederman, M.S. Ventilator-associated pneumonia: Present understanding and ongoing debates. *Intensive Care Med.* **2014**, *41*, 34–48.
3. O’Driscoll, B.R.; British Thoracic Society. Emergency oxygen use in adult patients: Concise guidance. *Clin. Med.* **2011**, *11*, 372–375.
4. Corley, A.; Edwards, M.; Spooner, A.J.; Dunster, K.R.; Anstey, C.; Fraser, J.F. High flow oxygen via tracheostomy improves oxygenation in patients weaning from mechanical ventilation: A randomized cross-over study. *Intensive Care Med.* **2017**, *43*, 465–467.
5. Van Kaam, A.H.; Lachmann, R.A.; Herting, E.; De Jaegere, A.; van Iwaarden, F.; Noorduyn, L.A.; Kok, J.H.; Haitsma, J.J.; Lachmann, B. Reducing atelectasis attenuates bacterial growth and translocation in experimental pneumonia. *Am. J. Respir. Crit. Care Med.* **2004**, *169*, 1046–1053.
6. Manzano, F.; Fernández-Mondéjar, E.; Colmenero, M.; Poyatos, M.E.; Rivera, R.; Machado, J.; Catalán, I.; Artigas, A. Positive-end expiratory pressure reduces incidence of ventilator-associated pneumonia in nonhypoxemic patients. *Crit. Care Med.* **2008**, *36*, 2225–2231.
7. Pelosi, P.; Gattinoni, B.L. Prone position in acute respiratory distress syndrome. *Eur. Respir. J.* **2002**, *20*, 1017–1028.
8. Mounier, R.; Adrie, C.; Français, A.; Garrouste-Orgeas, M.; Cheval, C.; Allaouchiche, B.; Jamali, S.; Dinh-Xuan, A.T.; Goldgran-Toledano, D.; Cohen, Y.; et al. Study of prone positioning to reduce ventilator-associated pneumonia in hypoxaemic patients. *Eur. Respir. J.* **2010**, *35*, 795–804.
9. Suzuki, S.; Li, G.; Wilson, G.; Malinchoc, M.; Gajic, O. Current oxygen management in mechanically ventilated patients: A prospective observational cohort study. *J. Crit. Care* **2013**, *28*, 647–654.
10. Rachmale, S.; Li, G.; Wilson, G.; Malinchoc, M.; Gajic, O. Practice of Excessive FIO_2_ and Effect on Pulmonary Outcomes in Mechanically Ventilated Patients with Acute Lung Injury. *Respir. Care* **2012**, *57*, 1887–1893.
11. Meyhoff, C.S.; Wetterslev, J.; Jorgensen, L.N.; Henneberg, S.W.; Høgdall, C.; Lundvall, L.; Svendsen, P.; Mollerup, H.; Lunn, T.H.; Simonsen, I.; et al. PROXI Trial Group. Effect of high perioperative oxygen fraction on surgical site infection and pulmonary complications after abdominal surgery: The PROXI randomized clinical trial. *JAMA* **2009**, *302*, 1543–1550.
12. Stub, D.; Smith, K.; Bernard, S.; Nehme, Z.; Stephenson, M.; Bray, J.E.; Cameron, P.; Barger, B.; Ellims, A.H.; Taylor, A.J.; et al. AVOID Investigators. Air versus oxygen in ST-segment-elevation myocardial infarction. *Circulation* **2015**, *131*, 2143–2150.
13. Kilgannon, J.H.; Jones, A.E.; Shapiro, N.I. Association between arterial hyperoxia following resuscitation from cardiac arrest and in-hospital mortality. *JAMA* **2010**, *303*, 2165–2171.
14. Girardis, M.; Busani, S.; Damiani, E.; Donati, A.; Rinaldi, L.; Marudi, A.; Morelli, A.; Antonelli, M.; Singer, M. Effect of Conservative vs. Conventional Oxygen Therapy on Mortality among Patients in an Intensive Care Unit the Oxygen-ICU Randomized Clinical Trial. *JAMA* **2016**, *316*, 1583–1589.
15. Mehta, A.; Bhagat, R. Preventing Ventilator-Associated Infections. *Clin. Chest Med.* **2016**, *37*, 683–692.
16. Helmerhorst, H.J.; Arts, D.L.; Schultz, M.J.; van der Voort, P.H.J.; Abu-Hanna, A.; de Jonge, E.; van Westerloo, D.J. Metrics of Arterial Hyperoxia and Associated Outcomes in Critical Care. *Crit. Care Med.* **2017**, *45*, 187–195.
17. Altemeier, W.A.; Altemeier, W.A. Hyperoxia in the intensive care unit: Why more is not always better. *Curr. Opin. Crit. Care* **2007**, *13*, 73–78.
18. Sinclair, S.E.; Altemeier, W.A.; Matute-Bello, G.; Chi, E.Y. Augmented lung injury due to interaction between hyperoxia and mechanical ventilation. *Crit. Care Med.* **2004**, *32*, 2496–2501.
19. Kallet, R.H.; Matthay, M.A. Hyperoxic acute lung injury. *Respir. Care.* **2013**, *58*, 123–141.
20. Dantzker, D.R.; Wagner, P.D.; West, J.B. Proceedings: Instability of poorly ventilated lung units during oxygen breathing. *J. Physiol.* **1974**, *242*, 72P.
21. Hafner, S.; Beloncle, F.; Koch, A.; Radermacher, P.; Asfar, P. Hyperoxia in intensive care, emergency, and peri-operative medicine: Dr. Jekyll or Mr. Hyde? A 2015 update. *Ann. Intensive Care* **2015**, *5*, 42.
22. Forel, J.M.; Voillet, F.; Pulina, D.; Gacouin, A.; Perrin, G.; Barrau, K.; Jaber, S.; Arnal, J.; Fathallah, M.; Auquier, P.; et al. Ventilator-associated pneumonia and ICU mortality in severe ARDS patients ventilated according to a lung-protective strategy. *Crit. Care* **2012**, *16*, R65.
23. Rello, J.; Lisboa, T.; Koulenti, D. Respiratory infections in patients undergoing mechanical ventilation. *Lancet Respir. Med.* **2014**, *2*, 764–774.
24. O’Reilly, P.J.; Hickman-Davis, J.M.; Davis, I.C.; Matalon, S. Hyperoxia impairs antibacterial function of macrophages through effects on actin. *Am. J. Respir. Cell. Mol. Biol.* **2003**, *28*, 443–450.
25. Patel, V.S.; Sitapara, R.A.; Gore, A.; Phan, B.; Sharma, L.; Sampat, V.; Li, J.H.; Yang, H.; Chavan, S.S.; Wang, H.; et al. High Mobility Group Box-1 mediates hyperoxia-induced impairment of *Pseudomonas aeruginosa* clearance and inflammatory lung injury in mice. *Am. J. Respir. Cell. Mol. Biol.* **2013**, *48*, 280–287.

##                                        

 

SESSION VII. “NEW WAYS TO TREAT SEVERE INFECTIONS”

 

## Nebulized Antibiotic Therapy

ChastreJeanService de Réanimation Médicale, ICAN, Institute of Cardiometabolism and Nutrition, Groupe Hospitalier Pitié, Salpêtrière, Assistance Publique, Hôpitaux de Paris, Université Pierre et Marie Curie, Paris 6, Paris, France; jean.chastre@aphp.fr

**Abstract:** Hospital-acquired pneumonia (HAP), including ventilator-associated pneumonia (VAP), is still a common ICU infection, with a high attributable morbidity and mortality. With current standard-of-care therapy, clinical success rates are often less than 60%, related to the challenges of antibiotic therapy in critically ill patients, including alterations in pharmacokinetics (PK) and pharmacodynamics (PD) with large increases in antibiotics volume of distribution and augmented renal clearance, relative low penetration of most antibiotics into the lung tissue and the frequency of difficult-to-treat or highly resistant pathogens in that setting [1].

Aerosol antibiotic administration offers the theoretical advantages of achieving high drug concentrations at the infection site, well above the minimal inhibitory concentration (MIC) of most causative microorganisms, and low systemic absorption, thereby avoiding toxicity, particularly the renal toxicity of aminoglycosides or colistin. The poor development of this potentially advantageous technique in patients on mechanical ventilation (MV) is partly due to the fact that, during MV, high amounts of the particles dispersed by conventional nebulizers deposit in the ventilatory circuits and the tracheobronchial tree, therefore not reaching the distal lung and hence less drug is available in the alveolar compartment. Current non-standardized clinical practice, the difficulties of implementing optimal nebulization technique and lack of robust clinical data considerably limits widespread adoption.

The optimal mass median aerodynamic diameter allowing distal lung deposition ranges between 0.5 and 3 μm. Particles larger than 5 μm massively deposit in the ventilator circuit and large airways, and very small droplets <1 μm are mostly exhaled in the expiratory limb of the ventilator circuit. Jet nebulizers appear less efficient than ultrasonic and vibrating-mesh nebulizers for antibiotic delivery. Nebulizers operating continuously during both insufflation and expiration should be placed in the inspiratory limb, 15–40 cm upstream of the Y-piece. Humidified gas increases the size of aerosol particles through hygroscopic water absorption; therefore, active heated humidifier should be switched off during nebulization. Ventilator settings that favor nebulization efficiency include low respiratory frequency, low inspiratory flow and increased inspiratory time. Tolerance of such specific ventilator settings in awaked patients may be poor and the benefit/risk ratio of temporary sedation during nebulization should be discussed on a case by case basis. Those practical constraints represent an important hurdle for performing clinical trials and ultimately for large scale feasibility of inhaled antibiotic therapy in daily clinical routine [2].

The most frequently described events secondary to inhaled antibiotics are cough and a disagreeable taste, which are minor and transient. Bronchospasm is a more severe, possibly life-threatening, but less common side effect that has been reported in patients receiving nebulized antibiotic, especially when the intravenous formulation was used. The prolonged use of broad-spectrum antibiotics is also known to lead to emergence of multi-resistant strains, and nebulization is not an exception to the rule. Thus, as for every antibiotic strategy, aerosolization must be managed prudently, particularly concerning treatment duration, that should be kept as short as possible [2].

Several studies have confirmed in animal models and also in patients with VAP that aerosol administration permits to reach very high antibiotic concentrations into the proximal airways respiratory secretions, as well as the epithelial lining fluid [3]. However, although some studies based on relatively low numbers of patients could demonstrate a positive result, none of them enables to draw definitive conclusion on relevant clinical endpoints, such as mortality and/or duration of MV, even when their results were meta-analysed [4,5]. Results of two large, double blind, placebo-controlled randomized trials were recently made available. In the first one, the investigators compared standard of care in each arm plus 300 mg amikacin/120 mg fosfomycin or placebo (saline), delivered by aerosol twice daily for 10 days via an efficient mesh-vibrating nebulizer [6]. A total of 143 patients with gram-negative bacterial VAP were studied. Comparison of CPIS change from baseline between treatment groups was not different, as well as the secondary hierarchical end point of no mortality and clinical cure at day 14.

The second study was the Bayer Inhaled study, a multinational, randomized, placebo-controlled, double-blind, multi-center study trial which investigated the clinical efficacy and safety of adjunctive “Amikacin Inhale” over standard of care IV antibiotics and aerosolized placebo for the treatment of Gram-negative pneumonia in adult ICU ventilated [7]. The primary outcome measure was survival at day 30. Secondary outcome measures included pneumonia-related mortality through to day 30, early clinical response up to day 10, number of days on MV, and number of ICU days up to day 30. 725 eligible patients were randomized into 2 arms. Patients in the first arm received 400 mgs of a specially formulated Amikacin Inhalation Solution every 12 h for 10 days administered using the Synchronized Inhalation System, a mesh-vibrating nebulizer synchronized with inspiration. Patients in the comparator arm received aerosolized placebo every 12 h for 10 days, also administered using the using the Synchronized Inhalation System. Both groups received standard of care IV antibiotics following American Thoracic Society (ATS) guidelines or local guidelines. The primary endpoint (overall mortality at day 28), as well as secondary endpoints were similar in both treatment arms, and therefore the study failed to demonstrate superiority of adjunctive aerosolized amikacin versus standard of care.

In summary, although promising and based on a strong PK/PD rationale, antibiotic aerosolization to treat HAP/VAP has not been demonstrated to improve clinically relevant outcomes. At present, antibiotic aerosolization can only be recommended to treat patients with HAP/VAP caused by multidrug-resistant microorganisms only susceptible to antibiotics with evidence of limited efficacy when given by the intravenous route: i.e., aminoglycosides and colistin [1]. Future investigation should focus on continuous improvement of nebulization practice and technique in these very specific populations of patients infected with extensively-drug resistant pathogens. A paradigm change might occur in the future with the development of inhaled anti-infective nanoparticle, antibodies or phage therapy.

**References**

1. Kalil, A.C.; Metersky, M.L.; Klompas, M.; Muscedere, J.; Sweeney, D.A.; Palmer, L.B.; Napolitano, L.M.; O’Grady, N.P.; Bartlett, J.G.; Carratala, J.; et al. Management of adults with hospital-acquired and ventilator-associated pneumonia: 2016 Clinical Practice Guidelines by the Infectious Diseases Society of America and the American Thoracic Society. *Clin. Infect. Dis.* **2016**, *63*, e61–e111.
2. Ehrmann, S.; Chastre, J.; Diot, P.; Lu, Q. Nebulized antibiotics in mechanically ventilated patients: A challenge for translational research from technology to clinical care. *Ann. Intensive Care* **2017**, *7*, 78.
3. Niederman, M.S.; Chastre, J.; Corkery, K.; Fink, J.B.; Luyt, C.E.; García, M.S. BAY41–6551 achieves bactericidal tracheal aspirate amikacin concentrations in mechanically ventilated patients with Gram-negative pneumonia. *Intensive Care Med.* **2012**, *38*, 263–271.
4. Solé-Lleonart, C.; Rouby, J.J.; Blot, S.; Poulakou, G.; Chastre, J.; Palmer, L.B.; Bassetti, M.; Luyt, C.E.; Pereira, J.M.; Riera, J.; et al. Nebulization of antiinfective agents in invasively mechanically ventilated adults: A systematic review and meta-analysis. *Anesthesiology* **2017**, *126*, 890–908.
5. Russell, C.J.; Shiroishi, M.S.; Siantz, E.; Wu, B.W.; Patino, C.M. The use of inhaled antibiotic therapy in the treatment of ventilator-associated pneumonia and tracheobronchitis: a systematic review. *BMC Pulm. Med.* **2016**, *16*, 40.
6. Kollef, M.H.; Ricard, J.D.; Roux, D.; Francois, B.; Ischaki, E.; Rozgonyi, Z.; Boulain, T.; Ivanyi, Z.; János, G.; Garot, D.; et al. Randomized trial of the Amikacin Fosfomycin Inhalation System for the adjunctive therapy of Gram-negative ventilator-associated pneumonia: IASIS Trial. *Chest* **2017**, *151*, 1239–1246.
7. Phase III Study Program with Amikacin Inhale in Addition to Standard of Care in Intubated and Mechanically Ventilated Patients with Gram-Negative Pneumonia Does Not Meet Primary Endpoint of Superiority. Available online: http://press.bayer.com/baynews/baynews.nsf/id/Phase-III-study-program-Amikacin-Inhale-addition-standard-intubated-mechanically-ventilated-patients?OpenDocument&sessionID=1515601612 (accessed on 24 November 2017).

## Empirical Antifungal Therapy in the ICU: Time to Revisit?

CalandraThierryProfessor of Medicine, Head, Infectious Diseases Service, Centre Hospitalier Universitaire Vaudois, CH-1011 Lausanne, Switzerland; Thierry.Calandra@chuv.ch

**Abstract:** Invasive mycoses are life-threatening opportunistic infections and recently emerged as a major cause of morbidity and mortality in the intensive care unit (ICU) and in surgical and critically ill immunocompromised patients. Candida and Aspergillus species are the most frequent fungal pathogens in the critical care setting and account for 80 to 90% of all invasive fungal infections [1–3]. Appropriateness and timing of initiation of antifungal therapy influence the outcome of invasive fungal infections (IFI). Several studies have shown that inappropriate therapy or delays in the introduction of appropriate antifungals increased mortality in patients with IFI [2]. This suggests a role for preventive antifungal therapy such as prophylaxis, preemptive or empirical therapy in the management of IFI. However, criteria for initiating preventive antifungal strategies remain ill-defined and should be balanced against the potential risks of toxicity, selection of resistance and treatment costs [2,3]. Up until now, investigators conducted less than twenty-five antifungal preventive studies in ICU patients [4,5].

Prophylaxis is the best-studied approach warranted in selected patients who are at highest risk (i.e., a rate of IFI greater than 5%). Yet, preemptive and empirical therapy are the favored preventive antifungal therapies. Preemptive therapy, defined as the initiation of therapy based on biomarkers of fungal infections, is gaining interest, but one is lacking studies to better define patients who may benefit from this approach in the ICU. The risk is that widespread use of antifungal agents given as prophylaxis or preemptive therapy may affect fungal ecology and increase resistance against antifungal agents.

Only two studies examined the impact of empirical therapy in ICU patients [6,7]. In a double-blind study conducted from 1995 to 2000 that included 270 ICU adult patients with persistent fever despite broad-spectrum antibiotics, intravenous fluconazole (800 mg per day) and placebo resulted in similar rates of successful outcome (36% versus 38%, *p* = 0.78) [6]. A composite index of primary outcome that included four items (resolution of fever, absence of invasive fungal infection, no discontinuation of therapy because of toxicity and no need for non-study systemic antifungal medication) defined success as the achievement of all four items. The lack of fever resolution was the main reason for failure in more than 50% of the patients. Documented IFI were present in only 17 patients, 6 (5%) in the fluconazole group and 11 (9%) in the placebo. In a multicenter, double-blind, placebo-controlled study conducted between 2012 and 2015 in 260 nonneutropenic and nontransplanted critically ill patients with ICU-acquired sepsis, micafungin did not increase fungal infection-free survival at day 28 [7]. This primary endpoint occurred in 68.0% of the micafungin-treated patients and in 60.2% of the placebo-treated patients (hazard ratio: 1.35, 95% CI, 0.87–2.08). However, micafungin reduced the number of new IFI (3% versus 12%, *p* = 0.008).

According to the 2016 guidelines of the Infectious Diseases Society of America (IDSA), the empiric antifungal therapy in nonneutropenic patients in the ICU should be considered in critically ill non-neutropenic patients with risk factors for invasive candidiasis and no other known cause of fever [8]. It should be guided by clinical assessment of risk factors, surrogate markers for invasive candidiasis, and/or culture data from non-sterile sites (strong recommendation; moderate-quality evidence). Empiric antifungal therapy should be started as soon as possible in patients who have risk factors and who have clinical signs of septic shock (strong recommendation; moderate-quality evidence). Preferred empiric therapy is an echinocandin (strong recommendation; moderate-quality evidence). Alternative therapies are fluconazole (strong recommendation; moderate-quality evidence) or lipid formulations of amphotericin B (strong recommendation; low-quality evidence). Additional clinical studies are needed to better define the place of empirical therapy among preventive antifungal strategies in the ICU.

**References**

1. Delaloye, J.; Calandra, T. Invasive candidiasis as a cause of sepsis in the critically ill patient. *Virulence* **2014**, *1*, 161–169.
2. Pascale. G.; Tumbarello, M. Fungal Infections in the ICU: Advances in treatment and diagnosis. *Curr. Opin. Crit. Care* **2015**, *5*, 421–429.
3. Calandra, T.; Roberts, J.A.; Antonelli, M.; Bassetti, M.; Vincent, J.L. Diagnosis and management of invasive candidiasis in the ICU: An updated approach to an old enemy. *Crit. Care* **2016**, *20*, 125.
4. Cortegiani, A.; Russotto, V.; Maggiore, A.; Attanasio, M.; Naro, A.R.; Raineri, S.M.; Giarratano, A. Antifungal agents for preventing fungal infections in non-neutropenic critically ill patients. *Cochrane Database Syst. Rev.* **2016**, *1*, CD004920.
5. Dupont, H.; Mahjoub, Y.; Chouaki, T.; Lorne, E.; Zogheib, E. Antifungal Prevention of Systemic Candidiasis in Immunocompetent ICU Adults: Systematic Review and MetaAnalysis of Clinical Trials. *Crit. Care Med.* **2017**, *45*, 1937–1945.
6. Schuster, M.G.; Edwards, J.E., Jr.; Sobel, J.D.; Darouiche, R.O.; Karchmer, A.W.; Hadley, S.; Slotman, G.; Panzer, H.; Biswas, P.; Rex, J.H. Empirical Fluconazole versus Placebo for Intensive Care Unit Patients. A Randomized Trial. *Ann. Intern. Med.* **2008**, *149*, 83–90.
7. Timsit, J.F.; Azoulav, E.; Schwebel, C.; Charles, P.E.; Cornet, M.; Souweine, B.; Klouche, K.; Jaber, S.; Trouillet, J.L.; Bruneel, F.; et al. Empirical micafungin treatment and survival without invasive fungal infection in adults with ICU-acquired sepsis, Candida colonization and multiple organ failure: The EMPIRICUS randomized clinical trial. *JAMA* **2016**, *316*, 1555–1564.
8. Pappas, P.; Kauffman, C.A.; Andes, D.R.; Clancy, C.J.; Marr, K.A.; Ostrosky-Zeichner, L.; Reboli, A.C.; Schuster, M.G.; Vazquez, J.A.; Walsh, T.J. Clinical Practice Guideline for the Management of Candidiasis: 2016 Update by the Infectious Diseases Society of America. *Clin. Infect. Dis.* **2016**, *62*, e1–e50.

## Carbon Monoxide: Mechanisms to control inflammation

ChoiAugustine M. K.Weill Cornell Medicine, New York, USA

**Abstract:** The era of gaseous molecules officially began with reports of endothelium-dependent vasorelaxation in 1980, which led to the unequivocal identification of EDRF as nitric oxide (NO). At that time, the concept that a substance which was not a peptide, protein, lipid mediator, or nucleic acid but endogenously derived gas such as NO could exert vital biological functions truly represented a paradigm shift in the life sciences. Since the formal discovery of NO, the extraordinary advances in the biology and chemistry of NO have improved our understanding not only of physiological processes but also of the pathogenesis of human diseases. The remarkable translational application of the basic knowledge of NO to human diseases has opened new avenues of biomedical research and drug discovery.

Ironically, we have known for a longer time, some two decades prior to the discovery of NO, that living organisms could produce another endogenous gaseous molecule: carbon monoxide (CO). CO, a diatomic gas, occurs in nature as a product of oxidation of organic matter. This invisible, chemically inert, colorless and odorless gas is commonly viewed as a poison, because at high concentrations it can produce deleterious physiologic effects and, with prolonged exposure, ultimately death. The well-known toxic effects of CO involve the avid binding of CO to hemoglobin with an affinity 240 times that of oxygen, resulting in tissue hypoxia due to displacement of oxygen from hemoglobin. In 1968, the precise biochemical mechanism for the endogenous production of CO was first described. CO originates from the breakdown of heme by the heme oxygenase (HO) enzymes. In humans, endogenous CO production arises principally from HO-catalyzed heme degradation from the turnover of hemoglobin and other cellular hemoproteins, with minor contributions from other metabolic pathways. HO, which exists as two major isoforms (HO-1, HO-2), catalyzes the first and rate-limiting step in the oxidative degradation of heme to ferrous iron, biliverdin-IX, and CO. In the past 2 decades, the interest in HO isozymes has shifted from their well-defined metabolic function of heme catabolism and erythrocyte turnover, to critical physiological function as a cytoprotective mechanism in numerous models of cellular stress and organ pathology. Although HO-derived CO production represents the major intracellular pathway that generates endogenous CO, it was largely considered metabolic waste by the scientific community during the first 25 years following the discovery of HO. Our laboratory in 2000 (*Nature Medicine*) was the first to show that low dose CO can exert cytoprotective effects in preclinical models of lung inflammation and injury. Our study at that time provoked new interest in the field of gaseous molecules, but also generated considerable controversy. We introduced a novel paradigm that a toxic gas such as CO which can lead to deleterious physiologic dysfunction *via* binding to hemoglobin and even death when administered at high doses, could act as a cytoprotective agent when applied at low dose. Many laboratories including our own have since demonstrated that CO conferred cytoprotection *via* potent anti-inflammatory, anti-apoptotic and anti-proliferative effects. The PI and the project leaders have collectively demonstrated the cytoprotective effects of low dose CO in various rodent models of lung injury and inflammation including hyperoxia, endotoxemia, bleomycin-induced lung fibrosis, ventilator-induced lung injury, ischemia-reperfusion induced lung injury, organ transplantation including lung and non-pulmonary tissue injury including models of myocardial ischemia, ileus, hemorrhagic shock and vascular injury. Importantly, other laboratories have also reported cytoprotective effects of inhaled CO in various models of tissue injury. Furthermore, these responses were regulated by cell- or tissue-specific, and stimuli- or stress-specific signaling. An improved understanding of the mechanisms by which CO mediates its cytoprotective effects would greatly enhance the potential to translating the use of this gas for human disease, and provide insight into additional therapeutic targets. We will review the preclinical studies of inhaled CO in experimental models of tissue inflammation and provide update on Phase I/II clinical trial of inhaled CO in lung diseases.

**Suggested Reading**

1. Otterbein, L.E.; Bach, F.H.; Alam, J.; Soares, M.; Tao Lu, H.; Wysk, M.; Davis, R.J.; Flavell, R.A.; Choi, A.M. Carbon monoxide has anti-inflammatory effects involving the mitogen-activated protein kinase pathway. *Nat. Med.* **2000**, *6*, 422–428.
2. Otterbein, L.E.; Zuckerbraun, B.S.; Haga, M.; Liu, F.; Song, R.; Usheva, A.; Stachulak, C.; Bodyak, N.; Smith, R.N.; Csizmadia, E.; at al. Carbon monoxide suppresses arteriosclerotic lesions associated with chronic graft rejection and with balloon injury. *Nat. Med.* **2003**, *9*, 183–190.
3. Nakahira, K.; Choi, A.M. Carbon monoxide in the treatment of sepsis. *Am. J. Physiol. Lung Cell. Mol. Physiol.* **2015**, *309*, L1387–L1393, doi:10.1152/ajplung.00311.2015.

## Anticoagulants Nebulization in Severe Pulmonary Infections

SchultzMarcus J.Academic Medical Center, Amsterdam, The Netherlands: Laboratory of Experimental Intensive Care and Anesthesiology (LEICA) and Mahidol University, Bangkok, Thailand: Mahidol–Oxford Research Unit (MORU)

**Introduction**

Coagulation and inflammation use extensive reciprocal activation and amplification in the response against systemic but also local insults [1]. Pneumonia is characterized by dysregulated coagulation and inflammation within the pulmonary compartment [2,3]. Clinical trials of systemically administered anticoagulant agents in patients with pneumonia so far failed to show beneficial effects of anticoagulant [4]. In these trials, dosages of anticoagulant agents could have been too low, and systemic bleeding may have offset any possible positive effect. Nebulization of anticoagulant agents may improve local biological availability, and could limit the systemic anticoagulant effects.

**Methods**

Recently, we reported on a systematic search of the medical literature to identify preclinical investigations and clinical trials of nebulized anticoagulant agents in the setting of lung injury [5]. For that search we used various terms referring to aspects of the condition of interest (i.e., pneumonia, ARDS, lung trauma), the intervention of interest (i.e., intratracheal, nebulized, vaporized, or aerosolized) and various anticoagulant agents, though limited to those that are commercially available or have been tested in clinical trials in critically ill patients (i.e., heparin, danaparoid, activated protein C, antithrombin and tissue factor pathway inhibitor). We here focus on findings regarding nebulized anticoagulant agents in the setting of pneumonia.

**Search Results**

The systematic search identified only five articles reporting on preclinical studies using pneumonia models in mice [6] and rats [7]; the search failed to find articles reporting on trials in the setting of pneumonia in humans. Quality of outcome of the systemic review was limited due to the heterogeneity of included articles of animal studies, as the five preclinical studies varied greatly in, e.g., models fro pneumonia, technique of administration of anticoagulant agents, and used endpoints.

**The Preclinical Studies Identified by the Search**

Ader et al. examined the effects of intratracheal instillation of heparin in a model of *Legionella pneumophila* pneumonia in mice [6]. For this, mice received heparin intratracheally, co–instilled with a virulent isolate of *L. pneumophila.* Hofstra et al. examined the effect of nebulized heparin, danaparoid, activated protein C, or antithrombin on pulmonary coagulopathy and inflammation in a model of *Streptococcus pneumoniae* pneumonia in rats [7]. For this, rats were challenged intratracheally with *S. pneumoniae*, inducing pneumonia, and received treatment before and after the bacterial challenge. Cornet et al. examined the effect of nebulization of the same anticoagulant agents on pulmonary coagulopathy and inflammation in a model of *Pseudomonas aeruginosa* pneumonia in rats [8]. For this they used the same set–up as in the study by Hofstra et al. [7], but animals were infected with *Pseudomonas aeruginosa* instead of *Streptococcus pneumoniae.* Van den Boogaard et al. examined the effect of nebulized tissue factor pathway inhibitor in two models of acute lung injury in rats [9]. For this, they intratracheally instilled *Pseudomonas aeruginosa*, causing direct lung injury, or gave an intravenous injection of *Escherichia coli* lipopolysaccharide, causing indirect lung injury. Tissue factor pathway inhibitor was administered by nebulization before and after induction of pneumonia or endotoxemia. Finally, Chimenti et al. examined the effects of nebulized heparin in a model of acute lung injury in rats (10). For this, they intratracheally instilled lipopolysaccharide. Heparin was nebulized at various and different timepoints.

**Main Findings**

In the diverse preclinical models of pneumonia the anticoagulant agents heparin, danaparoid, activated protein C, antithrombin and tissue factor pathway inhibitor were capable of attenuating pulmonary coagulopathy [6–10]. However, only heparin (6, 10), antithrombin [7] and tissue factor pathway inhibitor [9] were capable of dampening pulmonary inflammation. Of note, danaparoid and activated protein C also had systemic effects [7–9], and while bacterial clearance from the lung was found with all nebulized anticoagulants, a remarkable trend towards increased bacteremia was found with heparin and danaparoid [8].

**Discussion**

The reasons for not finding an inhibitory effect on inflammation in all preclinical studies remain unexplained. Nebulized anticoagulants have not yet been tested in the setting of pneumonia in humans.

**Conclusions**

Nebulized anticoagulant agents exert local anticoagulant effects in the lungs, an effect found with all tested agents and in all models. Nebulization of anticoagulant agents also results in local anti–inflammatory effects, though this was not find with all agents tested, and was not confirmed in all models. Nebulized anticoagulants can affect systemic coagulation.

**Recommendations**

Future clinical studies need to focus on the way to nebulize anticoagulant agents, efficient and safe dosages, and the side–effects.

**References**

1. Levi, M.; van der Poll, T.; Buller, H.R. Bidirectional relation between inflammation and coagulation. *Circulation* **2004**, *109*, 2698–2704.
2. Gunther, A.; Mosavi, P.; Heinemann, S.; Ruppert, C.; Muth, H.; Markart, P.; Grimminger, F.; Walmrath, D.; Temmesfeld-Wollbrück, B.; Seeger, W. Alveolar fibrin formation caused by enhanced procoagulant and depressed fibrinolytic capacities in severe pneumonia. Comparison with the acute respiratory distress syndrome. *Am. J. Respir. Crit. Care Med.* **2000**, *161*, 454–462.
3. Schultz, M.J.; Millo, J.; Levi, M.; Hack, C.E.; Weverling, G.J.; Garrard, C.S.; van der Poll, T. Local activation of coagulation and inhibition of fibrinolysis in the lung during ventilator associated pneumonia. *Thorax* **2004**, *59*, 130–135.
4. Schultz, M.J.; Haitsma, J.J.; Zhang, H.; Slutsky, A.S. Pulmonary coagulopathy as a new target in therapeutic studies of acute lung injury or pneumonia—A review. *Crit. Care Med.* **2006**, *34*, 871–877.
5. Juschten, J.; Tuinman, P.R.; Juffermans, N.P.; Dixon, B.; Levi, M.; Schultz, M.J. Nebulized Anticoagulants in Lung Injury in Critically Ill Patients—An Updated Systematic Review of Preclinical and Clinical Studies. *Ann. Trans. Med.* **2018**, in press.
6. Ader, F.; Le Berre, R.; Fackeure, R.; Raze, D.; Menozzi, F.D.; Viget, N.; Faure, K.; Kipnis, E.; Guery, B.; Jarraud, S.; et al. In vivo effect of adhesion inhibitor heparin on *Legionella pneumophila* pathogenesis in a murine pneumonia model. *Intensive Care Med.* **2008**, *34*, 1511–1519.
7. Hofstra, J.J.; Cornet, A.D.; de Rooy, B.F.; Vlaar, A.P.; van der Poll, T.; Levi, M.; Zaat, S.A.J.; Schultz, M.J. Nebulized antithrombin limits bacterial outgrowth and lung injury in Streptococcus pneumoniae pneumonia in rats. *Crit. Care* **2009**, *13*, R145.
8. Cornet, A.D.; Hofstra, J.J.; Vlaar, A.P.; van den Boogaard, F.E.; Roelofs, J.J.; van der Poll, T.; Levi, M.; Groeneveld, A.B.J.; Schultz, M.J. Nebulized anticoagulants limit coagulopathy but not inflammation in pseudomonas aeruginosa-induced pneumonia in rats. *Shock* **2011**, *36*, 417–423.
9. Van den Boogaard, F.E.; Hofstra, J.J.; Brands, X.; Levi, M.M.; Roelofs, J.J.; Zaat, S.A.; van't Veer, C.; van der Poll, T.; Schultz, M.J. Nebulized Recombinant Human Tissue Factor Pathway Inhibitor Attenuates Coagulation and Exerts Modest Anti-inflammatory Effects in Rat Models of Lung Injury. *J. Aerosol. Med. Pulm. Drug Deliv.* **2017**, *30*, 91–99.
10. Artigas, A. Nebulized Heparin Attenuates Pulmonary Coagulopathy and Inflammation through Alveolar Macrophages in a Rat Model of Acute Lung Injury. *Thromb. Haemost.* **2017**, *117*, 2125–2134.

##                                        

 

SESSION IX. “NEW DIRECTIONS IN PNEUMONIA MANAGEMENT”

 

## International Guidelines for the Management of Hospital-Acquired Pneumonia (HAP) and Ventilator-Associated Pneumonia (VAP). An ERS/ESCIM/ESCMID/ALAT Guideline

TorresA.NiedermanM.S.ChastreJ.EwigS.Fernandez-VandellosP.HanbergerH.KollefM.Li BassiG.LunaC.M.Martin-LoechesI.Artur PaivaJ.ReadR.C.RigauD.TimsitJ.F.WelteT.WunderinkR.International ERS/ESICM/ESCMID/ALAT guidelines for the management of hospital-acquired pneumonia (HAP)/ventilator-associated pneumonia (VAP): guidelines for the management of HAP/VAP of the European Respiratory Society (ERS), European Society of Intensive Care Medicine (ESICM), European Society of Clinical Microbiology and Infectious Diseases (ESCMID) and Asociación Latinoamericana del Tórax (ALAT). Eur Respir J 2017; 0: 1700582 Accessible at: https://doi.org/10.1183/13993003.00582–2017

**Abstract:** The most recent European guidelines and task force reports on hospital-acquired (HAP) and ventilator-acquired pneumonia (VAP) were published almost 10 years ago. Since then, further randomized clinical trials on HAP and VAP management have been conducted and changed old paradigms. The European Respiratory Society (ERS) launched a project to develop new international guidelines for HAP and VAP. Other European societies including the European Society of Intensive Care Medicine (ESICM) and the European Society of Clinical Microbiology and Infectious Diseases (ESCMID), were invited to participate, and appointed their representatives. The Latin American Society of Thoracic Diseases (ALAT) was also invited. A total of 15 experts and two methodologists made up the panel. Three experts from the US were also invited (Michael Niederman, Marin Kollef, and Richard Wunderink). Applying the GRADE methodology, the panel selected seven PICO questions which generated a series of recommendations for HAP/VAP diagnosis, treatment and prevention. The document also includes a supplement with practical advice for specific antibiotic management and recommendations for future research. The seven PICO questions and their corresponding recommendations are summarized below.

The first question was about the use of distal quantitative samples instead of quantitative proximal samples in patients with suspected VAP. The panel recommended the obtention of lower respiratory respiratory samples preferentially quantitative and distal in order to reduce antibiotic exposure and to narrow the initial antibiotic exposure

The second question was about what “low-risk patients” with suspected HAP or VAP could receive safely narrow-spectrum antibiotic treatment. The panel recommended the use of narrow-spectrum in patients without septic shock, no other risk factors for MDR pathogens and those who are not in hospitals with a high background rate of resistant pathogens (>25%)

The use of monotherapy versus combination of antibiotics was the third question. The panel recommended the use of combination therapy in high risk HAP/VAP patients to cover Gram negative bacteria and meticilin resistant S.aureus (MRSA) in those patiens at risk (septic shock, hospital settings with high rates of multidrug-resistant microorganisms (MDR) (25%), previous antibiotic use, recent prolonged hospital stay (>5 days), and previous colonization with MDR pathogens.

The duration of antibiotic treatment (7–10 days versus 14 days) was the fourth question. The recommendation of the panel was to use 7–8 days of antibiotic treatment in patients with HAP or VAP with a good clinical response to therapy (except those with immunodeficiency, cystic fibrosis, empyema, lung abscess or necrotizing pneumonia). This recommendation also includes patients with nonfermenting Gram-negatives, *Acinetobacter*, and MRSA with good clinical response. Longer courses of antibiotics may be needed in patients with inappropriate initial empiric therapy, and should be individualized to the patients clinical response, to the presence of pan drug resistant microorganisms (PDR), MRSA and bacteremia, and to the serial measuremet of biomarkers when indicated.

Using bedside clinical assessment with or without serial detection of biomarkers to predict outcomes and clinical response was the question number 5. The panel believed that performing routine bedside clinical assessment represents good practice. Clinical evaluation included aspect and volume of tracheobronchial secretions, white blood cell count, oxygenation and calculation of one or more scores such as CPIS, ODIN, SOFA, SAPSII, and APACHE II.

The sixth question was about the use of procalcitonin (PCT) blood serial measurements yes or not to reduce the duration of antibiotic HAP/VAP treatment. According to the evidence the panel did not recommend the use of serial serum PCT level to reduce duration of antibiotic course in patients with HAP/VAP when the anticipated duration is 7–8 days. Serial biomarker determination could be used in patients with initially inappropriate antibiotic therapy, severely immunocompromised, highly antibiotic-resiatnt pathogens (*Pseudomonas aeruginosa*, carbapnem-resistant *Acinetobacter* spp., carbapenem-resistant Enterobacteriaceae, and when using second line antibiotic therapy such as colistin or ttgecycline)

The last question was about prevention of VAP using selective oropharyngeal decontamination (SOD: antibiotics or chlorhexidine) or non absorbable antibiotics in the oropharynx and intestinal tract along with intravenous antibiotics (SDD). The panel decided not to issue a recommendation on the use of SOD with clorhexidine in patients requiring mechanical ventilation until more safety data become available, due to the unclear balance between the potential reduction in pneumonia rate and potential increase in mortality. Finally it was recommended the use of SOD (antibiotics), but not SDD, in settings with low rates of antibiotic resistant bacteria (5% threshold) and low-antibiotic consumption (<1000 daily doses per 1000 admission days).

Figure 1 shows the algorithm recommendation for the initial empirical antibiotic treatment.

## Ventilator Associated Tracheobronchitis: When Not to Treat

KeaneSean^1^Martin-LoechesIgnacio^2^*1Anaesthesia Specialist Registrar, Department of Anaesthesia and Critical Care Medicine, St. James’s Hospital, James’s Street, Dublin, Ireland; sean.e.keane@gmail.com2Consultant Intensivist, Department of Anaesthesia and Critical Care Medicine, St. James’s Hospital, James’s Street, Dublin, Ireland*Correspondence: drmartinloeches@gmail.com

**Background**

Ventilator associated tracheobronchitis (VAT) is a common occurance in intubated mechanically ventilated patients. There is no gold standard definition of VAT, and this makes an accurate assessment of its prevalence difficult, though it is estimated to be between 0 and 15% in intubated mechanically ventilated patients. (Malacarne P, 2008; Bouza E, 2013; Martin-Loeches I, 2015). VAT has been defined differently in recent years. For the Centers for Disease Control (CDC) the definition of VAT in adult patients must meet the radiological criteria of absence of pneumonia in the X-ray and at least 2 of the following findings: fever (>38 °C), cough, new or increased production of sputum, rhonchi and wheezing, or bronchospasm. In addition, a positive culture of bronchial secretions obtained by endotracheal aspirate (ETA) or bronchoscopic technique should be positive (Garner JS, 1988). More recently, an updated definition has been more commonly used. Along with an absence of pulmonary infiltrates on chest radiographs, a diagnosis of VAT requires the presence of at least two of the following criteria: body temperature >38.5 or <36.5, leucocyte count >12,000 cells per uL or <4000 cells per uL, and purulent endotracheal aspirates (ETA) or bronchoalveolar lavage (BAL). In addition, the VAT must be microbiologically confirmed by the growth of a potentially pathogenic microorganism in the ETA of at least 10^5^ colony-forming units (CFU) per mL, or with a BAL of at least 10^4^ CFU per mL (Malacarne P, 2008; Nseir S, 2008; Martin-Loeches I, 2009; Martin Loeches I, 2015). It is important to note that the combination of clinical findings and microbiological confirmation leads to a diagnosis of VAT, and not when they occur independently of one another. Issues continue to exist in relation to the diagnosis of VAT, and it remains to be seen if an accepted gold standard will emerge in the near future.

**Body**

It is increasingly recognised that a continuum exists between bacterial colonization of the lower respiratory tract, VAT, and ventilator associated pneumonia (VAP) (Nseir S, 2002; Craven DE, 2013; Nseir S, 2016). Colonization refers to the microbiological growth of potentially pathogenic microorganisms in tracheobronchial ETA or BAL samples, without evidence of local or systemic infection. VAT and VAP share common clinical findings, and are differentiated by the presence or absence of pulmonary infiltrates on chest radiographs. It is recognised that computed tomography or lung ultrasound are more efficient than chest radiography in detecting pulmonary infiltrates (Mongodi S, 2016). While this makes it difficult to make a differential diagnosis between VAT and VAP, it is important to recognise that they are different entities, even if they are in continuum.

VAT has been shown to prolong the duration of invasive mechanical ventilation, increase the length of ICU stay, and predispose to subsequent episodes of VAP, without an increase in mortality when compared to intubated mechanically ventilated patients without VAT (Nseir S, 2002; Nseir S, 2005; Karvouniaris M, 2013; Martin-Loeches I, 2015). In contrast, VAP is a well recognised independent risk factor for increased mortality (Heyland DK, 1999), and has been shown to carry a higher mortality rate compared to those with VAT (Nseir S, 2005). Immune factors are likely to play a role in determining whether a patient with VAT goes on to develop VAP. In a study of mechanically ventilated patients with VAP, patients showed a relative depression of genes involved in calcium signaling pathways, the complement system and cyclic adenosine monophosphate (Martin-Loeches I, 2012). Patients with VAP exhibit increased inflammation and worse outcomes than those with VAT, and this may be related to an immunosuppressed state (Martin-Loeches I, 2015). As a result, is important to ensure measures are in place to reduce the risk of VAT progressing to VAP.

The role of antimicrobial therapy in VAT continues to be a matter for debate (Torres A, 2005; Torres A, 2009). In a recent international survey of 288 ICUs in 16 countries, approximately half of respondents believed patients with VAT should be treated with antimicrobials (Rodriguez A, 2014). Observational studies suggest appropriate antimicrobial therapy has a beneficial effect in the management of VAT, with shorter duration of invasive mechanical ventilation, reduced length of ICU stay, and lower incidence of subsequent VAP (Nseir S, 2002; Nseir S, 2014; Martin-Loeches 2015). Two small randomised trials evaluated the impact of antimicrobials on patients with VAT. In a study of 43 patients with VAT randomised to either aerosolized antimicrobials or placebo, Palmer and colleagues demonstrated reduced incidence of subsequent VAP in those receiving antimicrobials, though there was no difference in ICU length of stay or mortality between groups (Palmer LB, 2008). In contrast, when comparing systemic antimicrobials versus placebo for the treatment of VAT, Nseir and colleagues showed a survival benefit in those receiving antimicrobial therapy (Nseir S, 2008). When these observational and randomised papers were subjected to meta-analysis, it was concluded that antimicrobial therapy for VAT, versus placebo or no therapy, is associated with lower incidence of subsequent VAP and a greater number of ventilator free days (Agrafiotis M, 2010). There was no demonstratable difference in length of ICU stay or mortality.

**Conclusions**

So, when not to treat VAT? In conclusion, the answer is to never not treat VAT. Antimicrobial therapy for VAT reduces the risk of subsequent progression to VAP, which may improve survival. There may also be benefits in relation to duration of mechanical ventilation and length of ICU stay. Potential survival benefit may be demonstrated as a greater number of studies are published. It is essential to correctly diagnose VAT and institute appropriate antimicrobial therapy. Inaccurate diagnosis or inappropriate antimicrobial therapy, including the treatment of tracheobronchial colonisation, should be avoided. In time, newer technology may allow us to diagnose VAT and VAP in more sophisticated ways (Douglas IS, 2015; Bos LD, 2014), and determine which patients may be at an increased risk of progression from VAT to VAP based on immunological factors. This may enable targeted therapy towards those patients with VAT at the greatest risk of progression to VAP, with improved survival and a minimal excess use of antimicrobials. In the meantime, it is essential to accurately detect, diagnose and treat all patients with VAT.

**References**

1. Nseir, S.; Di Pompeo, C.; Pronnier, P.; Beague, S.; Onimus, T.; Saulnier, F.; Grandbastien, B.; Mathieu, D.; Delvallez-Roussel, M.; Durocher, A. Nosocomial tracheobronchitis in mechanically ventilated patients: Incidence, aetiology and outcome. *Eur. Respir. J.* **2002**, *20*, 1483–1489.
2. Nseir, S.; Di Pompeo, C.; Soubrier, S.; Cavestri, B.; Jozefowicz, E.; Saulnier, F.; Durocher, A. Impact of ventilator-associated pneumonia on outcome in patients with COPD. *Chest* **2005**, *128*, 1650–1656.
3. Craven, D.E.; Lei, Y.; Ruthazer, R.; Sarwar, A.; Hudcova, J. Incidence and outcomes of ventilator-associated tracheobronchitis and pneumonia. *Am. J. Med.* **2013**, *126*, 542–549.
4. Nseir, S.; Povoa, P.; Salluh, J.; Rodriguez, A.; Martin-Loeches, I. Is there a continuum between ventilator-associated tracheobronchitis and ventilator-associated pneumonia? *Intensive Care Med.* **2016**, *42*, 1190–1192.
5. Malacarne, P.; Langer, M.; Nascimben, E.; Moro, M.L.; Giudici, D.; Lampati, L.; Bertolini, G.; Italian Group for the Evaluation of Interventions in Intensive Care Medicine. Building a continuous multicenter infection surveillance system in the intensive care unit: Findings from the initial data set of 9493 patients from 71 Italian intensive care units. *Crit. Care Med.* **2008**, *36*, 1105–1113.
6. Bouza, E.; Granda, M.J.; Hortal, J.; Barrio, J.M.; Cercenado, E.; Muñoz, P. Pre-emptive broad-spectrum treatment for ventilator-associated pneumonia in high-risk patients. *Intensive Care Med.* **2013**, *39*, 1547–1555.
7. Martin-Loeches, I.; Povoa, P.; Rodríguez, A.; Curcio, D.; Suarez, D.; Mira, J.P.; Cordero, M.L.; Lepecq, R.; Girault, C.; Candeias, C.; et al. Incidence and prognosis of ventilator-associated tracheobronchitis (TAVeM): A multicentre, prospective, observational study. *Lancet Respir. Med.* **2015**, *3*, 859–868.
8. Garner, J.S.; Jarvis, W.R.; Emori, T.G.; Horan, T.C.; Hughes, J.M. CDC definitions for nosocomial infectons, 1988. *Am. J. Infect. Control* **1988**, *16*, 128–140
9. Mongodi, S.; Via, G.; Girard, M.; Rouquette, I.; Misset, B.; Braschi, A.; Mojoli, F.; Bouhemad, B. Lung ultrasound for early diagnosis of ventilator-associated pneumonia. *Chest* **2016**, *149*, 969–980.
10. Karvouniaris, M.; Makris, D.; Manoulakas, E.; Zygoulis, P.; Mantzarlis, K.; Triantaris, A.; Chatzi, M.; Zakynthinos, E. Ventilator-associated tracheobronchitis increases the length of intensive care unit stay. *Infect. Control Hosp. Epidemiol.* **2013**, *34*, 800–888.
11. Heyland, D.K.; Cook, D.J.; Griffith, L.; Keenan, S.P.; Brun-Buisson, C. The attributable morbidity and mortality of ventilator-associated pneumonia in the critically ill patient. *Am. J. Respir. Crit. Care Med.* **1999**, *159*, 1249–1256.
12. Martin-Loeches, I.; Papiol, E.; Almansa, R.; López-Campos, G.; Bermejo-Martin, J.F.; Rello, J. Intubated patients developing tracheobronchitis or pneumonia have distinctive complement system gene expression signatures in the pre-infection period: A pilot study. *Med. Intensiva* **2012**, *36*, 257–263.
13. Martín-Loeches, I.; Pobo, A. What is new in ventilador-associated tracheobronchitis? *Clin. Pulm. Med.* **2010**, *17*, 117–121.
14. Nseir, S.; Favory, R.; Jozefowicz, E.; Decamps, F.; Dewavrin, F.; Brunin, G.; Di Pompeo, C.; Mathieu, D.; Durocher, A.; VAT Study Group. Antimicrobial treatment for ventilator-associated tracheobronchitis: A randomized, controlled, multicenter study. *Crit. Care* **2008**, *12*, R62.
15. Craven, D.E.; Chroneou, A.; Zias, K.; Hijalmarson, K.I. Ventilator-associated tracheobronchitis. The impac of target antibiotic therapy on patient outcomes. *Chest* **2009**, *135*, 521–528.
16. Torres, A.; Ewig, S.; Lode, H.; Carlet, J.; European HAP Working Group. Defining, treating and preventing hospital acquired pneumonia: European perspective. *Intensive Care Med.* **2009**, *35*, 9–29.
17. Rodríguez, A.; Póvoa, P.; Nseir, S.; Salluh, J.; Curcio, D.; Martín-Loeches, I. Incidence and diagnosis of ventilator-associated tracheobronchitis in the intensive care unit: An international online survey. *Crit. Care* **2014**, *18*, R32.
18. Nseir, S.; Martin-Loeches, I.; Makris, D.; Jaillette, E.; Karvouniaris, M.; Valles, J.; Zakynthinos, E.; Artigas, A. Impact of appropriate antimicrobial treatment on transition from ventilator-associated tracheobronchitis to ventilator-associated pneumonia. *Crit. Care* **2014**, *18*, R129.
19. Palmer, L.B.; Smaldone, G.C.; Chen, J.J.; Baram, D.; Duan, T.; Monteforte, M.; Varela, M.; Tempone, A.K.; O’Riordan, T.; Daroowalla, F.; et al. Aerosolized antibiotics and ventilator-associated tracheobronchitis in the intensive care unit. *Crit. Care Med.* **2008**, *36*, 2008–2013.
20. Agrafiotis, M.; Siempos, I.I.; Falagas, M.E. Frequency, prevention, outcome and treatment of ventilator-associated tracheobronchitis: Systematic review and meta-analysis. *Respir. Med.* **2010**, *104*, 325–336.
21. Douglas, I.S.; Price, C.S.; Overdier, K.H.; Wolken, R.F.; Metzger, S.W.; Hance, K.R.; Howson, D.C. Rapid automated microscopy for microbiological surveillance of ventilator-associated pneumonia. *Am. J. Respir. Crit. Care Med.* **2015**, *191*, 566–573.
22. Bos, L.D.; Weda, H.; Wang, Y.; Knobel, H.H.; Nijsen, T.M.; Vink, T.J.; Zwinderman, A.H.; Sterk, P.J.; Schultz, M.J. Exhaled breath metabolomics as a noninvasive diagnostic tool for acute respiratory distress syndrome. *Eur. Respir. J.* **2014**, *44*, 188–197.

## Treatment of HAP/VAP: New Therapeutic Options

VallecocciaM.S.De PascaleG.AntonelliM.Department of Anesthesiology and Intensive Care Medicine, Catholic University of Rome, Fondazione Policlinico Universitario “A. Gemelli”, Italy

**Abstract:** Hospital acquired pneumonia (HAP) and ventilator-associated pneumonia (VAP) are the most common complications of the hospital stay, accounting for 22% of all health care-associated infections in a multistate point-prevalence survey [1]. Despite our best efforts in prevention bundles, they often involve multidrug-resistant (MDR) germs, account for up to 50% of antibiotic prescription and have a negative impact on patient outcome, raising the average cost per patient stay and prolonging the length of mechanical ventilation and hospitalization [2].

The sub-therapeutic lung penetration of intravenous (IV) antibiotics and the need of prolonged antibiotic therapy is one of the basis of the spread of multi-drug MDR pathogens during VAP, favouring the selection of resistant bacteria [3]. For this reason, the use of aerosolized antibiotics was considered to optimize the delivery of antibiotics to the infection site, reducing systemic side effects [4]. Furthermore, the last ATS/IDSA guidelines suggest adjunctive inhaled colistin in patients with HAP/VAP caused by carbapenem-resistant pathogens or Acinetobacter species sensitive only to polymyxins (weak recommendation, low-quality evidence) [5]. In a recent study that took place in our ICU, we evaluated the effect of a combined aerosolized-IV colistin therapy versus IV colistin alone on the outcomes of microbiologically documented VAP caused by colistin-only susceptible gram-negative bacteria. Patients treated with the combined therapy had higher clinical cure rate and ventilator-free days, without any differences in all-cause ICU mortality, length of ICU stay or rate of acute kidney injury. Interestingly, eradication of the causative organism was also more common in the combined treatment group [6]. There are other studies about the use of the combined IV/aerosolized therapy that show improved clinical cure rate and a trend toward lower mortality, but a conclusive opinion about the efficacy cannot be stated because of the high level of heterogeneity. Different types of nebulizers are used and clinical and technical limitations cause suboptimal delivery of nebulized therapy (spontaneous ventilator modes, the presence of heat and moisture exchanges during the treatment etc.). Initial treatment with aerosolized antibiotics combined with IV therapy is therefore a promising treatment strategy that could improve clinical outcomes, but high-quality studies are warranted to become more confident in the effects of inhaled plus intravenous antibiotics [7].

Another weapon in the fight against HAP and VAP are the new released antimicrobials. In a recently published randomized controlled trial, Ceftazidime/avibactam was not inferior to meropenem, providing a potential future alternative to carbapenem in nosocomial pneumonia caused by Gram-negative pathogens [8]. Intensivists are still waiting for the publication of the TANGO-2 trial, a Phase 3 multi-center randomized open-label trial that evaluate the efficacy and safety of Meropenem-Vaborbactam compared to best available therapy (BAT) (NCT 02168946). The preliminary results of the study were presented at the IDWeek convention (in San Diego, CA, in October 2017; abstract 1867). A total of 72 patients with severe infections including nosocomial pneumonia were enrolled, 43 of them had a baseline Carbapenem-resistant Enterobacteriaceae. The trial was stopped prematurely for significant superiority in terms of clinical failure, nephrotoxicity and non-significant improvement of day-28 mortality of patients with HAP/VAP. Also Temocillin, a ticarcillin derivative that resists ESBL, can be used in the treatment of HAP/VAP but only as a step-down therapy for pathogens with MICs below 8 mg/L, confirming its potential application as a carbapenem-sparing agent [9].

Unfortunately, the number of new antibiotics released onto the market remains limited so there is the need of developing innovative approach for the management of MDR infections. In the future, a valuable complement to standard antibiotic therapy in treatment of HAP/VAP may be the passive immunotherapy. The clinical benefits of monoclonal antibodies (MAbs) has been established in several therapeutic areas and especially in patients with more severe disease as an adjunct to standard treatment. Therefore, a combination between antimicrobials and MAbs might lead to a more rapid resolution of infections, shorter ICU length of stay, decrease in mortality, morbidity and health care costs even if the cost of MAbs remain high.

Panobacumab is fully human monoclonal antibody of the IgM/κ isotype directed against the LPS O-polysaccharide moiety of P. aeruginosa serotype IATS O11. Its safety and efficacy has been demonstrated in humans [10]. A small phase IIa study about the use of this monoclonal antibody as complementary strategy of treating HAP was recently published, suggesting a potential efficacy in improving the clinical outcome of patients treated with this MAb [11].

Pagibaximab, a human chimeric monoclonal antibody developed against lipoteichoic acid (LTA), has been developed and successfully tested in a phase 2, randomized double-blind trial about the prevention of staphylococcal sepsis of preterm neonates [12]. No clinical studies regarding adult patients are available so far. There are also some interesting data about the development of fully human IgG1 MAbs that bind polysaccharide structures of different Gram-positive bacteria, making possible their elimination by antibody-mediated complement deposition and phagocytosis. The strong opsonizing activity was demonstrated against two different enterococcal and two different staphylococcal strains, including a MDR Staphylococcus aureus (MRSA) [13].

Passive immunotherapy with preformed monoclonal antibody allow an immediate protection of the patients, while active immunization requires often booster dosages because the host needs time to develop and produce its own protective antibodies. Therefore, the use of monoclonal antibodies (mAbs) could be advantageous because it enables prophylaxis of patients at particular risk, such as patients in intensive care units, patients after organ or bone-marrow transplantation, patients after implantation of foreign materials (e.g., artificial heart valves or artificial joints), or after chemotherapy. The proposed treatment and/or prophylaxis with a monoclonal antibody will probably complement rather than replace current treatment regimens and may act synergistically with antibiotic therapy.

Although promising, additional in vitro studies and better clinical trials are needed to establish monoclonal antibodies as therapeutic and/or prophylactic strategies against MDR pathogens. Additional evidences are required to understand the role of passive immunotherapy for treating severe infections, including HAP/VAP infections.

**References**

1. Magill, S.S.; Edwards, J.R.; Bamberg, W.; Beldavs, Z.G.; Dumyati, G.; Kainer, M.A.; Lynfield, R.; Maloney, M.; McAllister-Hollod, L.; Nadle, J.; et al. Multistate point-prevalence survey of health care-associated infections. *N. Engl. J. Med.* **2014**, *370*, 1198–1208, doi:10.1056/NEJMoa1306801.
2. Weiss, E.; Essaied, W.; Adrie, C.; Zahar, J.R.; Timsit, J.F. Treatment of severe hospital-acquired and ventilator-associated pneumonia: A systematic review of inclusion and judgment criteria used in randomized controlled trials. *Crit. Care* **2017**, *21*, 162, doi:10.1186/s13054-017-1755-5.
3. Weiner, L.M.; Webb, A.K.; Limbago, B.; Dudeck, M.A.; Patel, J.; Kallen, A.J.; Edwards, J.R.; Sievert, D.M. Antimicrobial-Resistant Pathogens Associated with Healthcare-Associated Infections: Summary of Data Reported to the National Healthcare Safety Network at the Centers for Disease Control and Prevention, 2011-2014. *Infect. Control Hosp. Epidemiol.* **2016**, *37*, 1288–1301, doi:10.1017/ice.2016.174.
4. Kollef, M.H.; Hamilton, C.W.; Montgomery, A.B. Aerosolized antibiotics: Do they add to the treatment of pneumonia? *Curr. Opin. in Infect. Dis.* **2013**, *26*, 538–544.
5. Kalil, A.C.; Metersky, M.L.; Klompas, M.; Muscedere, J.; Sweeney, D.A.; Palmer, L.B.; Napolitano, L.M.; O’Grady, N.P.; Bartlett, J.G.; Carratala, J.; et al. Management of Adults With Hospital-acquired and Ventilator-associated Pneumonia: 2016 Clinical Practice Guidelines by the Infectious Diseases Society of America and the American Thoracic Society. *Clin. Infect. Dis.* **2016**, *63*, e61–e111, doi:10.1093/cid/ciw353.
6. Tumbarello, M.; De Pascale, G.; Trecarichi, E.M.; De Martino, S.; Bello, G.; Maviglia, R.; Spanu, T.; Antonelli, M. Effect of aerosolized colistin as adjunctive treatment on the outcomes of microbiologically documented ventilator-associated pneumonia caused by colistin-only susceptible gram-negative bacteria. *Chest* **2013**, *144*, 1768–1775, doi:10.1378/chest.13-1018.
7. Valachis, A.; Samonis, G.; Kofteridis, D.P. The role of aerosolized colistin in the treatment of ventilator-associated pneumonia: A systematic review and metaanalysis. *Crit. Care Med.* **2015**, *43*, 527–533.
8. Torres, A.; Zhong, N.; Pachl, J.; Timsit, J.F.; Kollef, M.; Chen, Z.; Song, J.; Taylor, D.; Laud, P.J.; Stone, G.G.; et al. Ceftazidime-avibactam versus meropenem in nosocomial pneumonia, including ventilator-associated pneumonia (REPROVE): A randomised, double-blind, phase 3 non-inferiority trial. *Lancet Infect. Dis.* **2017**, doi:10.1016/s1473-3099(17)30747-8.
9. Balakrishnan, I.; Awad-El-Kariem, F.M.; Aali, A.; Kumari, P.; Mulla, R.; Tan, B.; Brudney, D.; Ladenheim, D.; Ghazy, A.; Khan, I.; et al. Temocillin use in England: Clinical and microbiological efficacies in infections caused by extended-spectrum and/or derepressed AmpC beta-lactamase-producing Enterobacteriaceae. *J. Antimicrob. Chemother.* **2011**, *66*, 2628–2631, doi:10.1093/jac/dkr317.
10. Lazar, H.; Horn, M.P.; Zuercher, A.W.; Imboden, M.A.; Durrer, P.; Seiberling, M.; Pokorny, R.; Hammer, C.; Lang, A.B. Pharmacokinetics and Safety Profile of the Human Anti-Pseudomonas aeruginosa Serotype O11 Immunoglobulin M Monoclonal Antibody KBPA-101 in Healthy Volunteers ∇. *Antimicrob. Agents Chemother.* **2009**, *53*, 3442–3446, doi:10.1128/aac.01699-08.
11. Que, Y.A.; Lazar, H.; Wolff, M.; Francois, B.; Laterre, P.F.; Mercier, E.; Garbino, J.; Pagani, J.L.; Revelly, J.P.; Mus, E.; et al. Assessment of panobacumab as adjunctive immunotherapy for the treatment of nosocomial Pseudomonas aeruginosa pneumonia. *Eur. J. Clin. Microbiol. Infect. Dis.* **2014**, *33*, 1861–1867, doi:10.1007/s10096-014-2156-1.
12. Weisman, L.E.; Thackray, H.M.; Steinhorn, R.H.; Walsh, W.F.; Lassiter, H.A.; Dhanireddy, R.; Brozanski, B.S.; Palmer, K.G.; Trautman, M.S.; Escobedo, M.; et al. A randomized study of a monoclonal antibody (pagibaximab) to prevent staphylococcal sepsis. *Pediatrics* **2011**, *128*, 271–279, doi:10.1542/peds.2010-3081.
13. Rossmann, F.S.; Laverde, D.; Kropec, A.; Romero-Saavedra, F.; Meyer-Buehn, M.; Huebner, J. Isolation of highly active monoclonal antibodies against multiresistant gram-positive bacteria. *PLoS ONE* **2015**, *10*, e0118405, doi:10.1371/journal.pone.0118405.

##                                        

 

SESSION X. “NEW ANTIBIOTICS FOR TREATMENT OF SEVERE INFECTIONS”

 

## New Antibiotics for Treatment of Severe Infections. Ceftazidime-Avibactam vs. Meropenem

BassettiMatteoVenaAntonioPeghinMaddalenaRighiEldaCastaldoNadiaInfectious Diseases Clinic, Department of Medicine, University of Udine and Azienda Sanitaria Universitaria Integrata, Presidio Ospedaliero Universitario Santa Maria della Misericordia, Udine, Italy

**Abstract:** Despite the implementation of intensive care bundles, the incidence of severe infections due to multidrug-resistant (MDR) pathogens has dramatically escalated over the last decade, becoming an independent predictor of initial inadequate antibiotic therapy and increased mortality among critically ill patients [1]. The most worrying emerging threats associated with antibiotic resistance are the gram negative (GN) pathogens included in the ESCAPE group (*Enterococcus faecium*, *Staphylococcus aureus*, *Clostridium difficile*, *Acinetobacter species*, *P. aeruginosa*, and *Enterobacteriaceae*), which represent a major challenge in the context of health care acquired infections [2]. Due to the shortage of effective therapeutic options, empirical treatment in this setting has been traditionally based on the rediscovery of old drugs, such as antipseudomonal β-lactams, fosfomycin, aminoglycosides and polymixins, often used in combination, with an increased risk of drug-related toxicity and emerge of new resistance [3]. The recent introduction in the antibiotic pipeline of new molecules with broad-spectrum activity against MDR species has enhanced the therapeutical alternatives for critical setting, where GN bacteria represent a serious threat [4]. Ceftazidime–avibactam is a novel agent combining a third generation cephalosporin (ceftazidime) and a β-lactamase inhibitor (avibactam), which has been demonstrated to inactivate a wide range of β-lactamases, including Ambler class A (ESBL and KPC), AmpC, and some class D serine beta-lactamases [5]. Ceftazidime-avibactam combination shows highly in vitro efficacy against cephalosporin-resistant *Enterobacteriaceae* and *P. aeruginosa*, while has a very limited activity against *A. baumannii*, other anaerobic Gram-negative rods, metallo-β-lactamases and *Acinetobacter* OXA carbapenemases [6]. Ceftazidime–avibactam has currently been approved by Food and drug Administration (FDA) and European Medicines Agency (EMA) for the treatment of complicated urinary tract infections (cUTIs), complicated intra-abdominal infections (cIAIs), hospital acquired pneumonia/ventilator associated pneumonia (HAP/VAP) [7]. Beyond the FDA-approved indications, ceftazidime-avibactam is also considered as an alternative agent for the treatment of GN infections when traditional agents are not available because of antimicrobial resistance, clinical failure, or toxicity [8]. Ceftazidime-avibactam shows an interesting bactericidal activity, a linear pharmacokinetics, similar efficacy and safety compared to carbapenems [9]. A prospective study (REPROVE study) has recently compared ceftazidime-avibactam activity versus meropenem for the treatment of nosocomial pneumonia, including VAP. More than 800 patients were included. The most commonly isolated pathogens were *K. pneumoniae* and *P. aeruginosa*, with 28% of ceftazidime non-susceptible stains. A successful outcome was achieved in 68.8% of cases treated with ceftazidime-avibactam, compared with 72.9% in the group treated with meropenem, The result of REPROVE study confirmed ceftazidime-avibactam non-inferiority to carbapenem, which represent one of the most commonly employed antimicrobial class in ICU [10]. Furthermore no differences in adverse event rates have been recorded between ceftazidime-avibactam and all tested comparators [11]. Currently no randomized trials have still been addressed to the role of ceftazidime-avibactam for treatment of carbapenem-resistant *Enterobacteriaceae* (CRE), although several reports suggest its efficacy in this setting, with overall success rates between 45% and 76% [12]. A recent multi-center, retrospective case series collected 60 cases treated with ceftazidime-avibactam for CRE infection from any source, 45% of which were pneumonia and 38% requiring mechanical ventilation. Overall result of this trial showed a rate of 53% of microbiological cure and 65% of clinical success, thus reproving ceftazidime-avibactam activity in severe CRE respiratory infections [13]. Of note that the development of resistance has already been described in patients who have received Ceftazidime-avibactam. However the relevance of this effect in clinical practice is still unclear [14]. In summary, we suggest that ceftazidime-avibactam can be an interesting treatment option in patients with nosocomial infections, or for empirical therapy in settings with critically ill patients with high rates of ESBL or CRE GN bacteria.

**References**

1. Boucher, H.W.; Talbot, G.H.; Bradley, J.S.; Edwards, J.E.; Gilbert, D.; Rice, L.B.; Scheld, M.; Spellberg, B.; Bartlett, J. Bad bugs, no drugs: No ESKAPE! An update from the Infectious Diseases Society of America. *Clin. Infect. Dis.* **2009**, *48*, 1–12, doi:10.1086/595011.
2. Peterson, L.R. Bad bugs, no drugs: No ESCAPE revisited. *Clin. Infect. Dis.* **2009**, *49*, 992–993, doi:10.1086/605539.
3. Bassetti, M.; Righi, E. Development of novel antibacterial drugs to combat multiple resistant organisms. *Langenbecks Arch. Surg.* **2015**, *400*, 153–165, doi:10.1007/s00423-015-1280-4.
4. Bassetti, M.; Welte, T.; Wunderink, R.G. Treatment of Gram-negative pneumonia in the critical care setting: Is the beta-lactam antibiotic backbone broken beyond repair? *Crit. Care* **2016**, *20*, 19, doi:10.1186/s13054-016-1197-5.
5. Keepers, T.R.; Gomez, M.; Celeri, C.; Nichols, W.W.; Krause, K.M. Bactericidal activity, absence of serum effect, and time-kill kinetics of ceftazidime-avibactam against beta-lactamase-producing Enterobacteriaceae and Pseudomonas aeruginosa. *Antimicrob. Agents Chemother.* **2014**, *58*, 5297–5305, doi:10.1128/AAC.02894-14.
6. Haidar, G.; Clancy, C.J.; Shields, R.K.; Hao, B.; Cheng, S.; Nguyen, M.H. Mutations in blaKPC-3 That Confer Ceftazidime-Avibactam Resistance Encode Novel KPC-3 Variants That Function as Extended-Spectrum beta-Lactamases. *Antimicrob. Agents Chemother.* **2017**, *61*, doi:10.1128/AAC.02534-16.
7. Falcone, M.; Paterson, D. Spotlight on ceftazidime/avibactam: A new option for MDR Gram-negative infections. *J. Antimicrob. Chemother.* **2016**, *71*, 2713–2722, doi:10.1093/jac/dkw239.
8. Connor, K.A. Newer Intravenous Antibiotics in the Intensive Care Unit: Ceftaroline, Ceftolozane-Tazobactam, and Ceftazidime-Avibactam. *AACN Adv. Crit. Care* **2016**, *27*, 353–357, doi:10.4037/aacnacc2016612.
9. Carmeli, Y.; Armstrong, J.; Laud, P.J.; Newell, P.; Stone, G.; Wardman, A.; Gasink, L.B. Ceftazidime-avibactam or best available therapy in patients with ceftazidime-resistant Enterobacteriaceae and Pseudomonas aeruginosa complicated urinary tract infections or complicated intra-abdominal infections (REPRISE): A randomised, pathogen-directed, phase 3 study. *Lancet Infect. Dis.* **2016**, *16*, 661–673, doi:10.1016/S1473-3099(16)30004-4.
10. Torres, A.; Zhong, N.; Pachl, J.; Timsit, J.F.; Kollef, M.; Chen, Z.; Song, J.; Taylor, D.; Laud, P.J.; Stone, G.G.; et al. Ceftazidime-avibactam versus meropenem in nosocomial pneumonia, including ventilator-associated pneumonia (REPROVE): A randomised, double-blind, phase 3 non-inferiority trial. *Lancet Infect. Dis.* **2017**, doi:10.1016/S1473-3099(17)30747-8.
11. Mazuski, J.E.; Gasink, L.B.; Armstrong, J.; Broadhurst, H.; Stone, G.G.; Rank, D.; Llorens, L.; Newell, P.; Pachl, J. Efficacy and Safety of Ceftazidime-Avibactam Plus Metronidazole Versus Meropenem in the Treatment of Complicated Intra-abdominal Infection: Results From a Randomized, Controlled, Double-Blind, Phase 3 Program. *Clin. Infect. Dis.* **2016**, *62*, 1380–1389, doi:10.1093/cid/ciw133.
12. Bassetti, M.; Giacobbe, D.R.; Giamarellou, H.; Viscoli, C.; Daikos, G.L.; Dimopoulos, G.; De Rosa, F.G.; Giamarellos-Bourboulis, E.J.; Rossolini, G.M.; Righi, E.; et al. Management of KPC-producing Klebsiella pneumoniae infections. *Clin. Microbiol. Infect.* **2017**, doi:10.1016/j.cmi.2017.08.030.
13. King, M.; Heil, E.; Kuriakose, S.; Bias, T.; Huang, V.; El-Beyrouty, C.; McCoy, D.; Hiles, J.; Richards, L.; Gardner, J.; et al. Multicenter Study of Outcomes with Ceftazidime-Avibactam in Patients with Carbapenem-Resistant Enterobacteriaceae Infections. *Antimicrob. Agents Chemother.* **2017**, *61*, doi:10.1128/AAC.00449-17.
14. Temkin, E.; Torre-Cisneros, J.; Beovic, B.; Benito, N.; Giannella, M.; Gilarranz, R.; Jeremiah, C.; Loeches, B.; Machuca, I.; Jimenez-Martin, M.J.; et al. Ceftazidime-Avibactam as Salvage Therapy for Infections Caused by Carbapenem-Resistant Organisms. *Antimicrob. Agents Chemother.* **2017**, *61*, doi:10.1128/AAC.01964-16.

## Improving Antibiotic Delivery for Pneumonia

EhrmannStephan^1^,^2^,^3^1Médecine Intensive Réanimation and CIC INSERM 1415, CHRU de Tours, Tours, France.2Centre d’étude des pathologies respiratoires, INSERM U1100, Aérosolthérapie et biomédicaments à visée respiratoire, Université de Tours, Tours, France3CRICS-TriggerSEP European research network

**Abstract:** Pneumonia represents a very frequent infection among critically ill patients. Up to 20% of hospitalized patients with community acquired pneumonia develop severe disease, 10% are admitted to the intensive care unit and mortality remains as high as 30%. Uncontrolled inflammation contributes to sepsis and delayed clinical stabilization driving suboptimal outcome [1,2]. Ventilator associated pneumonia represents the most frequent nosocomial infection among critically ill patients, whereas the direct impact on mortality remains debated, morbidity is very high with increased lengths of stay, disease relapse and multidrug-resistant bacteria selection [3]. Notwithstanding numerous and important differences, severe community acquired and ventilator associated pneumonia share some common pathophysiologic features which leave room to improve antibiotic delivery in order to optimize patients’ outcome. Among others, a high bacterial burden, poor antibiotic diffusion into the lung parenchyma, modified pharmacokinetics favoring low antibiotic drug concentrations or conversely drug toxicity and acute kidney injury driving increased concentrations, practical constraints for clinical and biomarker assessment of disease course all represent challenges for optimal antibiotic delivery. Choice of the antibiotic drug, administration dose and schedule, route of delivery, duration of therapy all represent potential opportunities for improvement. Drug: Whereas the choice is limited to a few antibiotic drugs for empiric therapy of community acquired pneumonia, several options can be discussed when adapting therapy to causative bacteria such as the benefit of macrolide induced immune-modulation to alter Streptococcus pneumoniae induced inflammation [3], use of antibiotics targeting protein synthesis to alter bacterial toxin production. In the setting of nosocomial and healthcare associated pneumonia a large panel of antibiotic drugs may be considered based on patient history and local epidemiology of drug resistance. Aside of efficacy, antibiotic side effects need to be considered, the benefits and harms of nephrotoxic drugs may be discussed. Dose and schedule: A large body of evidence shows that critically ill patients are at risk of insufficient antibiotic drug concentration at the site of action due to increased drug volume of distribution. Furthermore, limited diffusion of some drugs to the lung, the important bacterial burden and large infected tissue volume may further impede optimal drug concentration in the case of pneumonia. Despite the lack of gold standard exogenous glomerular filtration maker studies to investigate the phenomenon, some patients may experience glomerular hyperfiltration further reducing antibiotic concentrations. High doses of concentration dependent antibiotics and continuous infusion of time dependent antibiotics have been evaluated as means to improve pharmacokinetics/pharmacodynamics of therapy [5]. Route: Given the challenge to achieve sufficiently high drug concentrations in the lung after systemic delivery, inhaled antibiotic administration has been the subject of intense investigations. Whereas it appears that antibiotics delivered as an inhaled aerosol enable to successfully treat pneumonia in ventilated critically ill patients, this practice currently lacks high level scientific evidence of benefit compared to standard intravenous therapy [6]. Given the spread of multidrug-resistant bacteria clinicians may face situations where inhaled antibiotic therapy as a complement or substitute to intravenous therapy may represent a suitable way to improve antibiotic therapy. However, inhaled antibiotic therapy implementation appears frequently suboptimal or even dangerous in observational studies of routine care [7,8]. To ensure optimal and safe aerosol delivery in ventilated patients, nebulizer placement, circuit setup, humidification, filter placement, ventilator settings all need to be carefully considered [5]. Duration: After initial antibiotic therapy careful monitoring is required to ascertain patient reach clinical stability and cure. Reduced antibiotic therapy durations may be considered in some cases based on clinical and biomarker evolution such as C reactive protein or procalcitonin. Very short antibiotic therapy may be considered in patients with non-bacterial pneumonia [9]. Conversely patients showing therapeutic failure and/or nosocomial superinfection may require antibiotic escalation.

**References**

1. Prina, E.; Ceccato, A.; Torres, A. New aspects in the management of pneumonia. *Crit. Care* **2016**, *20*, 267.
2. Morley, D.; Torres, A.; Cilloniz, C.; Martin-Loeches, I. Predictors of treatment failure and clinical stability in patients with community acquired pneumonia. *Ann. Transl. Med.* **2017**, *5*, 443.
3. Kalil, A.C.; Metersky, M.L.; Klompas, M.; Muscedere, J.; Sweeney, D.A.; Palmer, L.B.; Napolitano, L.M.; O’Grady, N.P.; Bartlett, J.G.; Carratala, J.; et al. Management of adults with hospital-acquired and ventilator associated pneumonia: 2016 Clinical practice guidelines by the Infectious Diseases Society of America and the American Thoracic Society. *Clin. Infect. Dis.* **2016**, *63*, e61–e111.
4. Kovaleva, A.; Remmelts, H.H.; Rijkers, G.T.; Hoepelman, A.I.M.; Biesma, D.H.; Oosterheert, J.J. Immunomodulatory effects of macrolides during community-acquired pneumonia: A literature review. *J. Antimicrob. Chemother.* **2012**, *67*, 530–540.
5. Roberts, J.A.; Abdul-Aziz, M.H.; Davis, J.S.; Dulhunty, J.M.; Cotta, M.O.; Myburgh, J.; Bellomo, R.; Lipman, J. Continuous versus intermittent beta-lactam infusion in severe sepsis. A Meta-analysis of individual patient data from randomized trials. *Am. J. Respir. Crit. Care Med.* **2016**, *194*, 681–691.
6. Ehrmann, S.; Chastre, J.; Diot, P.; Lu, Q. Nebulized antibiotics in mechanically ventilated patients: A challenge for translational research from technology to clinical care. *Ann. Intensive Care* **2017**, *7*, 78.
7. Solé-Lleonart, C.; Rouby, J.J.; Chastre, J.; Poulakou, G.;Palmer, L.B.; Blot, S.; Felton, T.; Bassetti, M.; Luyt, C-E.; Pereira, J.M. et al. Intratracheal administration of antimicrobial agents in mechanically ventilated adults: An international survey on delivery practices and safety. *Respir. Care* **2016**, *61*, 1008–1014.
8. Ehrmann, S.; Roche-Campo, F.; Bodet-Contentin, L.; Razazi, K.; Dugernier, J.; Trenado-Alvarez, J.; Donzeau, A.; Vermeulen, F.; Thévoz, D.; Papanikolaou, M. et al. Aerosol therapy in intensive and intermediate care units: Prospective observation of 2808 critically ill patients. *Intensive Care Med.* **2016**, *42*, 192–201.
9. Hurst, J.M.; Bosso, J.A. Antimicrobial stewardship in the management of community-acquired pneumonia. *Curr. Opin. Infect. Dis.* **2013**, *26*, 184–188.

## International Actions against Anti-Microbial Resistance (AMR)

CarletJeanPresident of the World Alliance Against Antibiotic Resistance (WAAAR)

**Abstract:** The discovery of antibiotics has been undoubtedly the most important one in medicine. Those compounds saved a huge number of patients, and more recently allowed determinant progresses in medicine like treatment of neonatal sepsis, development of neonatal and adult ICUs, transplantations, treatment of hematological disorders, bone marrow transplantation, antibioprophylaxis in surgery, to cite only a few. Resistance to antibiotics appeared very quickly after their discovery, as already mentioned by Fleming for penicillin. Many antibiotics from various classes were discovered over years, until recently. We have not been able to protect this precious treasure, unconscious of the risk, and certain that new antibiotics would be discovered forever [1]. Unfortunately no antibiotics from new classes were discovered in the last 20 years, and rather few are expected in the near future. In the meanwhile, resistance to antibiotics increased sharply in most countries, mostly but not only due to the over-consumption of those compounds. Some countries like Scandinavian ones, Iceland, The Netherlands were able to control the use of antibiotics, and kept over years a very low level of resistance [2]. Other factors are also very important. The transmission of resistant bugs, mostly via the hands, is a paramount factor, in both the hospitals and the community. The role of the environment is also important, although we do not know to which extent.

Since several years strong international actions have been implemented. Several NGOs are very active in this field (WAAAR, React, CDDEP, GARP, Antibiotic Action, APUA …). Common actions between those NGOs have been also organized recently (CARA). International agencies have also set up very offensive programmes, in particular WHO, OIE, FAO, European commission, United nations, CDC, ECDC. Several countries have also very strong national programmes, in particular the USA, Germany, UK, France, Scandinavian countries, and several Asian ones including China. WHO is very logically the structure which has the natural power and duty to co-ordinate those different actions, but we can easily understand that it is pretty complex.

Europe has also very effective networks to follow antibiotic resistance (EARSS from ECDC) or antibiotic consumption (ESAC from ECDC).

We will see in the next few years if those international actions and programs will be effective, in order to reduce antibiotic resistance

**References**

1. Carlet, J.; Collignon, P.; Goldmann, D.; Goossens, H.; Gyssens, I.C.; Harbarth, S.; Jarlier, V.; Levy, S.B.; N’Doye, B.; Pittet, D.; et al. Society failure to protect a precious resource: Antibiotics. *Lancet* **2011**, *378*, 369–371, doi:10.1016/S0140–6736(11)60401–7.
2. ECDC. EARSS Programme.

## Posters Presentations

                                       

 

## Infections Associates With Devices in ICU

Molina de la TorreM. CarmenVela ColmeneroR. MaríaRuíz GarcíaM. IsabelSalvagoF. Javier BreaICU Department, University Hospital of Jaén, Spain

Introduction: It is the infection that occurs in a patient with an invasive device (for example a ventilator or a central catheter) that was used within 48 h before the start of the infection. Objectives: Present the infection rates adquired in the ICU of the Hospital Médico Quirúrgico de Jaén related to devices: intubation/mechanical ventilation (MV), urethral catheter (SU) and vascular catheter (CV) during the years 2014 and 2015. Methods: Incidence study, prospective and local level. The Controlled infections have been: pneumonias related to VM (N-VM), SU-related urinary tract infection (UI-SU), bacteremia secondary to infection of central vascular catheters (BS-CVC). The rates are expressed in incidence density (ID) per 1000 days of the risk factor and severity has been calculated with the severity scale APACHE II. The data has been obtained from the National Surveillance Study of Nosocomial Infection (ENVIN-HELICS). The results are presented for quantitative variables such as means and deviation standard and for qualitative variables as percentages for different categories. Results: 839 patients admitted to the ICU MedicalSurgical Hospital Complex of Jaén, consisting of 15 beds, during the study period. The average age was 61.01 (DE ± 15.33). 66.75% have been males. The basic pathology has been: medical (54.54%), coronary (34%), surgical (13.71%) and traumatological (1.67%). THE average APACHE II was 14.07 (SD ± 8.98). The average stay it is of 8.92 days (SD ± 13.16) and the overall mortality of 17.61%. The days of risk, number of infections and ID rates for infection are the following: N-VM: 5450 days of VM, 46 N-VM, 8,44 N-VM/1000 days of VM. IU-SU: 6594 days of SU, 38 IU-SU, 5,76 IU-SU/1000 days of SU. BS-CVC: 5589 days of CVC, 15 BS-CVC, 2.68 BS/1000 days of catheter Central venous. Conclusions: We have identified the rates of adquired infections in ICU during the years 2014 and 2015 of our hospital, what we allows us to compare our results with the data obtained Nacional level.

## Changes in the Multidrug-Resistant Pattern in the Icu of a Tertiary Hospital in Spain in the Last Six Years

Molina de la TorreM.C.Vela ColmeneroR.M.Ruiz GarcíaM.I.Guerrero MarínM.ResinaGordilloM.M. Machado CasasJ.F.Unidad de Cuidados Intensivos Médico Quirúrgicos, Hospital de Jaén, Spain

**Introduction:** The increasing problem of antimicrobial resistence (AMR) in the intensive care units, constitutes a serius threat to public health of global reach, as the World Health Organitation (WHO), the European Centre for Disease Control and Prevention, and others national organizations have recently warned.

As the ANTARTICA (ANTimicrobiAl Resistance CriTIcal CAre) coalition has remarked in Brussels (Nov 2017), AMR is associated with increased mortality, increased costs, prolonged length of stay, and increased antibiotic use.

Multidrug-Resistant organisms (MDROs) are defined as those microorganisms, which are resistant to at least one agent in three or more antimicrobials, usually used in the treatment of infections caused by said microorganism. Such resistance must have clinical and epidemiological relevance.

**Purpose:** The purpose of this study was to identify and to describe the MDR map in our ICU and its evolucion among the last six years (from 2012 to 2017), as well as analyze several epidemiological and clinical factors associated.

**Methods:** This is a retrospective study including all patients admitted from from 2012 to 2017 in the ICU in a tertiary hospital in Jaén, Spain, by using the databases of the ENVIN-HELICS registry (we collected data from 1 April to 30 June of each year). Data concerning demographic characteristics and clinical history were collected from each patient. We analysed the MDR bacteria infection and colonization cases in our ICU, the results was compared with the data in Spain. We used the MDR pathogens cataloged as such by “Resistencia-Zero” project, wich are: *methicillin-resistant Staphylococcus aureus (MRSA), vancomycin-resistant Enterococcus Faecium, extended-spectrum beta-lactamase-producing Enterobacteriacecae, carbapenem-hydrolyzing Gramm Negative Bacteria, Imipenem-R Acinetobacter Baumannii and MDR Pseudomonas Aeruginosa.* We do also analysed the percentage use of several antibiotics, as well as its possible association with resistance map changes.

**Results**: There were 786 patiens with infection by MDROs in these periods, of these, 68.57% were male. The length of stay in ICU was 8.11 days. The average age was 62.73 years, with an APACHE II average of 13.48. The global mortality in these patiens was 18.83%.

A situation of a very high rate of Imipenem-Resistant *Acinetobacter* was detected, we could say this is an *endemic* infection in our unit. This situation changes in 2016 when the number of cases decreases, although a high percentage of cases still persists in comaration with the data collected nationally. In the same year there was a marked increase in the appearance of *Enterobacteriaceae ESBL*, wich remains until now, being similar to the national registry. A decrease of more than 50% in carbapenem use is also detected this year (from 2012 to 2015 meropenem is used in 12% of infections, while in 2016 is used in 4.93% of the infections).

Our *MRSA* cases are anecdotal, and the frecuency of infections caused by this microorganism is much lower than in the rest of Spain.

Also noteworthy is the absence of Vancomicin resistant *Enterococcus.*

In 2017 *Carbapenem-R GN* makes its appearance on stage, a fact that we intent to analyse later.
**2012****2013****2014****2015****2016****2017*****MRSA***2 (9.52%)*1 (10%)4 (22%)2 (5.51%)1 (4.5%)3 (20%)***Enterocc Vanco-R***000000***MDR-Pseudom.A***3 (14%)1 (10%3 (16%)4 (11%)3 (13%)1 (6.6%)***Acinetob. Imipenem-R***13 (62%)6 (60%)9 (50%)18 (50%)7 (**31%**)3 (**20%**)***Enterobact.(ESBL)***3 (14%)2 (20%)2 (11%)12 (33%)11 (**50%**)7 (**46%**)***Carbapenem-R GN***000001 (**6.61%**)Total211018362215(* Percentages referring to the total of the different microorganisms responsible for MDR infections).

**Conclusions:** This study shows that in the last two years there has been a turnaround in the pattern of antimicrobial resistance in our intensive care unit, which consists in a drastic decrease in cases of *Acinetobacter* R infection, and an increase in infections by *Enterobacteriaceae ESBL* (the same data were found also in the national registry). We think it be related to a decrease use of carbapenem, as well as the aplication of selective decontamination of the digestive tract (SDD).

The frecuency of infections by the rest of the multidrug-resistant microorganisms analysed remains practically unchanged or with minimal changes over the years. It is remarkable the appereance in this year of *Carbapenem-R GN.* This is an alarming fact, wich we should analyse in the future. We consider that knowing own patterns, their changes and relationed factors is esential to prevent and control AMR, and may help to reduce the burden of MDR in the ICUs.

## Could Thymoquinone Be the Panacea for Sepsis?

AlkharfyKhalid M.AhmadAjazJanBasit L.Department of Clinical Pharmacy, King Saud University, Riyadh, Saudi Arabia

**Introduction**

Despite many advances in critical care medicine, sepsis remains associated with high mortality rates in intensive care units. Thymoquinone is a naturally occurring small molecule, which has demonstrated plethoric pharmacological properties including anti-inflammatory, anti-oxidant and immune-modulatory activities.

**Objective**

The objective was to evaluate whether thymoquinone administration after the incidence of sepsis can potentially reduce systemic inflammation and improve animal survival rate.

**Methods**

Mice were treated with thymoquinone (1.0 mg/kg i.p.) and subsequently challenged with endotoxin or live *Escherichia coli*. Survival, organ function, vascular homeostasis and early-stage sepsis biomarkers including C-reactive protein (CRP), vascular endothelial growth factor (VEGF), and endothelial cell-specific molecule-1 (ESM-1 or Endocan-1) and inflammatory cytokines (TNF-α, IL-1α, IL-2, IL-6, and IL-10) were evaluated. The outcome of thymoquinone therapy on sepsis mortality was also assessed following a cecal ligation and puncture (CLP) procedure, a widely used and a clinically relevant model of sepsis.

**Results**

Animals treated with thymoquinone showed 80–90% survival rate and improved organ function compared to the control group. Thymoquinone treatment was associated with less nitric oxide (NO) production and lower oxidative stress along with adjusting vascular responsiveness, which is often dysregulated during sepsis and septic shock. There was a significant amelioration of the kidney and liver functions as well as tissue injury. Thymoquinone administration led to a reduction in the synthesis of sepsis-related inflammatory cytokines (TNF-α, IL-1α, IL-2, IL-6, and IL-10) and early stage biomarkers of the bacterial infection (CRP, VEGF, and ESM-1) with ~30–50% fall in their circulating levels.

**Conclusions**

The involvement of thymoquinone against sepsis or septic shock in the governance of its protective activity further support the need to develop it as a more effective therapy. Consequently, the time is probably appropriate to move thymoquinone from experimentation in the laboratory to clinical testing.

## Critical Care Discharges: Improving the Governance and Quality of Clinical Handover via the Development of an Electronic Discharge Proforma and Integration with Electronic Healthcare Records

ClementsJamieShevlinClaireIntensive Care Unit, Craigavon Area Hospital, 63 Lurgan Road, Portadown, BT63 5QQ, Northern Ireland

**Introduction**

Patients leaving critical care areas tread a precarious clinical course and suboptimal clinical handover may lead to deficiencies in care following discharge from ICU/HDU. Electronic discharge summaries have been shown to improve the transfer of clinical information between teams and avoid preventable harm during this high risk event. The National Institute of Clinical Excellence (NICE) have outlined a set of key criteria for inclusion during handover of patients from critical care areas, stating that verbal handover should be augmented by a structured written summary and clinical plan. Anecdotally, the practice of hand-written discharge summaries from the ICU in our centre lacked some of these criteria and failed to reflect the complexity of care delivered, posing a latent risk to the safety of vulnerable patients during transfer from critical care.

**Objectives**

(1) Audit the compliance of hand-written discharge summaries with NICE guidelines, specifically;
  - Reason for admission and primary diagnosis
  - Summary of admission, monitoring, investigations and management
  - Summary of patients awareness/family awareness of condition and treatment
  - Plan for ongoing treatment including;
    - Drugs
    - Nutrition
    - Infection status
    - Limitations of treatment
    - Lines and drains
    - Risks post-discharge and re-escalation plan
  - Documented verbal handover between ICU and receiving team
(2) Devise a template for the completion of electronic discharge summaries and re-audit practice
(3) Facilitate the integration of summaries with Northern Ireland Electronic Care Records (NIECR)

**Methods**

A retrospective audit was carried out (*n* = 20) to identify compliance with discharge standards outlined by NICE. A Plan-Do-Study-Act (PDSA) cycle was undertaken to ascertain the key parameters for inclusion in an electronic critical care discharge proforma and a novel discharge template was designed, circulated and embedded in practice to ensure trainees addressed previously deficient criteria. Liaison with clerical and management staff facilitated integration with electronic healthcare records. Discharge practice was then re-audited following the introduction of this template to compare adherence with discharge criteria.

**Results**

Overall quality of discharge summaries was improved, with increased documentation of previously deficient parameters. Documentation of nutritional status and plans for ongoing nutritional management (65% to 89.5%); limitations of care; predicable risks and plans for re-escalation; patients’ awareness of condition and discussions with family members and ongoing infection risks were all improved (Table 1).

**Conclusions**

Quality, accessibility and traceability of clinical discharge summaries from critical care can be improved via a standardised electronic template, and may be further augmented by integration with electronic healthcare records. Overall, this facilitates improved transfer of clinical information to ward staff and subsequent improvement in the delivery of care. Local practice has been reorganised by this simple project, which is transferable to other hospitals where the discharge process has not been standardised.

## Evaluation of Multiplex PCR in a Regional Hospital Center for Diagnosis of Ventilation-Associated Pneumonia

InesLakbar^1^KarimChaoui^1^Marie-LuceGilbert^1^ClaireChassagne^2^NathalieWilhelm^2^LucileMendes^2^1Intensive Care Unit, Cahors hospital2Microbiology laboratory, Cahors hospital

**Background**: Early availability of information on bacterial pathogens and their antimicrobial susceptibility is key for the management of infectious disease. Currently, using traditional approaches, it usually takes at least 48 h for identification and susceptibility testing of bacterial pathogens. Therefore, the slowness of diagnostic procedures drives prolongation of empiric, potentially inappropriate, antibacterial therapies. Introduction of novel technologies such as molecular biology (PCR) has helped, reducing time to organism identification, optimizing antimicrobial therapy, and subsequently improving clinical outcomes, including mortality rates.

**Objective**: Evaluation of the pertinence of a rapid bacteriologic diagnosis using multiplex PCR Unyvero HPN assay (Curetis^®^) in ventilation associated pneumonia (VAP) in a regional hospital center.

**Material and Method**: This observational, prospective, monocentric trial included patients clinically suspected of VAP and Endotracheal Aspiration (ETA) or Broncho-Alveolar Lavage (BAL) was performed on each patient. Samples were analyzed through conventional microbiologic culture and multiplex PCR test. PCR results were not communicated to clinicians. Descriptive statistical analysis and χ2 tests were performed to evaluate concordance between the two methods.

**Results**: A total of 41 samples were included with 6 (14.6%) negative in both culture and PCR. 7 samples were concordant (ID and resistance mechanisms) between PCR and culture with a faster detection result for PCR. 18 samples were discordant due to reported organisms not being on the PCR panel including 15 contaminants such as coagulase negative staphylococci or Candida and 3 Hafnia alvei. Sequencing is currently ongoing for 3 samples containing bacteria not identified by PCR (Enterobacter cloacae, Proteus and Staphylococcus aureus) and 4 samples where resistance profiles are different. In 11 samples, PCR detected pathogens that were negative in culture because of a pre-existing antibiotherapy in these patients. Preliminary antibiotic courses had no significant impact on PCR results (*p* = 0.662).

**Conclusions**: The use of Multiplex PCR for the rapid diagnosis of VAP allows for an early adaptation of targeted antimicrobial therapy and better stewardship. It would be interesting to add additional pathogens to the panel such as H. alvei. Good knowledge of local trends in aetioly and resistance patterns remains important for accurate diagnosis.

## Figures and Tables

**Figure 1:**
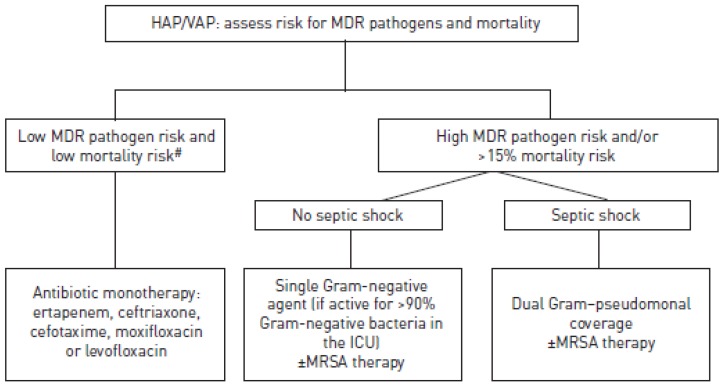
Empiric antibiotic treatment algorithm for hospital-acquired pneumonia [HAP]/ventilator-associated pneumonia [VAP]. MDR: multidrug-resistant, ICU: intensive care unit, MRSA: methicillin-resistant *Staphylococcus aureus.* ^#^: Low risk for mortality is defined as a <15% chance of dying, a mortality rate that has been associated with better outcome using monotherapy than combination therapy when treating serious infections (Kumar A et al., 2010). Reproduced from [1].

**Figure 1:**
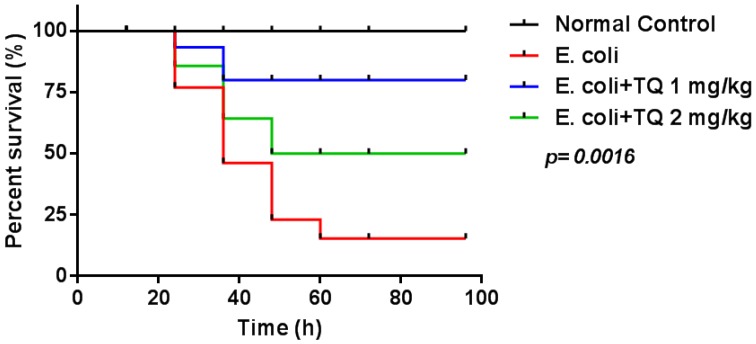
Kaplan–Meier survival plot following *E. coli* (*n* = 12 mice per group). Mice were challenged with live *E. coli* and thymoquinone (TQ) was administered two hours after sepsis induction.

**Figure 2:**
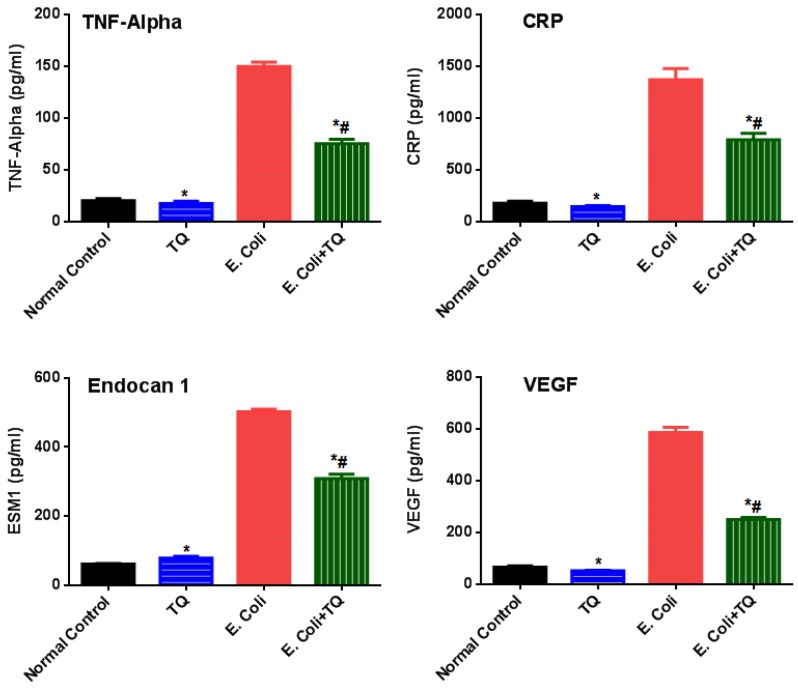
TNF-α, CRP, Endocan-1, VEGF levels in *E. coli*-induced sepsis measured six hours after bacterial challenge in mice treated with thymoquinone (TQ; *n* = 10 mice per group; data is given as mean ± SEM). * (*p* < 0.05) in comparison with Normal Control; ^#^ (*p* < 0.05) in comparison with *E. coli.*

**Table 1:** Monitoring of fluid challenge.

Pulse Pressure Variation STROKE Volume Variation	Patient Should Be Sedated, Sometimes Paralyzed No Cardiac Arrhythmia No Low Lung Compliance No Low Tidal Volume
Passive leg raising test	Tested against blood flow (preferred) or blood pressure changes
End-expiratory occlusion test	Patients should accept a 15 s pause in ventilation. Maybe invalid at a tidal volume of 6 mL/kg
“Mini” fluid challenge: infusion of 100 mL of fluid. Sonography of heart and inferior vena cava (test respiratory variations) (skills needed)	

The first 4 techniques are validated by many studies and meta-analysis. All parameters are limited by sensitivity and specificity ranging from 75% to 95%. Pulse pressure variation 84% (75–90) sensitivity, 80% (78–96) specificity. Stroke volume variation 82% (74–92) sensitivity, 86% (79–93) specificity. Passive leg raising 88% (80–93) sensitivity, 91% (87–96) specificity.

**Table 2:** Cut off and “grey zone” for main parameters.

Pulse Pressure Variation	11 (4–15) mmHg
Stroke volume variation	13 (10–20) mmHg
Passive leg raising (against blood flow)	11 (7–15) mmHg
Inferior vena cava variation (controlled ventilation)	15 (12–21) mmHg

**Table 1:** 

**Audit Criteria**	**% (*n*)**	**% (*n*)**
Reason for admission	100 (20)	100 (19)
Primary diagnosis	100 (20)	100 (19)
Summary of admission, monitoring and management	100 (20)	100 (19)
Summary of investigations	90 (18)	100 (19)
Summary of family discussions	20 (5)	94.7 (18)
A plan for ongoing treatment, including;		
(a) Drugs	100 (20)	100 (19)
(b) Nutrition plan	65 (13)	89.5 (17)
(c) Infection status and plan	60 (12)	94.7 (18)
(d) Limitations of treatment	30 (6)	100 (19)
(e) Lines and drains	85 (17)	100 (19)
(f) Risks post discharge and re-escalation plan	70 (14)	100 (19)
Documented verbal handover between medical teams	90 (18)	94.7 (18)

